# The Evolving Landscape of Antibody–Drug Conjugates: In Depth Analysis of Recent Research Progress

**DOI:** 10.1021/acs.bioconjchem.3c00374

**Published:** 2023-10-11

**Authors:** Janet M. Sasso, Rumiana Tenchov, Robert Bird, Kavita A. Iyer, Krittika Ralhan, Yacidzohara Rodriguez, Qiongqiong Angela Zhou

**Affiliations:** † CAS, A Division of the American Chemical Society, Columbus, Ohio 43210, United States; ‡ ACS International India Pvt. Ltd., Pune 411044, India

## Abstract

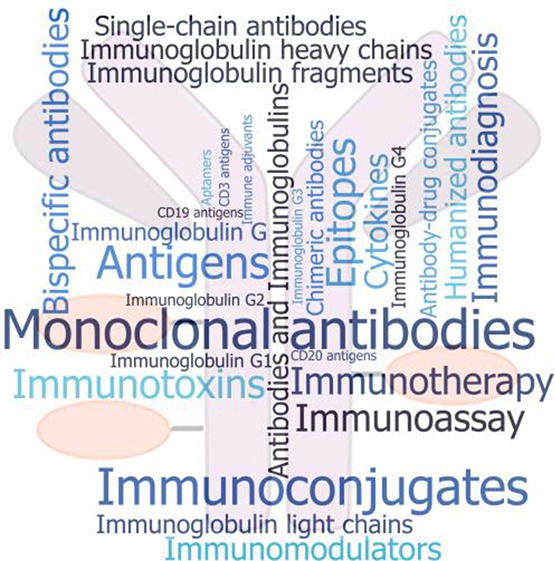

Antibody–drug conjugates (ADCs) are targeted immunoconjugate constructs that integrate the potency of cytotoxic drugs with the selectivity of monoclonal antibodies, minimizing damage to healthy cells and reducing systemic toxicity. Their design allows for higher doses of the cytotoxic drug to be administered, potentially increasing efficacy. They are currently among the most promising drug classes in oncology, with efforts to expand their application for nononcological indications and in combination therapies. Here we provide a detailed overview of the recent advances in ADC research and consider future directions and challenges in promoting this promising platform to widespread therapeutic use. We examine data from the CAS Content Collection, the largest human-curated collection of published scientific information, and analyze the publication landscape of recent research to reveal the exploration trends in published documents and to provide insights into the scientific advances in the area. We also discuss the evolution of the key concepts in the field, the major technologies, and their development pipelines with company research focuses, disease targets, development stages, and publication and investment trends. A comprehensive concept map has been created based on the documents in the CAS Content Collection. We hope that this report can serve as a useful resource for understanding the current state of knowledge in the field of ADCs and the remaining challenges to fulfill their potential.

## 1 Introduction

Antibody–drug conjugates (ADCs) are currently among the most promising drug classes in oncology because of their ability to deliver cytotoxic agents to specific tumor sites, combining the selectivity of monoclonal antibodies (mAbs) and the efficacy of chemotherapeutic drugs.^1−4^ Cancer is a major global health threat, causing millions of fatalities yearly.^5,6^ Chemotherapies based on cytotoxic drugs have been the main strategy for treating of a wide assortment of cancers for decades.^7,8^ Cytotoxic drugs include a large variety of compounds such as alkylating agents, antimetabolites, antitumor antibiotics, topoisomerase and mitotic inhibitors, and corticosteroids among many others.^9−14^ Most of these antitumor drugs, however, exhibit low therapeutic index and cause severe side effects due to nonspecific drug exposure to off-target tissues.^15^ To address these challenges, intense research has been carried out, aimed at developing novel cancer therapeutics with better targeting ability and less harmful side effects.

**Figure 1:**
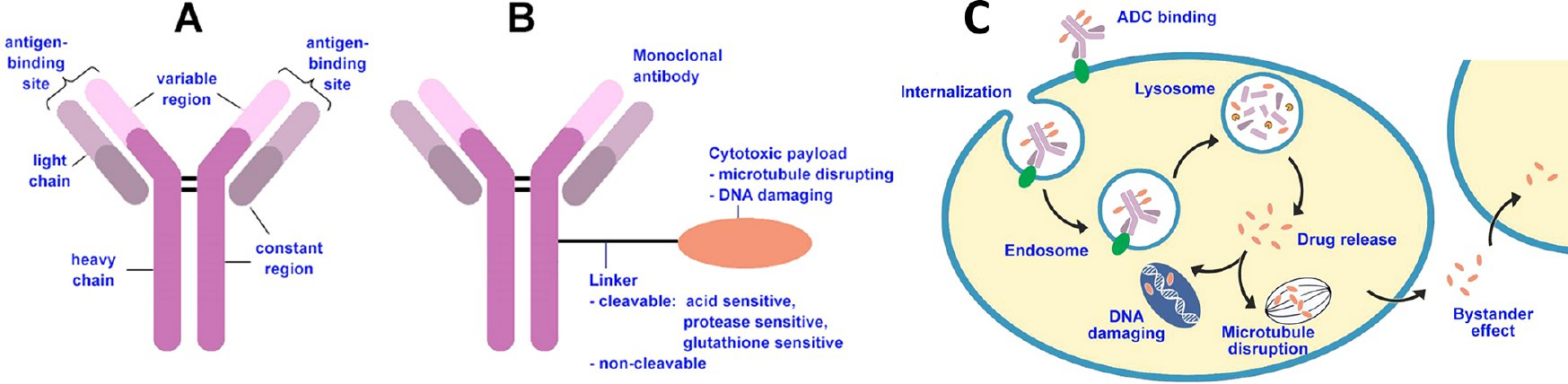
Structure and mechanism of action of ADCs. (A) Scheme of antibody structure including heavy chains, light chains, constant region, variable region, and antigen binding site. (B) Antitumor ADCs combine three key elements: a monoclonal antibody moiety that binds to an antigen preferentially expressed on the tumor cell surface, thereby ensuring specific binding to tumor cells; a covalent linker that warrants that the payload drug is not released in blood ahead of time, but is released within the tumor cell; and a cytotoxic payload that causes tumor cell apoptosis through targeting of key components such as DNA, microtubules. (C) ADC mechanism of action includes key sequential steps: binding to cell surface antigen; internalization of the ADC–antigen complex through endocytosis; lysosomal degradation; release of the cytotoxic payload within the cytoplasm; and interaction with target cell components. A fraction of the payload may be released in the extracellular environment and taken up by neighboring cells, known as the bystander effect.

ADCs are targeted immunoconjugate constructs that integrate the potency of cytotoxic drugs with the selectivity of monoclonal antibodies (Figure 1), minimizing damage to healthy cells and reducing systemic toxicity. The structure of an ADC consists of three main components: a monoclonal antibody, a cytotoxic drug payload, and a linker molecule.^16−19^ The monoclonal antibody is engineered to bind specifically to a target antigen that is overexpressed on cancer cells. This allows the ADC to selectively target cancer cells while sparing normal cells. The cytotoxic drug payload is a potent chemotherapeutic agent that is highly effective in killing cancer cells. The linker molecule attaches the cytotoxic drug to the antibody, and its stability is crucial in controlling the release of the drug within the target cell.^16,20−22^ Once the ADC binds to the cancer cell surface, it is internalized through receptor-mediated endocytosis. Within the cancer cell, the linker molecule is designed to release the cytotoxic drug payload either by enzymatic cleavage or through chemical degradation. Once released, the cytotoxic drug exerts its therapeutic effect by disrupting key cellular processes and inducing cancer cell death.

ADCs offer several advantages over conventional chemotherapy. By selective targeting of cancer cells, ADCs reduce damage to healthy tissues and minimize side effects. This allows for higher doses of the cytotoxic drug to be delivered to the tumor, potentially increasing efficacy. Additionally, the antibody component of ADCs can elicit an immune response against the cancer cells, further enhancing their antitumor activity. Therefore, ADCs have become a major approach in the research and development of antitumor drugs.^16,21,22^

**Figure 2:**
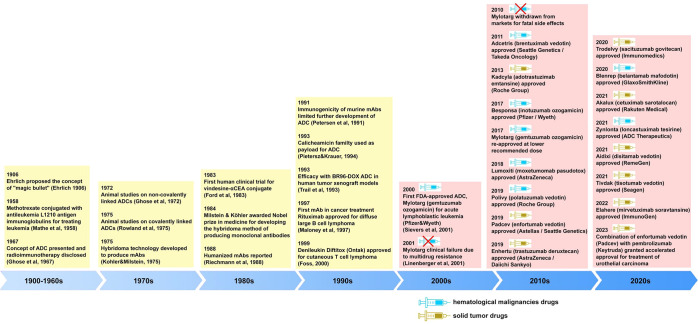
Timeline of key events and discoveries in the antibody–drug conjugate research and development.^21,24−27,29−34,36−80^

The German scientist Paul Ehrlich is credited with coining the term chemotherapy to indicate the use of chemical compounds to treat disease.^23^ Following his experience with antibodies, Ehrlich also proposed the concept of a “magic bullet” (Figure 2) that would bring about a selective targeting of pathogens without injuring the rest of the organism, one of the most notable notions of modern medicine.^24^ Ehrlich’s idea of targeted therapy was first materialized almost 50 years later, with methotrexate linked to an antibody targeting leukemia cells.^25^ The innovative development of hybridoma technology to produce mouse mAbs greatly advanced the field.^26^ It took nearly eight decades until Ehrlich’s visionary concept was advanced to achieve the first human trial of ADC therapy using an anticarcinoembryonic antigen antibody-vindesine conjugate.^27^ Subsequent advances in technologies for the ADC constituent building blocks–the antibody, the linker, and the payload–have resulted in the development of greater number of ADCs with enhanced potency, improved pharmacokinetics, reduced immunogenicity and overall toxicity, and upgraded specificity for cancer cells.^28^ Early ADC prototypes were created but they suffered from issues such as limited stability and inadequate target specificity. In the 1990s preclinical studies demonstrated the potential of ADCs in improving the therapeutic index of cytotoxic drugs by targeting specific antigens expressed on cancer cells.^29−31^ However, early clinical trials encountered challenges including toxicity and insufficient efficacy. In the late 1990s to early 2000s advances in antibody engineering and linker technologies contribute to the development of more stable and effective ADCs.^32−35^ Several ADC candidates have entered clinical trials, showing promising results in terms of efficacy and safety.

The early, first-generation, ADCs used clinically approved drugs with well-documented mechanisms of action, including the antimetabolites methotrexate and 5-fluorouracil, the DNA cross-linker mitomycin and the antimicrotubule agent vinblastine.^30^ First-generation ADC, however, showed insufficient drug efficacy, linker instability, targeted antigens expressed in both normal and cancerous cells, and triggered undesired immune responses.^29^ Further developments, including higher drug efficacy and thoroughly selected targets, eventually led to the first ADC, Mylotarg (gemtuzumab ozogamicin), to get approval from the US Food and Drug Administration (FDA) in 2000 for the treatment of CD33-positive acute myelogenous leukemia.^33,34^ Regardless of the hopeful clinical results, Mylotarg was withdrawn from the market because it did not exhibit progress in overall survival. The second ADC to be developed, Adcetris (brentuximab vedotin), received approval from the US FDA in 2011 for the treatment of Hodgkin’s lymphoma and anaplastic large-cell lymphoma.^43,44^ The next ADC, Kadcyla (trastuzumab emtansine), used a construct combining the humanized antibody trastuzumab with a powerful microtubule inhibitor cytotoxic drug via a highly stable linker. It was approved for the treatment of human epidermal growth factor receptor 2 (HER2)-positive breast cancer in 2013.^45,46^

**Figure 3:**
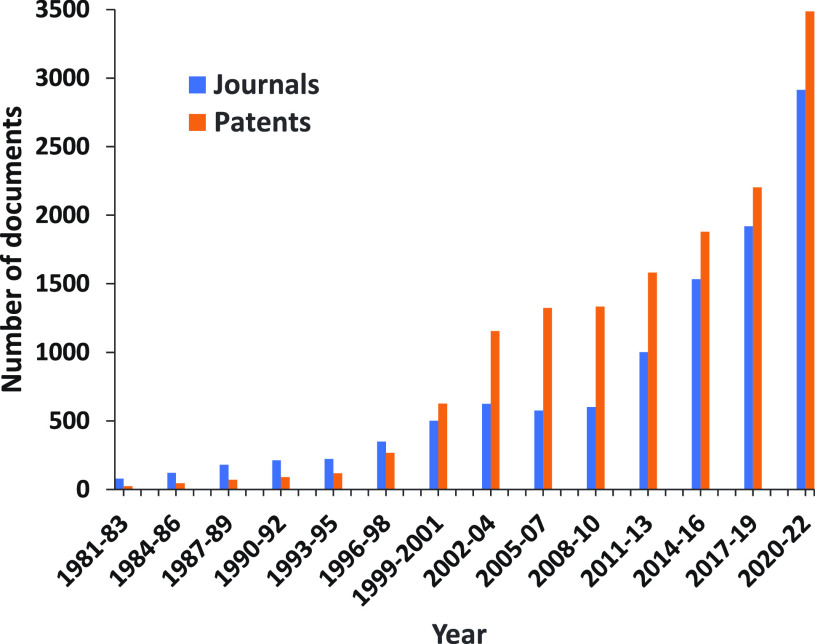
Yearly growth of the number of documents (journal articles and patents) in the CAS Content Collection related to antibody–drug conjugate research and development.

Recent advancements have resulted in a new generation of ADCs with better chemistry, manufacturing, and control (CMC) properties, including linkers with optimized stability and powerful cytotoxic agents. Currently, there are 15 approved ADCs, which have received market endorsement worldwide, with over 150 candidates being investigated in various stages of clinical trials at present. Out of these, ∼12% are in the late-phase (Phase III/IV) owing to rapid advancements in technology required for generating ADCs. Thus, ADCs as an anticancer treatment strategy is leading a new era of targeted cancer defeat. Additionally, ADCs are being increasingly applied in combination with other agents. The growing diversification of target antigens as well as bioactive cytotoxic payloads is rapidly extending the range and scope of tumors targeted by ADCs. However, toxicity continues to remain a key issue in the development of these agents, and a better understanding and management of ADC-related toxicities will be essential for further optimization. Although numerous challenges remain, recent clinical accomplishments have produced intense interest in this therapeutic class reflected in a rapidly growing number of publications (Figure 3). The development of ADCs continues to be a particularly active area of research and development, with ongoing efforts to optimize the design of antibodies, linkers, and cytotoxic drug payloads. The goal is to improve the efficacy and safety profile of ADCs and apply them to a wider range of cancers and to other diseases.

This report provides a detailed overview of the recent advances in antibody–drug conjugate research and considers future directions and challenges in advancing this promising platform to widespread therapeutic use. We examine data from the CAS Content Collection,^81^ the largest human-curated collection of published scientific information, and analyzed the ADC publication landscape of recent research (2000 onward) to uncover the trends in published documents (both journals as well as patents) and to provide insights into scientific advances in the area. We also discuss the evolution of key concepts in the field as well as the major technologies and the development pipelines of ADCs with a particular focus/emphasis on company research, disease targets, and development stages. We hope this report can serve as a useful resource for understanding the current state of the field of ADCs and the remaining challenges to fulfill and achieve their full potential.

## 2 ADC Basics

### 2.1 Selection/Optimization of Antibodies

Antibodies are an essential building block of ADCs (Figure 1) that possess characteristic requirements. It should have a high affinity and avidity for the target antigen but no or insignificant binding to off-target sites and it is important to have selective binding to the target antigen leading to the accumulation and retention of ADCs at the target site.^82−84^ In addition, the antibody should have low immunogenicity, low cross-reactivity, optimum-linker binding, and a long half-life.^85,86^ It is interesting to note that the antibody component of many ADCs retains its activity profile and can therefore interfere with target cell function, alter downstream signaling, and/or cause immune effector cells to elicit payload-independent antitumor immunity, thereby acting beyond mere payload carrier. For example, the Fab region of the antibody in ADCs can disrupt target function by blocking ligand binding, interfering with dimerization and/or endocytosis, and target protein degradation.^84,87^ Likewise, the Fc region of the antibody can induce antibody-dependent cell cytotoxicity (ADCC). Most ADCs such as Kadcyla (T-DM1), Enhertu (T-DXd), Polivy, Padcev, Trodelvy, just to mention a few, rely upon the ADCC-competent IgG1 backbone.^59,61,65,85,88^ The Fc region is also responsible for complement-dependent cytotoxicity and antibody-dependent cellular phagocytosis.

A significant and long-recognized challenge in the field is the heterogeneous distribution of the antibodies when administered systemically.^89^ Antibody internalization and clearance obstruct uptake in solid tumors, limited by tumor vascular permeability and diffusion.^90^ Mathematical analysis of antibody distribution through tumors applying simple scaling relationships can help understanding the tumor physiology and antibody tumor penetration.^90,91^ Fundamental understanding of the mechanisms and time scales of antibody transport and clearance are essential for the prediction of the distance an antibody may permeate through tumor tissue, with the balance between these processes controlling the antibody penetration into a tumor and therefore its optimal efficacy.^91^ Thus, for solid tumors, optimal binding affinity has been suggested to depend on the level of target expression.^91^

Initially, first-generation ADCs used murine antibodies, but they elicited a robust immune response with some patients producing antihuman antibodies, leading to the generation of second-generation chimeric antibodies. These mouse/human chimeric antibodies contain the variable regions of mouse light- and heavy-chains linked to human constant regions. Recent developments in the field have moved toward fully humanized mAbs which do not produce immunogenic responses.^88,92^ Many innovative strategies are being used to maximize the efficacy of ADCs including the use of biparatopic antibodies. These antibodies target two different epitopes of the same target antigen and can help cluster the antigenic receptors, leading to the rapid internalization of ADCs.

For designing present-day ADCs, immunoglobulin G (IgG) is the most widely used antibody isotype. ADCs must have similar pharmacokinetic properties to those of normal human IgGs. Human IgG comprises four subclasses: IgG1, IgG2, IgG3, and IgG4, which differ in their constant and hinge regions. Despite being the most immunogenic, use of IgG3 is avoided in ADC design due to its short serum circulating half-life (∼7 days) when compared to other subclasses (∼21 days).^88,93^ Though IgG1, IgG2, and IgG4 have suitable serum half-lives, IgG2 is rarely used owing to its tendency to dimerize and aggregate *in vivo*.^94^ Most ADCs are developed on IgG1 platforms because of improved solubility, greater complement-fixation, low nonspecific immunity, and better immune effector cell receptor (FcγR)-binding efficiencies of IgG1, which can play a crucial role in anticancer activity of ADCs.^3,84^ Though IgG4 can also induce antibody-dependent phagocytosis (ADCP), its dynamic nature due to Fab-arm exchange contributes to reduced efficacy. Despite this, a few ADCs, such as Gemtuzumab ozogamicin and Inotuzumab ozogamicin, use IgG4 as the platform to target CD33 and CD22, respectively.^95−97^ Gemtuzumab ozogamicin contains an IgG4 core-hinge mutation that blocks Fab-arm exchange.^98^

″Biparatopic” or “bispecific antibodies” are antibodies designed to simultaneously bind to two different epitopes (binding sites) on the same target antigen or two different antigens.^99,100^ This dual binding capacity can offer several advantages in terms of therapeutic applications. Thus, having two antigen-binding sites allows them to target either two distinct epitopes on the same antigen or two different antigens altogether. Their advantages include (i) enhanced targeting–bispecific antibodies can engage two binding sites on the same antigen, potentially increasing binding affinity and specificity; (ii) versatility–they can target multiple antigens simultaneously, which is particularly valuable in cancer therapy or immunological diseases; (iii) cross-linking–in the context of cancer therapy, bispecific antibodies can cross-link cancer cells and immune cells, such as T cells, leading to targeted cell killing.^100^

Antibodies with masked binding domains have been engineered to have one or more of their antigen-binding sites temporarily inactivated or “masked”.^101,102^ These antibodies can be designed to change their binding properties under specific conditions. Their applications include conditional activation, for instance, an antibody may only become active when exposed to specific environmental factors or cellular cues; reduced off-target effects—in some cases, masking can be used to prevent antibody binding to nontarget tissues or cells until it reaches the desired site. CytomX’s Probody technology is one example where the binding domains of antibodies are masked by a peptide, which is selectively cleaved by proteases present at the tumor site.^103^ This allows for the activation of the antibody’s binding and therapeutic functions specifically within the tumor microenvironment.

Both biparatopic/bispecific antibodies and antibodies with masked binding domains are innovative strategies in antibody engineering, offering greater control and versatility in targeting diseases while minimizing off-target effects. These approaches continue to advance in the field of immunotherapy and targeted therapies with potential applications in cancer, autoimmune diseases, and other medical conditions.

The antibodies used in ADC design are large compared to the actual cytotoxic payload, which implies that much of the actual formulation is being utilized to deliver the antigen to the target rather than to execute its pharmacological activity. The large size of the antibody (∼150 kDa) can also cause issues with target penetration in solid tumors. The targeting capacity of antibodies is achieved through its small variable loop structures (V_H_) present at the terminal portion of Fab fragments; therefore, fragments of native antibodies can be used to develop smaller binding motifs such as F(ab)_2_, Fab′, Fab, and Fv fragments. In addition, engineered scaffolds, such as single-chain variable fragments (scFv-Fc), single domain antibodies (sdAbs), and diabodies, are being explored. Furthermore, humanized fragments of unusual IgGs, such as heavy chain variable domain(V_H_H) and heavy chain variable domain-based antibody (V_NAR_) fragments from camelids and shark antibodies, respectively known as nanobodies, are being studied.^104^ Owing to their small size, ease of production, manipulation, conjugation, high solubility, stability, and delayed serum clearance, these antibody fragment conjugates or FDCs have various advantages compared to their antibody precursors.^105^

For example, Fan et al. developed an epidermal growth factor (EGFR)-targeting nanobody-drug conjugate that displays potent anticancer activity in solid tumor models.^104^ In recent years, ASN004, an scFv-Fc ADC, has been developed that targets 5T4 oncofetal antigens expressed on a wide range of malignant tumors. It is designed by conjugating a novel scFv-Fc antibody with an Auristatin F hydroxypropylamide (AF-HPA) payload using Dohtlexin drug-linker technology. The developed ADC has a high drug-antigen ratio (DAR) of approximately 10–15 and has shown tumor repression in preclinical models.^106^ ANT-045 and ANT-043 are other scFv-Fc conjugated ADCs being developed by Antikor Biopharma that have shown successful results against solid tumors. While ANT-043 has tumor ablation effects in gastric, breast, and cancer xenograft models, ANT-045 has high stability, excellent *in vitro* cell-kill potency, and is successful in *in vivo* tumor cures.^107^

With the advancement in molecular biology techniques, site-specific antibody conjugation is being introduced into ADC development to increase the therapeutic efficacy. Antibodies can be engineered using genetic engineering, chemoenzymatic modifications, or metabolic labeling through their Fab or Fc region to introduce specific reaction sites for ease of conjugation. A few modifications include introducing natural amino acids such as cysteine (Cys) or glutamine (Gln).^108−110^ Apart from natural amino acids, unnatural amino acids (UAA) (also referred to as noncanonical amino acids) containing orthogonal side-chain functional groups are also introduced at different positions in the antibody for generating sites for stable conjugation. For example, tubulin inhibitor payload AS269 is conjugated to a HER2- targeted mAb incorporated with a UAA, pAF. The resulting anti-HER2 ADC (ARX788) has a DAR of 1.9 and exhibits a high serum stability and half-life. ARX788 showed strong antitumor activity in mice and is currently undergoing Phase III clinical trials.^111,112^ Recently, other ligands such as short peptide tags, modified glycans, and small molecule-based affinity ligands are also used to generate conjugation sites for antibody payloads.^109,113,114^ Some ADCs in preclinical/clinical development have attenuated effector functional activity, with the intention for reducing off-target off-tumor toxicity.^115−117^

Similar to ADCs, peptide-drug conjugates (PDCs) consist of a peptide two-five amino acids in length bound to the cytotoxic payload via a linker. A key difference between ADCs and PDCs is the substantially smaller size of the peptide component (2–20 kDa) of PDCs than the antibody component (∼150 kDa) of ADCs (5–25 amino acids vs ∼1000 amino acids in peptides and antibodies, respectively)^118,119^ This allows for better absorption and uptake than conventional ADCs, which is especially important for drugs that need to cross the BBB.^118,119^ ADCs and PDCs show a great degree of overlap in terms of linker chemistry and types of cytotoxic payloads. A variety of peptides have been utilized in PDCs, including bicyclic toxin peptides and dendritic peptides.^119^ Bicyclic peptides are small, constrained peptides, many of which have high affinity for target antigens. An example of a PDC is BT8009, which consists of a bicyclic peptide linked to MMAE. BT8009 binds cell adhesion molecule, nectin-4 with high affinity (∼3 nM) and specificity and has shown promise in NSCLC and pancreatic ductal xenograft models.^120^ Currently, several PDCs are in clinical trials or being actively explored.^119^

### 2.2 Linkers for Use in Antibody–Drug Conjugates

Linkers connect the active molecule in an ADC to the antibody that determines the target for the drug. To be effective, a linker should be stable in plasma.^121^ It should not alter the behavior of either the drug or the antibody to which it is attached. The linker should be sufficiently hydrophilic to moderate or mitigate the solubility effects of warheads, which, in most cases, are lipophilic. The linkers should not aggregate; since aggregation is likely to impair the activity of the antibody and may reduce the stability of the ADC. Finally, the linker should release the drug completely and selectively under appropriate conditions. The choice of linker will be determined by the functionality present on the drug and the antibody.

#### 2.2.1 Linker Types

Linkers can be divided into two major classes: cleavable and noncleavable. Cleavable linkers allow the drug to be separated from the antibody without proteolytic cleavage of the antibody, while noncleavable linkers require proteolysis of the antibody for the drug to diffuse to its site of action.

##### 2.2.1.1 Cleavable Linkers

Cleavable linkers allow the drug attached to an antibody to be freed from the ADC without destroying the antibody.^17^ Most commonly, cleavable linkers can be severed by acid, reducing agents, or enzymes.

Acid-sensitive linkers are chosen because the microenvironment of tumors is often acidic and hypoxic;^122^ with parenteral administration ensuring that the drug is released selectively only in the tumor. Acid-cleavable linkers are most commonly hydrazones; for example, a hydrazone is used in the linker for the first USFDA-approved ADC Mylotarg.^123^ One disadvantage of acid-sensitive linkers is premature or nonselective release; Mylotarg was withdrawn in part because of nonselective toxicity (which was mitigated by changes in ADC dosing).^124^

Reductive linkers allow the release of the drug under reducing conditions; the hypoxic environment of tumors makes release of the drug at the tumor site more likely than elsewhere. Reductive linkers have most commonly been disulfides; disulfides can be cleaved either by reduction of the sulfur–sulfur bond or by exchange of thiols (such as glutathione, present in high concentrations in cancer cells) with disulfides to release a thiol (either the antibody or the drug).^121^ Cysteines or other thiols comprise the linker, but when the disulfide is attached to unhindered carbon atoms, nonselective cleavage of the disulfide is more likely. This can be mitigated significantly by two α-methyl groups on one of the carbons at the disulfide.^125,126^

Finally, as the name suggests, enzyme-cleavable linkers incorporate linkages that are selectively cleaved by enzymes. Enzyme-cleavable linkers most commonly use amides as the linking moieties because of the prevalence of enzymes in organisms that process and cleave amide bonds in proteins. Amides are thermally and hydrolytically stable, reducing the likelihood of premature or nonselective drug cleavage. The availability of a wide variety of enzymes and enzyme targets allows linkers to be tuned to avoid hydrolysis by enzymes in normal cells and to facilitate hydrolysis in tumor cells. Initially, di- and tripeptide linkers were used; one of the most common linkers contain the valine-citrulline (Val-Cit) linkage, a target for cathepsin B which is overexpressed in some tumor cells.^127^ Phenylalaninylvaline, valinylalanine, and tetrapeptide linkers have been deployed as well.^128^ The valine-citrulline linker is used in the approved ADC Adcetris, while the terminated clinical candidate T-Rova uses a valine-alanine linker.^129^ While amides are the most common in enzyme-cleavable linkers, other motifs have also been used. For example, substituted ortho-hydroxybenzyl β-glucuronides^17^ and β-galactosides^121^ are susceptible to hydrolysis by β-glucuronidase and β-galactosidase, respectively. Alternatively, substitution of the hydroxybenzyl phosphate-containing linkers yields linkers susceptible to hydrolysis by phosphatases. Cleavage of the acetal or phosphate linkage generates an ortho-hydroxybenzyl group, which subsequently undergoes dealkylative cleavage via an ortho-quinone methide to split the linker. The protected ortho-quinone methide linker is termed a self-immolative linker;^130^ while it is stable under physiological conditions, rapid cleavage occurs when the hydroxy group is unveiled. The self-immolative linker can be altered to incorporate a variety of groups sensitive to various enzymes, allowing linker cleavage to be tailored to the necessary selectivity.

##### 2.2.1.2 Noncleavable Linkers

Noncleavable linkers are designed to remain intact until the antibody is proteolyzed in lysosomes after internalization. One example of a noncleavable linker is the maleimido thioether linker in Kadcyla;^88,131^ while retro-Michael reaction of the β-thioether amide is possible, the modes of cleavage common under physiological conditions are limited. Since conditional cleavage of ADC containing noncleavable linkers is rare to nonexistent, premature release of the drug moiety should be limited to cleavage of the antibody itself, an unlikely event. The off-target toxicity of ADC using noncleavable linkers should thus be minimal. In addition, proteolytic cleavage of the ADC yields a drug moiety substituted with a remaining fragment of the antibody; the peptide fragment is likely polar and charged, preventing the escape of the drug from the cell. The peptide-substituted drug is thus retained within the cell, limiting its ability to kill neighboring cells or to enter circulation and kill nontarget cells; the antibody fragment may also limit transporter-mediated resistance to the drug (though it is unlikely to reduce the effects of other resistance pathways).

**Figure 4:**
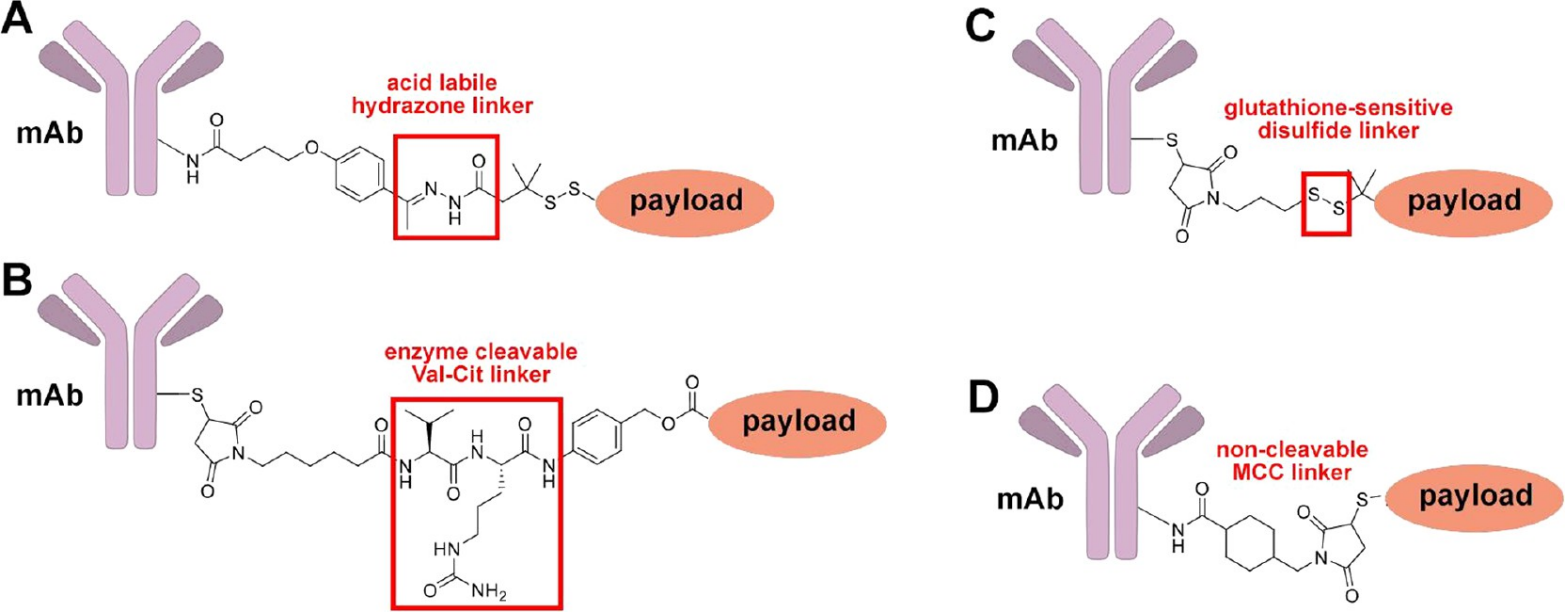
Exemplary ADC linkers: (A) acid labile hydrazone linker; (B) enzyme cleavable Val-Cit linker; (C) glutathione-sensitive disulfide linker; (D) noncleavable maleimidomethyl cyclohexane-1-carboxylate (MCC) linker.

Exemplary cleavable and noncleavable linkers used in ADCs are shown in Figure 4.

##### 2.2.1.3 Branched Linkers

Branched linkers have been devised for ADC to access ADC with higher DAR.^132^ The closest technology to clinical use is that developed by Mersana Therapeutics in which a glycerol-glycolaldehyde condensation polymer (Fleximer) with pendant esters of mercaptocarboxylic acids and drug moieties is prepared and attached to an arylmaleimide-substituted antibody.^133,134^ The method can produce ADC with a DAR of 10–15. A vincristine-functionalized trastuzumab using the technology had antitumor activity similar to and lower toxicity than the corresponding conventionally generated ADC. Two ADC using this technology have entered clinical trials. Upifitomab rilsodotum^135,136^ uses MMAF as the warhead; in a Phase 1/2 trial, it did not show improvement over control against platinum-resistant ovarian cancer. A second ADC, XMT 2056, uses the same platform but uses an STING agonist as the warhead. It is in Phase 1 clinical trials for treating metastatic HER2-positive tumors; unfortunately, its trials are in clinical hold due to a severe adverse event.^137^ Other branched linker methodologies use transglutaminase-mediated coupling of trisubstituted piperidine-containing amines with sequence-modified antibodies,^134,138^ the preparation and use of carbamoylethyl- and carbamoylethoxy arylmaleimides by Firefly Bio as branched linkers for ADC,^139^ the use of pentaerythritol-derived linkers containing amine or oxime moieties for antibody conjugation, fluorescent linkers, and three azide moieties for attachment pf payloads by Sapozhnikova et al.,^140^ and the preparation and use of disubstituted tetrahydropyrazinoindoles as antibody linkers via condensation of aldehyde-containing antibodies with trisubstituted indolemethanehydrazines by R.P. Scherer Technologies.^141^ Linkers that carry two drug units per linker have been also reported for typical hinge cysteine-based conjugation.^142,143^

#### 2.2.2 What Types of Linkers Are Used in ADCs? Why?

Of the currently approved ADCs, cleavable linkers (particularly enzyme-cleavable linkers) predominate, with 11 out of 13 USFDA-approved ADCs having cleavable linkers^19^ (and eight of the ADCs having enzyme-cleavable linkers). Thus, while premature release of the payload (as seen in Mylotarg) in ADC possessing cleavable linkers can lead to unacceptable off-target toxicity and reduced exposure that potentially affects efficacy, the benefits of selective linker cleavage appear to outweigh the liabilities. One way in which this may occur is by the “bystander effect”.^127^ If the payload is released either near a target cell or is transported from (or diffuses from if the drug lacks charged groups such as carboxylates), then the drug can be taken up by neighboring cells, resulting in their death. In most cases, cells in the vicinity of a tumor cell are likely to be tumor cells; thus, the bystander effect allows the ADC to affect cells in the vicinity of an antigen-presenting cell. Since one of the many ways that cancer cells evade treatment is to downregulate the production and expression of surface antigens, ADCs using cleavable linkers can take advantage of the bystander effect and circumvent some of the resistance modes of tumor cells.

### 2.3 Drugs Incorporated into ADCs

#### 2.3.1 What Are the Requirements for Drugs To Be Incorporated as Payloads into ADCs?

First, antibodies such as IgG (approximately 150 kDa) are large; attachment of many drug molecules to an antibody often makes it more lipophilic, causing aggregation of the ADC and subsequent degradation and inactivation. The number of drug molecules linked to an antibody is small (in most cases, four or fewer), making the effective dose of the drug in an ADC small. In addition, the low numbers of antigens per cell and the imperfect delivery of ADC to cells further reduces the likely dose of drug from ADC.^131^ Thus, because only a small dose of drug is possible, the drug chosen for use in an ADC needs to be highly potent (exhibiting its effects at nM to pM concentrations) to boost efficacy. However, the effective dosage of drug to cells from ADCs has shown a plateau in mice, indicating that a sufficient ADC can be delivered to a tumor to oversaturate it with the drug (with the caveat that the drugs delivered in this case were highly potent and adapted for the ADC).^144^ Second, drugs in ADCs are attached to antibodies and are not free to diffuse into cells. If the linker between a drug and an antibody remains intact until delivery, the antibody controls where the drug is released, allowing less selective drugs to be used in ADCs than those given systemically. Finally, the drug needs to be stable both to storage and to administration; since ADCs are administered parentally, the drug in an ADC needs to be stable in blood and plasma (though attachment to an antibody may provide steric shielding for a drug and thus may reduce its reactivity and increase its stability). A secondary consideration is if the ADC is being used in combination with other drugs, the ADC warhead should act on a different target, a different biological pathway, or at a different phase of the cell cycle than the other drugs being used.^3^

#### 2.3.2 What Types of Drugs Are Used as Payloads for ADCs?

- Auristatins are tubulin polymerization inhibitors derived from the marine natural product dolastatin 10, with potencies of 50–100 pM.^88^ Monomethyl auristatins F and E have been used as warheads for ADCs such as Adcetris, Polivy, and Blenrep.^145^
- Maytansinoids are ansa-macrolide natural products isolated from the shrub *Maytenus serrata*.^146^ They bind tubulin and prevent its assembly into microtubules, thus inhibiting mitosis and cell replication.^147^ Maytansinoids were tried as antitumor agents but were not effective at tolerable concentrations. The maytansine DM1 is the warhead of the ADC Kadcyla.^88^
- Camptothecin and its analogs such as irinotecan, topotecan, govitecan, SN-38, exatecan, and deruxtecan inhibit topoisomerase 1, an enzyme that unwinds and cuts a single strand of supercoiled DNA, allowing it to be repaired and replicated;^148^ its inhibition leads to DNA cleavage and cell death. A variety of camptothecin analogs have been tried as antitumor agents, but their aqueous solubilities and side effects have hindered their use as antitumor agents.^149^ Some of the camptothecins have also been susceptible to export by ABC transporters; annulation of an additional ring as in exatecan prevented transport but caused myelotoxicity, which was reduced further by addition of a maleimide-terminated peptide to form deruxtecan. Camptothecin analogs are warheads in the USFDA-approved ADC Enhertu and Trodelvy.^88^
- Pyrrolobenzodiazepines such as tesirine and talirine were derived from the natural product anthramycin.^150^ They alkylate DNA with extremely high potencies (as low as 100 fM).^88^ One pyrrolobenzodiazepine (SJG-136) was tried as an antitumor agent but progressed only to Phase I trials due to significant toxicity with no antitumor response.^151^ Their high potency makes them attractive warheads for ADCs.^152^ A variety of ADCs with pyrrolobenzodiazepine warheads have been tried, with one (Zylonta) having been approved as of December 2021 for the treatment of B-cell lymphomas. Their dimeric nature and the presence of two alkylating moieties allows them to cross-link DNA, which creates DNA damage that is difficult to repair. However, these same features are also likely responsible for undesired off-target toxicity. Structurally related indolinobenzodiazepine dimers have also been studied as warheads for ADC;^153^ incorporation of monoamine indolinobenzodiazepines has been used to generate antitumor ADC with reduced off-target toxicities.^131,154^
- Calicheamicin γ^1^ and related natural products such as dynemicin contain strained enediyne moieties. Under reductive conditions, DNA-bound calicheamicin undergoes Bergmann cyclization to generate diradicals which cleave both strands of DNA, leading to damage which is difficult or impossible for cells to repair. It inhibits DNA replication at pg/mL concentrations^155^ but is also toxic as a result. The combination of potency and toxicity suggested the potential use of calicheamicin γ^1^ in ADCs. The first approved ADC, Mylotarg, incorporated calicheamicin as a warhead; it was approved in 2001 but withdrawn in 2010 because of its toxicity. Development of modified dosing allowed Mylotarg to be reapproved in 2017 with an expanded patient population.^124^ The ADC Besponsa also uses a calicheamicin derivative as a warhead.
- Many other compounds have been tried as warheads for ADC. Duocarmycins such as CC-1065 and seco-DUBA are DNA-alkylating agents effective at nanomolar to picomolar concentrations.^156^ Tubulysins are noncanonical peptides containing thiazole moieties that act as microtubule polymerization inhibitors and are active against cancer cells at nanomolar to picomolar concentrations,^157^ making them attractive candidates for ADCs.^158^ However, some of the tubulysins are unstable in aqueous environment and can show unselective toxicity in cancer cells. Cryptophycins are macrolides which inhibit tubulin polymerization at picomolar concentrations; however, trials against cancer showed toxicity but not efficacy.^159,160^ Despite this, their potency has made them attractive payloads for ADCs.^161^ The spliceostatins and thailanostatins are natural products that inhibit the spliceosome, modifying mRNA sequences and thus influencing protein expression. They have shown inhibition of cancer cells at nanomolar concentrations^162^ and hence are potential ADC payloads.^163^ Doxorubicin is an intercalating agent for modification of DNA which was used as one of the first payloads for ADCs but was not effective because of its low potency. However, if the DAR of doxorubicin conjugates can be increased by newer conjugation methods, it may prove to be an effective warhead for ADCs. Alternatively, more potent anthracycline drugs have been developed. For example, PNU-159682 is an anthracycline acting by a similar mechanism to doxorubicin which was found to be nearly 1000 times more potent than doxorubicin.^164,165^ This enhanced potency enables ADCs incorporating it to be highly effective.^166^ α-Amanitin, a fungal toxin which inhibits RNA polymerase II, is a significant cause of liver failure and death from toxic mushroom ingestion.^167^ Its toxicity, robustness, and moderate size make it a reasonable choice as a warhead for ADC.^168^ Protein toxins have been used as warheads for ADCs; for example, Pseudomonas exotoxin A^169^ is the warhead for the USFDA-approved ADC Lumoxiti.^170^ Diphtheria exotoxin^171^ has also been used as a payload for ADC.^172^
- Finally, immunomodulating agents have been tested as antibody payloads, either to suppress immune responses for anti-inflammatory or immunosuppressant activities or to enhance the immune response to cancer.^173−177^ In addition, an ADC with antibiotic warheads have been designed for use as an antibacterial agent.^178^

### 2.4 Conjugation Methods for Antibody–Drug Conjugates

#### 2.4.1 What Are the Important Features of a Conjugation Method?

As with the linking moiety, it is important that the method of attaching a drug to an antibody through a linker does not alter the activity or stability of the drug or the antibody. In addition, conjugation should be efficient, proceed in high yield (so that as little as possible of the reagents are used per unit of ADC) and proceed as rapidly as possible. It should also be selective and predictable so that the locations of attachment of a drug to the antibody are controllable, known, and consistent.^179^ While it would be optimal to have a single species generated by a method, it is not necessary as long as the ADC is composed of a consistent mixture of species with consistent stability, biological and physical properties, and biological activity.

#### 2.4.2 What Is the Structure of an Antibody? Where Can It Be Functionalized?

Most antibodies used for ADC are immunoglobulins, of which the most commonly used is immunoglobulin G (IgG) (Figure 1).^127^ IgG contains two heavy chains and two light chains. The heavy chains are attached to each other and to the light chains through four (interchain) disulfide S–S bonds that hold the antibody together. Twelve other intrachain disulfide bonds control the tertiary structures of the light and heavy chains. There are also roughly 80 lysine residues in a typical antibody, of which 40 may be functionalized. In addition, one of the heavy chain glutamine residues is substituted with a branched-chain heptasaccharide which improves the ability of the antibody to trigger an immune response.^180^

#### 2.4.3 Conjugation Methods to Native Antibodies

##### 2.4.3.1 Lysine

Under most circumstances, however, the reactivity of specific residues is rarely controlled, unless the basicity of a residue is significantly altered by its position in the protein sequence or by the side chains of nearby residues. In most cases, the average number of residues functionalized can be controlled by stoichiometry but not the position of functionalization. The lysine ε-amine forms stable amides with acylating agents such as *N*-hydroxysuccinimidyl esters (particularly sulfonated *N*-hydroxysuccinimidyl esters,^181^ which have better aqueous solubilities), benzoyl fluorides,^182^ and acid anhydrides.^183^ Acylating agents can form esters with tyrosine, threonine, or serine residues, but the esters are not stable; thus, if not prevented, some of the acylating agents will be consumed, decreasing the amount of drug attached to the antibody. Affinity peptides have been used to control the location of conjugation with antibodies;^184^ for example, binding of a peptide-containing acylating agent to the Fc domain of an antibody directs the acylation reaction to nearby residues^185^ and the conjugation method has been performed on gram scale using a peptide-substituted thioester.^186^ Cleavage of the peptide yielded a thiol available for further reaction.

##### 2.4.3.2 Cysteine

Cysteine is the most common residue functionalized in antibodies because the sulfur atom of its side chain is highly nucleophilic. Cysteine’s thiol moiety is acidic enough for its anion to be accessible under physiological conditions; the resultant anion is more reactive toward electrophiles than other residues but is not basic enough to cause side reactions. IgG does not natively contain free cysteine residues, requiring some of the disulfide bonds of the antibody to be cleaved in order to allow functionalization. The four interchain disulfide bonds are most often used;^187^ while this preserves the structure and function of the individual antibody fragments, the stability of the antibody may be compromised. Selectivity among the cysteine residues is difficult; while a single species can be formed if all eight cysteines are functionalized (as for the approved ADC Troldelvy and Enhertu), most methods yield mixtures of products.

The most common monofunctionalization reagent used is the maleimide. Maleimides undergo Michael additions of thiols readily under ambient conditions to yield alkylthiosuccinimides.^188^ The thiol-succinimide adducts, however, can undergo retro-Michael addition, which could lead to either incomplete functionalization of the antibody or premature release of the drug from the antibody and undesired toxicity. Ring opening of the imide to form a carboxylate-containing amide reduces retro-Michael reactions substantially.^189^ Exposure of the imide product to mildly basic conditions can be used to form the carboxylate;^190^ the presence of nearby positively charged residues facilitates imide ring opening and stabilizes the maleimide adducts. Intramolecular reactions can also be used to stabilize the cysteine adducts.^191^ Other monofunctionalization reagents for cysteine include palladium aryl complexes to yield aryl thioethers,^192^ disulfides derived from exogenous thiols (particularly acidic thiols),^193^ and alkenyl and alkynyl phosphorus^194,195^ and iodine reagents.^196^ The known reactions of haloacetamides with cysteine residues can also be used;^96^ while stable thioether linkages are formed, their reactivity may lead to difficulty in controlling the number of appended drug moieties or their residue selectivities.

Disulfide rebridging can be used to reduce the potential instability caused by complete functionalization of the cysteines generated by reduction of the four interchain disulfide bonds.^179^ Reaction of the intermediate octathiols with biselectrophiles yields bisthioethers in which two of the cysteines are connected by alkyl or aryl groups; the thioether linkages are robust and can stabilize the dimeric antibody structure. The number of bridges formed (and thus the number of drugs incorporated) is easily controlled; the structure of the alkylating agent and the number of available sites for drug conjugation on it determine the carrying capacity of the ADC. One disadvantage of the method is that the biselectrophiles are likely to not be commercially available and thus require synthesis. A variety of reagents has been developed for disulfide rebridging. Abzena developed bis(sulfonylmethyl)methyl ketones (ThioBridge) which undergo sequential elimination and Michael reactions to yield thioether-substituted ketones.^197^ The payload can then be attached with alkoxyamines or by reductive amination. A similar method for rebridging has been used by Novartis with dichloroacetone as the biselectrophile.^198^ Maleimides with bromo-, iodo-, or arylthiol-substituents undergo Michael/retro-Michael reactions to yield substituted maleimides;^199−201^ incubation at pH 8.4 yields maleimidic acids which are resistant to Michael and retro-Michael reactions at the linker. Dihalo- or bis(phenylthio)pyridazines undergo analogous reactions to dihalo- or bis(arylthiol)maleimides but are resistant to linker cleavage by retro-Michael reactions.^202^ The pyridazines can incorporate multiple drug moieties; in addition, their reactivity can be controlled by temperature. Other biselectrophiles used for disulfide rebridging are aryl di(bromomethyl)quinoxalines (C-Lock, Concortis Therapeutics^203^), cis-platinum diamine complexes (Invictus Oncology),^204^ and divinylpyrimidines.^205^

##### 2.4.3.3 Other Residues

The N-terminal amino group of peptides is less basic than the ε-amino groups of lysine residues, making selective reactions at the N-terminus of the antibody heavy chains possible. The proximity of the N-termini to the receptor binding site of the antibody may complicate functionalization. While reaction at the C-terminus would be preferable (because the C-terminus of the heavy chains is distant from the antigen- or receptor-binding sites of the antibody), chemical methods for doing so are uncommon. Transamination at an N-terminal glutamine residue with a formylpyridinium salt followed by reaction with an alkoxyamine yielded a stable N-terminal oxime ether.^206^ Oxidation of an N-terminal serine moiety yields a formylglycine residue, which can react with an alkoxyamine to form an oxime ether or can form nitrogen heterocycles by condensation with carbonyl compounds.

Arginine residues are unreactive under physiological conditions because the guanidine conjugate base of the native guanidinium ion is highly basic, inhibiting reaction. However, dicarbonyl compounds can react with amidines or guanidines to yield stable imidazoles. An azidophenylglyoxal was designed for reaction with arginine residues to form azidophenylaminoimidazole moieties; azide–alkyne cycloaddition with a terminal alkyne-substituted warhead yields the functionalized antibody.^207^ Finally, Ugi reactions of a lysine residue, a nearby aspartate or glutamate residue, an azidoaldehyde, and an isonitrile formed azide-functionalized macrocycles amenable to drug attachment via azide–alkyne cycloaddition.^208^

#### 2.4.4 Conjugation Methods for Non-Native Antibodies

If an ADC with a precisely known structure is desired, native antibodies may not allow sufficient selectivity. Incorporating non-native functional groups by modification of the heavy chain sequence makes selective antibody conjugation with existing chemistry possible. Antibodies can be produced either by inoculation of mice with an antigen, by phage display, or by biopanning,^209^ separation of the cells producing the antibody and hybridization with cancer cells, cloning of the antibody sequence, and insertion into CHO cells.^210^ Since the antibody DNA and protein sequences are known, they can be modified to obtain antibodies with non-native sequences.^210^ Multiple methods of sequence modification may be useful.

##### 2.4.4.1 Cysteine Incorporation

IgG normally does not possess free cysteine residues; the cysteines are connected by oxidation to intra- and interchain disulfides. Because the reactivity of cysteine is distinct from other residues, a non-native cysteine residue should be readily functionalizable, and has been used in technologies such as THIOMAB.^211^ Incorporation of an N-terminal cysteine allows reaction with aldehydes to form thiazolidines^212^ which slowly releases the aldehyde payload and enables the adducts to be effective as ADC. The position of insertion of the cysteine into the antibody sequence, however, needs to be chosen to minimize perturbation of antibody structure; in addition, as noted earlier, the presence of positively charged residues nearby facilitates ring opening of maleimide conjugates and improves their stabilities to deconjugation. Another caveat of cysteine incorporation is that cysteines form disulfides with glutathione during antibody production which must be reductively cleaved, but methods have been developed to address this issue.^213^ Recent studies have shown that antibody engineering methods are used to add two or three unpaired Cys residues, boosting the DAR of ADCs to >2 per antibody.^109^

##### 2.4.4.2 Noncanonical Amino Acids

The use of amino acids containing functionality not normally present in peptides or antibodies allows facile, biocompatible, and selective reactions such as azide–alkyne cycloaddition and oxime ether formation to be used for linking to drugs. However, the biosynthesis of antibodies containing noncanonical amino acids (NCAA) is difficult. NCAA incorporation normally requires one of the stop codons in translation (most commonly, the amber codon UAG) to be suppressed and instead be used to encode the desired amino acid. Amber codon suppression, however, is believed to harm the cells in which it is deployed,^214^ requiring alterations to the expression methods. Cell-free systems can be used to generate NCAA-containing antibodies, but they lack the ability to glycosylate the finished antibody,^215^ which reduces its ability to summon an immune response. In addition, the site of incorporation requires optimization to avoid altering the stability or function of the antibody. Antigen- and receptor-binding sites and the hinge regions must be avoided, while solvent-exposed sites are preferred to increase the rate of drug attachment.

p-Acetylphenylalanine,^216,217^ p-azidophenylalanine,^218,219^ p-azidomethylphenylalanine,^220,221^ and azidolysine^222−224^ are stable in cells and do not alter protein structure significantly. p-Acetylphenylalanine reacts readily with alkoxyamines, while p-azidophenylalanine, p-azidomethylphenylalanine, and azidolysine undergo either copper-catalyzed or strain-promoted azide–alkyne cycloadditions with terminal alkynes or cyclic alkynes, respectively. All can be incorporated into peptides in reasonable yields. N-Propargyllysine is stable and amenable to copper-catalyzed azide–alkyne cycloaddition^225^ but differs enough in structure from lysine to inhibit its incorporation into peptides.^226^ Cyclopropene- and cyclopentadiene-substituted amino acids undergo cycloaddition reactions with tetrazines or maleimides to form stable pyrazine or bicycloheptene adducts.^227,228^ Selenocysteine is a rare but natural amino acid; the selenol group is more acidic and yields an even better nucleophile than a cysteine residue. Incorporation of selenocysteine into an antibody was achieved,^229^ despite low yields because of undesired termination at the erstwhile stop codon. Finally, suppression of two of the three stop codons allows peptides to be translated with two NCAA, which allows two different warheads to be attached to a single ADC.^230^ The difficulty of incorporating NCAA into an antibody likely means that the use of NCAA-based conjugation reduces the possible drug capacity of the ADC.

##### 2.4.4.3 Incorporation of Additional Amino Acids

Addition of amino acid sequences to the C-terminus of the heavy chain of an antibody can be used to selectively conjugate drugs. (While the addition of amino acids increases the size of the ADC, large addends are required to perturb the antibody’s movement because it is already large, 150 kDa in the case of IgG). The cysteine in a π-clamp sequence (FCPF) selectively reacts with perfluorinated benzenes to yield substituted fluoroaryl thioethers.^231^ The cysteine in a different cysteine-containing sequence (LCYPWVY) undergoes reaction with dibenzocyclooctynes to yield stable dibenzocyclooctenyl thioethers.^232^ Both reactions can thus be used to attach drugs to an antibody. Appending a receptor to the C-terminus of an antibody can be used to attach drugs to the antibody if a covalent or irreversible inhibitor of the receptor exists; this method was used with CD38.^233^ Finally, a catalytic antibody sequence (38C2) was incorporated into an IgG1 antibody (without significant alteration of its properties); the antibody alters the basicity of nearby lysine and arginine residues, making their selective functionalization possible.^207^ The size and position of added sequences are likely to limit the drug loading of an antibody.

#### 2.4.5 Enzymatic Methods for Conjugation

Enzymatic methods can circumvent some of the limitations that exist in chemical methods to conjugate drugs to antibodies. The evolutionary constraints for enzyme function are well-suited for chemoselective attachment of substrates to antibodies, and the development of bioorthogonal chemistries to interrogate protein function has further enhanced their capabilities.

Some enzymic modifications allow moieties to be directly attached to an antibody; while they require specific chemical matter to be present or may require mutant or additional sequences to be added to an antibody, the methods need only one step to functionalize antibodies. In most cases, the need for a single residue or functional handle for specificity limits the number of warheads that can be easily attached to an antibody. Transglutaminases exchange the amino group of a glutamine’s amide moiety for another amine, allowing for facile attachment of amino-containing payloads or linkers. A glutamine residue (Q295) in the heavy chain possesses a carbohydrate moiety that helps to determine the physical properties and immune responses of the antibody; removal of the carbohydrate can have negative consequences for ADC performance^234^ but provides a reliable attachment point for payloads.^235^ Alternatively, inclusion of glutamine residues (either by extension or by mutation of the antibody sequence) allows transglutaminase-mediated coupling reactions to attach payloads without perturbing the immune effects of the antibody.^236^ A large variety of amine-containing linkers can be used; with mutant transglutaminases, hydrazones (acyl hydrazines) can also be exchanged with glutamine residues.^237^ Prenyltransferases attach farnesyl or geranylgeranyl (15- or twenty-carbon) pyrophosphates to cysteine residues followed by two aliphatic amino acid residues.^238^ One of the prenyl methyl groups can be substituted; when a ketone or azide moiety is included, bioorthogonal coupling methods are applicable to attachment of linkers or payloads.^239^ Sortases attach N-terminal substituents to peptides or antibodies with the C-terminal sequence LPXTG–OH (effectively coupling an amine to the C-terminal glycine), allowing attachment of N-terminal substituents to an antibody where they are less likely to interfere with other functions.^240^ Butelase 1 appends dipeptides to C-terminal asparaginylhistidylvaline moieties to form dipeptidyl asparagine amides; the enzyme has been used with sortase A and modification of the antibody light chains to provide doubly modified antibodies.^241^ SpyLigase attaches a peptide containing an N-terminal tag to a peptide with a corresponding C-terminal tag, eliding the intervening peptide and forming a new substituted antibody.^242^ Phosphopanteinyltransferases acylate serine residues with CoA thioesters;^243^ while the functionality that can be incorporated is broader (requiring only a CoA thioester, the ester linkage formed may not be sufficiently stable or persistent.

Other enzymic methods convert native peptides or amino acid residues to reactive moieties that are amenable to conjugation with a variety of linkers or warheads but do not directly attach substituents to antibodies. Formylglycine-generating enzyme (FGE) reacts with peptides with the N-terminal sequence H-CXPXR (X = any amino acid), again requiring mutation of the antibody sequence. FGE generates formylglycine residues from the N-terminal cysteine;^244^ the aldehyde moiety is amenable to condensation with oxime ethers or with electron-rich arylmethylhydrazines in iso-Pictet-Spengler reactions to form aryl-fused tetrahydropyridazines.^245^ FGE-mediated conjugation may be useful when conjugation with cysteines is not compatible with the linker chemistry or when different requirements for linker stability are necessary. Tyrosinases and horseradish peroxidases oxidize tyrosine residues to form ortho-quinones which can undergo either Michael addition of amines to the quinones and rearomatization to yield stable 3-aminotyrosine residues^246^ or Diels–Alder reactions.^247^ While aminotyrosine residues are potentially oxidizable, the carbon–nitrogen bond formed is robust.

Finally, the sugar moieties present in the antibodies can be remodeled to yield attachment points for conjugation. Fucose, sialic acid, and galactose moieties contain vicinal *cis*-hydroxyl groups which can be oxidatively cleaved by sodium periodate to yield dialdehydes; condensation with oxime ethers or hydrazines yields oxime ethers and hydrazones.^248^ While the oxidation provides multiple attachment points for payloads, it also can oxidize methionine residues of the antibody, which increases clearance and decreases efficacy.^249^ An alternative method is to alter the sugar moieties by incorporating sugars with non-native functionalities into the pendant sugar moieties. For example, endoglycosidases catalyze the exchange of the terminal aminosugar moieties of saccharides, incorporating 2-N-acetylglycosamines into saccharides via oxazoline intermediates.^250^ Glucosyl-, galactosyl-, and thiofucosylamines with azide or ketone substituents (or, with thiofucosylamine, a thiol group) can thus be swapped into the sugar moieties of antibodies;^251^ reaction of azides with alkynes (using copper catalysis or strained alkynes), of ketones with alkoxyamines, or of thiofucose moieties with maleimides immobilizes payloads onto antibodies. Both methods may alter the immune functions of the antibody–drug conjugate by sugar modification and thus the activity of the conjugate.

#### 2.4.6 Drug–Antibody Ratio

Drug–antibody ratio (DAR) characterizes how many drug molecules an ADC can carry; theoretically, it should characterize the ability of an ADC to deliver drug to a tumor and thus positively correlate to effectiveness.^19^ However, the presence of large numbers of drugs (and thus large numbers of linkers) on an ADC perturbs the properties of the antibody. Lipophilic drugs and linkers increase the aggregation of ADC, preventing them from reaching their site of action; they may also hinder access to binding sites necessary for antigen recognition or binding, reducing the activity of the ADC. Reducing the lipophilicity of linker moieties has been used to reduce the negative consequences of antibody functionalization,^121^ but not the alteration in ADC properties. In addition, significant toxicity has been noted for ADC with high DAR (DAR ≥ 8) and high DAR may increase the clearance of ADC (reducing their residence time and effectiveness).^252^ DAR is an important analytical property of ADC; the ability to produce ADC with consistent DAR is likely to lead to ADC with consistent properties and biological activity and thus is likely a critical attribute for ADC synthesis and production.

#### 2.4.7 What Types of Drugs, Linkers, and Conjugation Methods Have Been Used for Approved ADCs?

A variety of types of drugs are used in clinically approved ADCs and in ADCs in clinical trials (discussed further in the text). Calicheamicin, maytansinoids, auristatins, duocarmycins, and pyrrolobenzodiazepines have been used, while tubulysin-, eribulin-, and amberstatin-containing ADCs are being researched in clinical trials.^19^ The drugs in ADCs are highly potent (effective at nM to pM concentrations); the difficulty in delivering significant amounts of drug to tumors with an ADC (as noted earlier) may explain the prevalence of potent drugs in ADC.

Most of the currently approved drugs and nearly all ADCs in clinical trials use cleavable linkers, and in most cases, enzyme-cleavable linkers.^19^ The preference for cleavable linkers indicates (as noted) that the contribution of bystander effects to the ADC clinical effectiveness is critical. In addition, the continued development of enzyme-cleavable linkers is likely important. While the toxicity of Mylotarg and its subsequent withdrawal and reapproval provided concern for chemically reactive linkers, the incorporation of chemically stable linkers allows the best of both worlds. The controlled drug release provides safety assurance as well as increased antitumor response via the bystander effect. The use of cleavable linkers also may prevent or reduce the resistance of tumors to ADCs by decreasing the effect of antigen loss on toxicity and releasing ADCs from the requirement for lysosomal cleavage.

As of mid-2023, all of the approved ADCs used conjugation to lysine or cysteine residues; none of the currently approved ADC used site-selective functionalization methods,^19^ while few of the ADCs use site-selective conjugation methods. It is unclear why site-selective methods have not been more effective; knowledge of antibody structure and function should be sufficient to avoid antibody inactivation or a loss of function. Methods to site-specifically conjugate drugs to antibodies were less advanced and may have been insufficient for incorporation into a drug (or may have had insufficient data and thus too much risk to incorporate into a clinical candidate). The reasons for the failures in the current trials, however, appear unclear.

ADCs currently approved and in clinical trials vary significantly in their DAR as well; DAR between 1.8 and 8 have been tried (see Section 7.3 below enlisting approved ADCs), with most possessing DAR around 4. The DAR may be an artifact of the conjugation method; immobilization using the interchain bridging disulfides should yield ADC with a DAR of roughly 4. The presence of larger linker and drug moieties may also require a lower DAR to avoid aggregation or inactivation of the ADC. ADCs with higher DARs and less-potent drugs have not yet reached clinical trials; it is not clear whether this is due to limitations on DAR, ineffectiveness in previously tried high-DAR ADCs with less potent payloads, or some other reason.

### 2.5 Selection/Optimization of Target Antigen Moiety

The efficacy of an ADC depends on the expression levels of target antigens. ADCs are designed to release the payload upon interaction with cognate antigens.^88,253^ As the field continues to grow, over 50 antigens have been identified as successful ADC targets under various clinical evaluation stages.^253,254^ To achieve optimal therapeutic efficiency, antibody–antigen binding affinity can be optimized on a case-by-case basis depending on the tumor size, target antigen concentration, and receptor-mediated internalization kinetics.

The most used antigenic targets are CD19, erb-b2 receptor tyrosine kinase 2 (ERBB2), HER2, CD22, CD30, CD33, CD79b, and Mesothelin (MSLN).^46^ These antigenic markers vary depending on the tumor type. Antigens such as HER2, EGFR, 5T4, trophoblast cell-surface antigen 2 (TROP2), and nectin 4 are commonly used ADC targets in solid tumors due to their higher level of expression in malignant cells when compared to the nonmalignant ones.^46,255,256^ For hematological cancers, markers such as CD30, CD22, CD79b, CD19, CD138, and B-cell maturation antigen (BCMA), which are distinct from solid tumor markers, are commonly used.^88^ For example, CD30, the target of brentuximab vedotin (Adcetris by Seattle Genetics), is mainly expressed by the malignant lymphoid cells of Hodgkin lymphoma and anaplastic large cell lymphoma (ALCL). Likewise, for specifically targeting B cell lineages, markers such as CD22, CD79b, and BCMA are used. These markers have successfully been used as the targets of inotuzumab ozogamicin (for the treatment of relapsed or refractory (R/R) B cell acute lymphoblastic leukemia), polatuzumab vedotin (for R/R diffuse large B cell lymphoma), and belantamab mafodotin (for R/R multiple myeloma), respectively.^257,258^ Apart from these well-known targets, work to identify suitable antigenic targets in the tumor microenvironment, such as in the stroma and vasculature, is ongoing.

A successful antigenic target of an ADC should be uniformly and heterogeneously expressed on the surface of target cells or other components of the tumor microenvironment and have minimal to no expression in off-target sites.^259,260^ Other essential factors include the internalization and processing of ADCs that help in their cellular uptake and increase the efficiency of the cytotoxic drug. In addition, it is advantageous that ADCs are designed against functional/oncogenic targets as they can have higher antitumor activity; for example, data from preclinical studies suggest that HER2-targeted ADCs T-DM1 and T-DXd having anti-HER2 mAb trastuzumab’s Fab region prevents ligand-independent HER2 dimerization, inhibiting HER2 downstream signaling.

Once the antibody in an ADC binds to the target antigen, it is internalized via the early endosome, and the internalization rate and efficiency depend on the target antigen and the payload. Affinity correlates well with internalization with higher affinity often resulting in rapid internalization up to a ceiling limit.^88^ However, a very strong binding affinity between the antibody and target antigen can lead to uneven distribution of ADCs in solid tumors due to the presence “binding site barrier”. This leads to stronger binding of antibodies with the antigens presents on cells near blood vessels and less penetration away from the tumors.^261,262^

Once ADCs are inside the cell, the endosomes containing them mature into late endosomes and finally fuse with lysosomes. This is followed by the release of cytotoxic payloads in the lysosome upon linker cleavage or antibody degradation. The payload eventually escapes into the cytosol to exert its effects. ADCs with cleavable linkers can release drug to neighboring cells both with and without ADC internalization.^263,264^ ADC with noncleavable linkers require proteolysis of the ADC for drug release. Proteolysis yields drugs with an attached amino acid residue (lysine or cysteine) which is charged at cellular pH.^265^ The ability of drugs to diffuse out of the cell depends on their lipophilicity; charge impairs their ability to diffuse across the membrane and thus leave the cell through passive transport. ABC transporters prefer neutral and hydrophobic compounds and export neutral hydrophobic drugs out of the cell, so drugs with amino acid residues derived from ADC catabolism are poor substrates for ABC^266^ and require help to leave the lysosome^267^ and the cell. The susceptibility of payloads to active transport decreases the effectiveness of ADC but also makes cells that do not take up the antibody subject to its effects. In particular, the bystander effect depends on how much drug can escape a cell and then accumulate in neighboring cells and if it is sufficient to kill them.^265^

ADCs are used to treat solid tumors, but a heterogeneous expression of antigenic targets in these tumors may be overcome by the ’bystander-killing effect’,^84,268,269^ where the payload is transferred from the antigen-positive cells to the antigen-negative cells in the tumor microenvironment. The lipophilic payload for internalized ADCs diffuses across cell membranes and significantly contributes to ADC activity against tumors. For some ADCs, the payload release might happen extracellularly, killing antigen-negative cells located in proximity.^259^

Table 1 summarizes antigens used as targets of ADCs in development and in the clinic.^4,254,270,271^

**Table 1:** Target Antigens for ADCs in Development and in the Clinic

Disease	Target antigens
Breast cancer	CD25, CD174, CD197, CD205, CD228, c-MET, CRIPTO, HER2, HER3, FLOR1, Globo H, GPNMB, IGF-1R, integrin β-6, PTK7, nectin-4, ROR2, SLC39A6
Ovarian cancer	CA125, CD142, CD205, FLOR1, Globo H, mesothelin, PTK7, TIM-1
Prostate cancer	CD46, PSMA, STEAP-1, SLC44A4, TENB2
Lung cancer	CD25, CD56, CD71, CD228, CD326, CRIPTO, EGFR, HER3, FAP, Globo H, GD2, IGF-1R, integrin β-6, mesothelin, PTK7, ROR2, SLC34A2, SLC39A6, Axl, αv β6
Pancreatic cancer	CD25, CD71, CD74, CD227, CD228, GRP20, GCC, IGF-1R, integrin β-6, nectin-4, SLC34A2, SLC44A4, αv β6, mesothelin
Melanoma	CD276, GD2, GPNMB, ED-B, PMEL 17, endothelin B receptor
Gastric cancer	CD25, CD197 (CCR7), CD228 (P79, SEMF), FLOR1(FRα), Globo H, GRP20, GCC, SLC39A6 (LIV1A ZIP6)
Colorectal cancer	CD74, CD174, CD166, CD227, CD326, CEACAM5, CRIPTO, FAP, ED-B, HER3
Bladder cancer	CD25, CD205(Ly75)
Liver cancer	CD276 (B7–H3), c-MET
Renal cancer	AGS-16, EGFR, c-MET, CAIX, CD70, FLOR1, TIM-1
Multiple Myeloma	CD38, CD46, CD56, CD74, CD138, CD269, endothelin B receptor
Head and neck cancer	CD71 (transferrin R), CD197 (CCR7), EGFR, SLC39A6 (LIV1A ZIP6)
Non-Hodgkin lymphoma	CD19, CD20, CD21, CD22, CD25, CD30, CD37, CD70, CD71, CD72, CD79a/b, CD180, CD205, ROR1
Hodgkin’s lymphoma	CD25, CD30, CD197
Acute myeloid leukemia	CD25, CD33, CD123, FLT3
Gliomas	CD25, EGFR
Mesothelioma	mesothelin, CD228

## 3 Landscape View of Antibody–Drug Conjugate Research–insights from the CAS Content Collection

The CAS Content Collection^81^ is the largest human-compiled collection of published scientific information, which represents a valuable resource to access and keep up to date on the scientific literature all over the world across disciplines including chemistry, biomedical sciences, engineering, materials science, agricultural science, and many more, thus allowing quantitative analysis of global research publications across various parameters including time, geography, scientific area, medical application, disease, and chemical composition. Currently, there are over 25,000 scientific publications (mainly journal articles and patents) in the CAS Content Collection related to ADC research and development. There has been a steady growth of these documents over the last three decades, with an >30% increase in the last three years (Figure 3). Noteworthy, while in the earlier years scientific journal publications dominated, after around the year 2000 the number of patents clearly outnumber them, correlating well with the initial accumulation of scientific knowledge and its subsequent transfer into patentable applications.

**Figure 5:**
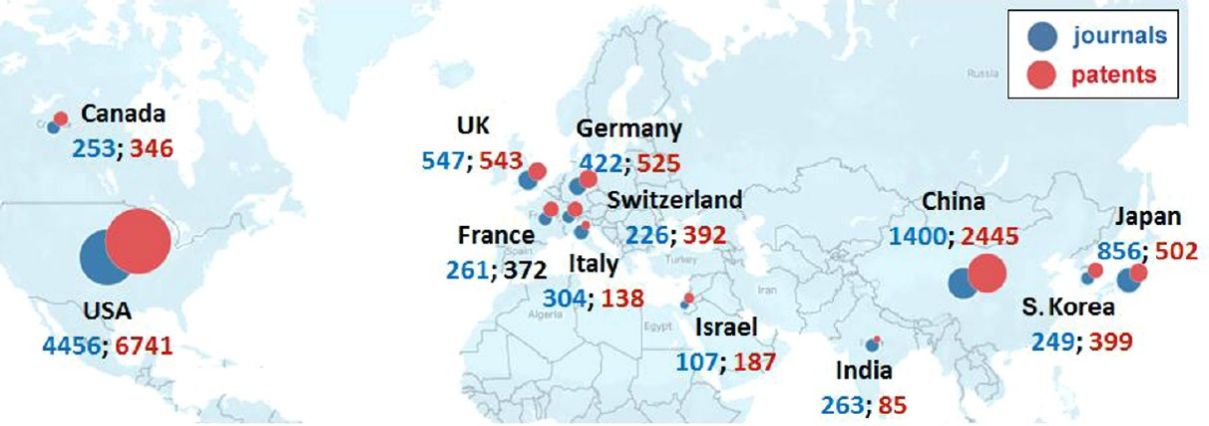
Top countries with respect to the number of ADC-related journal articles (blue) and patents (red).

**Figure 6:**
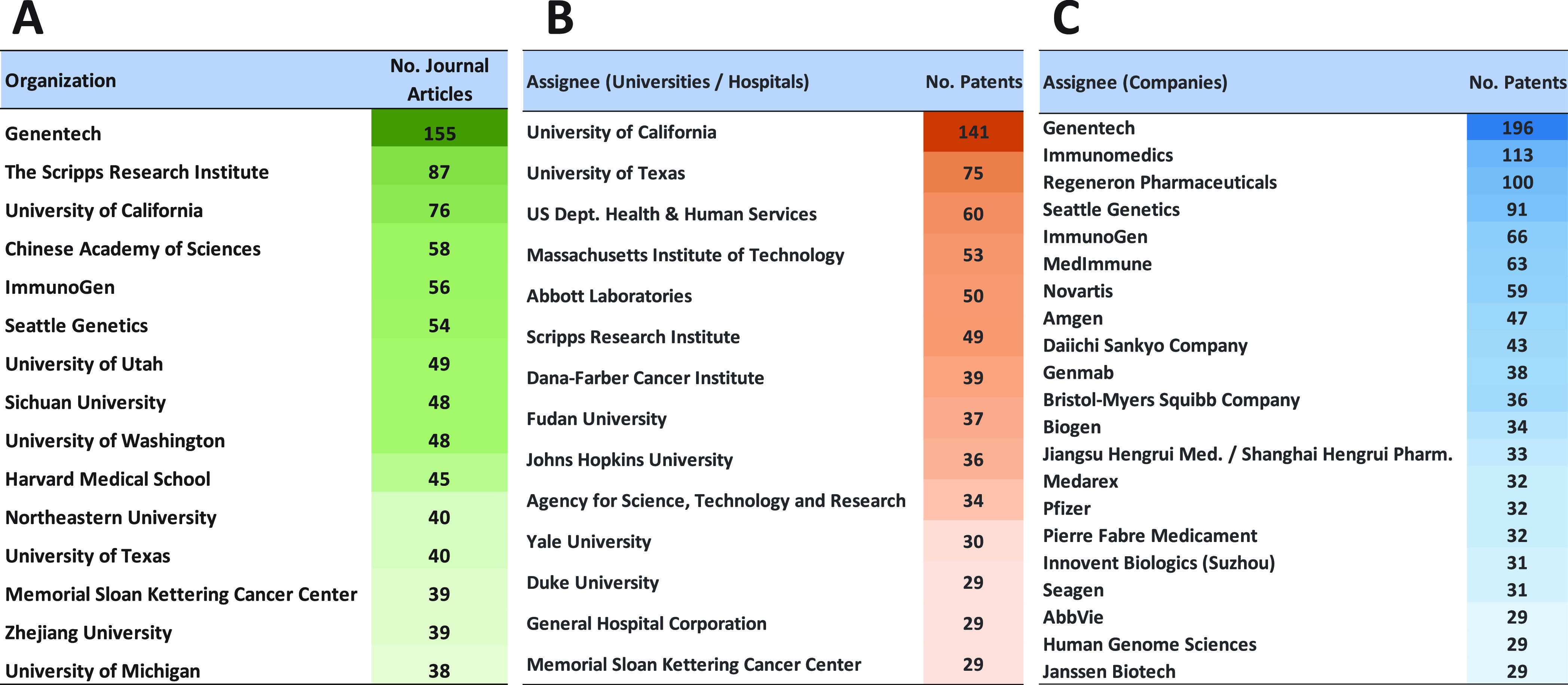
(A) Top organizations publishing ADC-related journal articles. Top patent assignees of ADC-related patents from universities and (B) hospitals and (C) companies.

**Figure 7:**
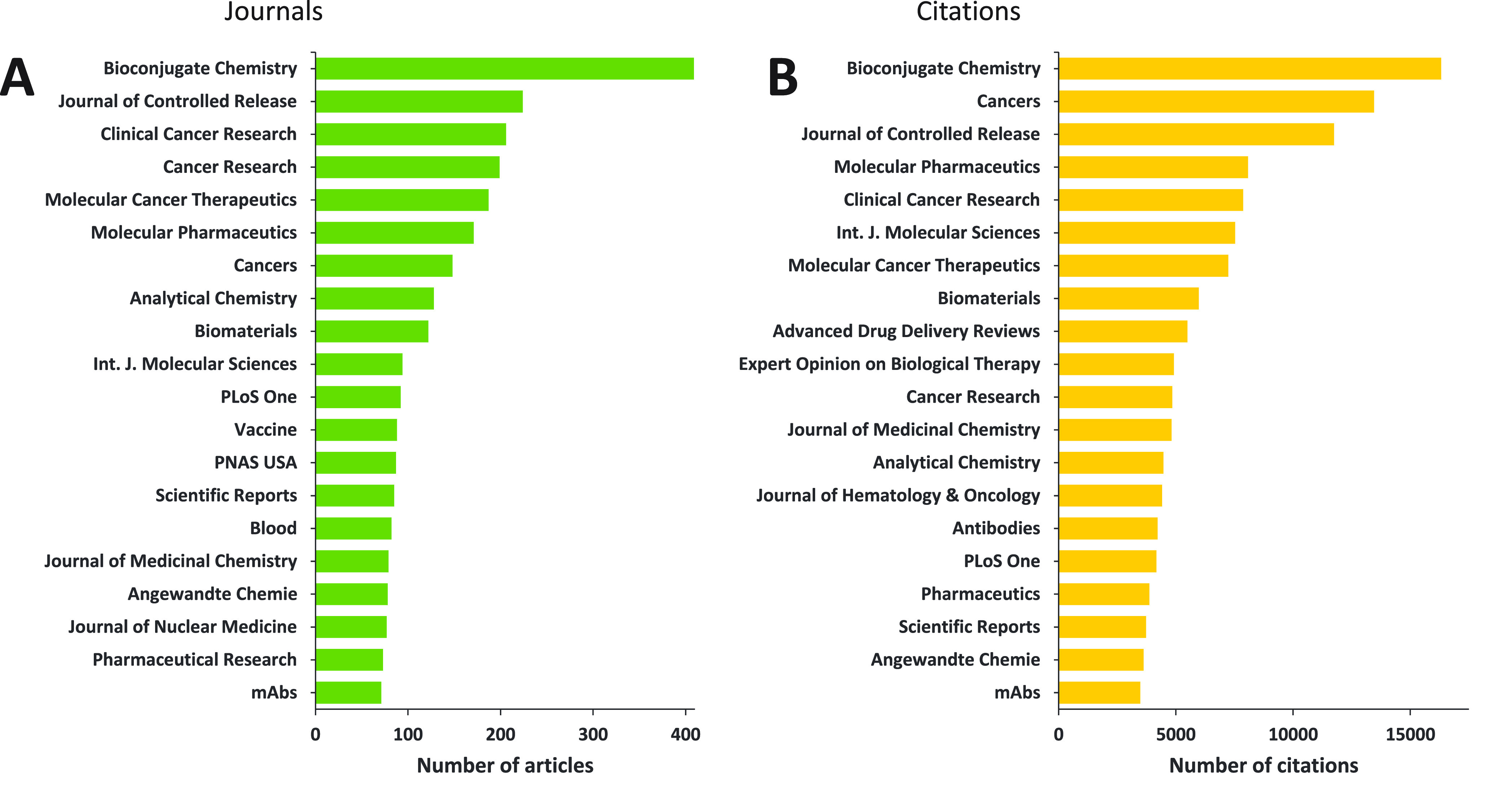
Top scientific journals with respect to the number of ADC-related (A) articles published and the (B) citations they received.

United States, China, Japan, United Kingdom, Germany, and South Korea are the leaders with respect to the number of published journal articles and patents related to ADC research with the United States having ∼3- and ∼2.7-fold greater number of journal and patent publications, respectively, as compared to China (Figure 5). Genentech, the Scripps Research Institute, University of California, and the Chinese Academy of Sciences have the largest number of published articles in scientific journals (Figure 6A). The journal *Bioconjugate Chemistry* publishes the most articles related to ADC research (Figure 7A) and is the most-cited journal for ADC research (Figure 7B). Unsurprisingly, patenting activity is dominated by corporate players as compared to academics (Figure 6B,C). Genentech, Immunomedics, Regeneron Pharmaceuticals, and Seattle Genetics have the highest number of patents among the companies (Figure 6C), while University of California leads among the universities, having nearly double the number of patents as University of Texas, ranked second (Figure 6B).

**Figure 8:**
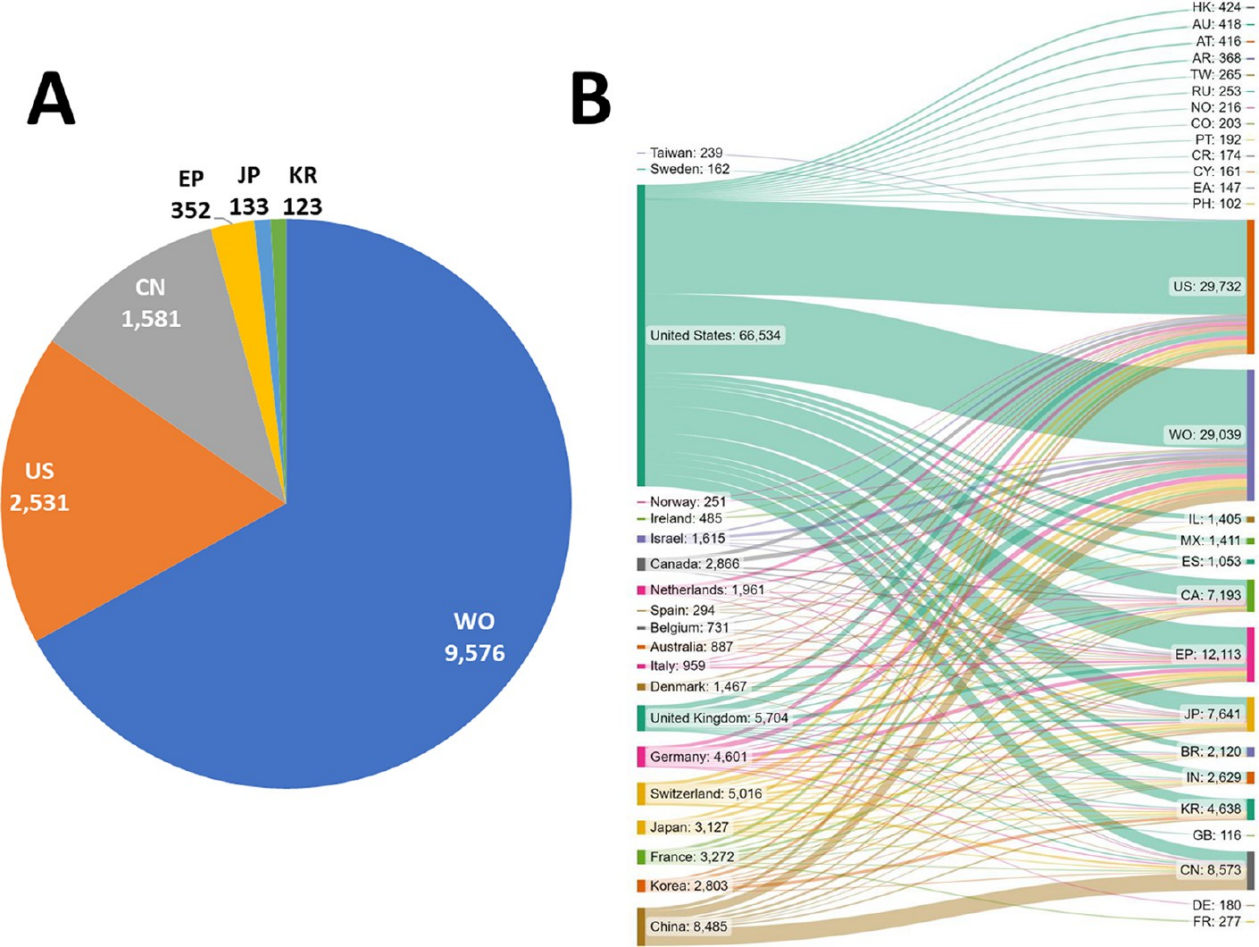
(A) Top patent offices receiving ADC-related patent applications. (B) Flow of ADC-related patent filings from different patent assignee locations (left) to various patent offices of filing (right). The abbreviations on the right indicate the patent offices of Hong Kong (HK), Australia (AU), Austria (AT), Argentina (AR), Taiwan (TW), Russian Federation (RU), Norway (NO), Colombia (CO), Portugal (PT), Costa Rica (CR), Cyprus (CY), Eurasian Patent Organization (EA), Philippines (PH), United States (US), World Intellectual Property Organization (WO), Israel (IL), Mexico (MX), Spain (ES), Canada (CA), European Patent Office (EP), Japan (JP), Brazil (BR), India (IN), South Korea (KR), Great Britain (GB), China (CN), Germany (DE), and France (FR).

Figure 8A presents the distribution of patents among the top patent offices receiving ADC-related patent applications. The World Intellectual Property Organization (WIPO) patent office clearly dominates accounting for about 2/3 of patents filed, followed by the patent offices of the United States (US), China (CN), Japan (JP), and S. Korea (KR), and the European patent office (EP).

Patent protection is territorial, and therefore the same invention can be filed for patent protection in several jurisdictions. We thus searched for all related files pertaining to ADCs. Certain patent families might be counted multiple times when they have been filed in multiple patent offices. Figure 8B presents the flow of patent filings from various applicant locations to a variety of patent offices of filing. Most of the applicants tend to have a comparable number of filings in their home country and at the WO, while also having a sizable number of filings at other patent offices such as the US, European Patent Office (EP), and others.

**Figure 9:**
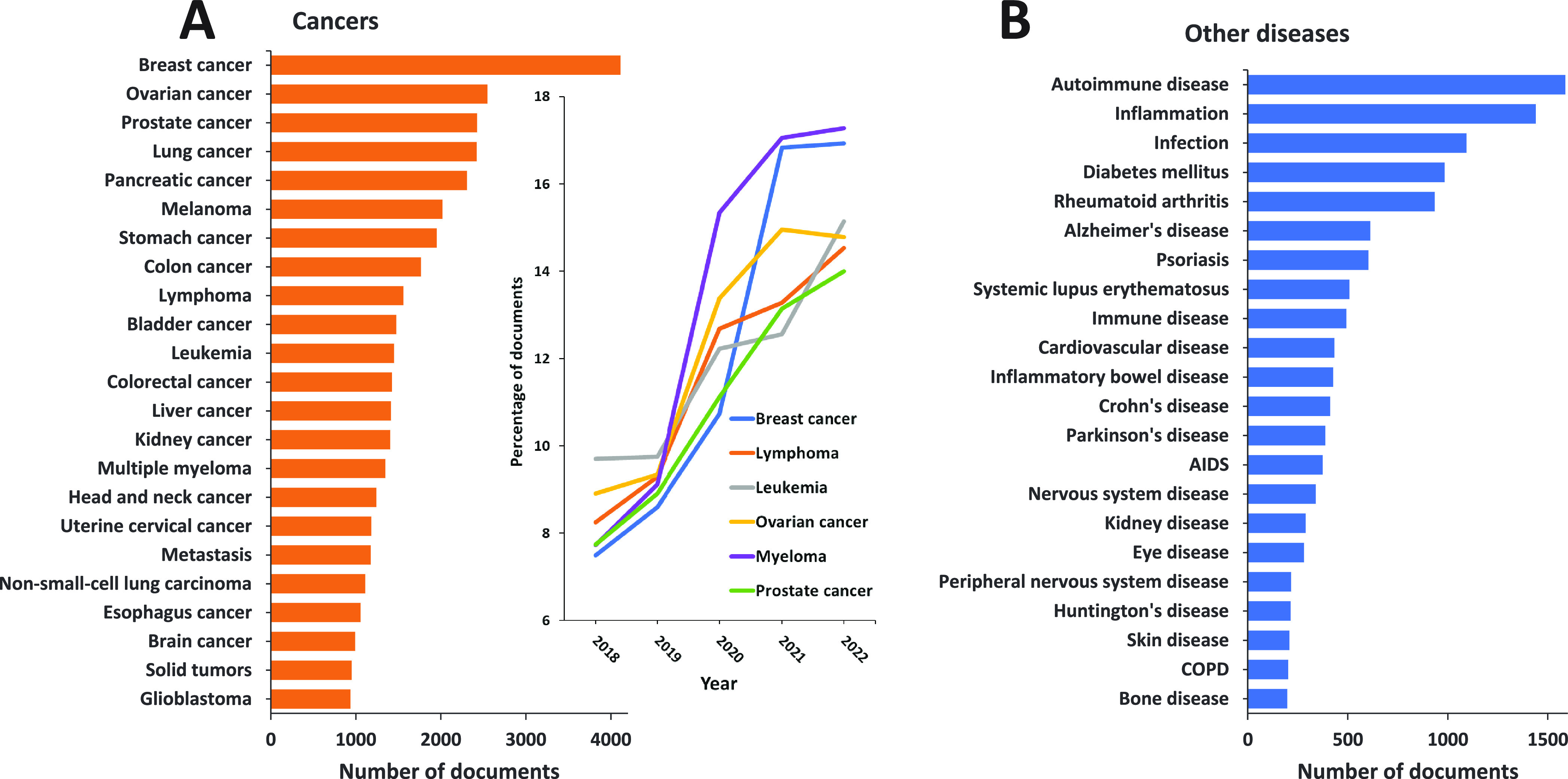
Diseases explored in ADC-related publications: (A) cancers (Inset: Annual growth of the number of documents for the fastest growing solid and hematological cancers for the years 2018–2022); (B) other diseases.

We further explored distribution and trends in the published documents (journals and patents) dealing with various ADC-related concepts. Figure 9 presents a number of the ADC-related documents in the CAS Content Collection concerning neoplastic (A) and other diseases (B). The highest number of documents pertain to breast cancer (mammary gland neoplasm) and lymphoma for solid tumors and hematological malignancies, respectively. This data correlates well with approved ADCs used in the treatment of cancers that are currently on the market.^53,55^ Breast cancer and myeloma exhibit the highest growth rate in the last five years with respect to the number of documents related to them (Figure 9A, inset). Most ADCs developed thus far, aimed at treating various types of cancer (solid and hematological), have nonetheless been restricted to treating cancer. Challenges in designing ADCs for noncancerous diseases include identifying targeting cell types, a specific surface marker expressed on the targeting cells, and an effective payload drug. So far, not many ADCs have been designed for noncancerous indications, with none yet having successfully progressed through clinical trials to market. With the advancement of ADC platforms and technology, more ADCs for nononcology indications are being developed.^272^ A search in the CAS Content Collection showed that among the noncancerous diseases, autoimmune diseases, inflammations, and infections are the top pathologies with respect to the number of documents related to ADCs (Figure 9B). Despite obvious complications such as difficulty in penetration through the blood-brain barrier (BBB), our data indicate a growing interest in development of ADCs targeting the brain. The number of documents pertaining to ADCs in the context of neurodegenerative disorders such as Alzheimer’s, Parkinson’s and Huntington’s disease (Figure 9B) are on the rise. Recent approvals of antibody treatments for Alzheimer’s disease by the US FDA^273,274^ also stimulate ADC development for neurological diseases.

**Figure 10:**
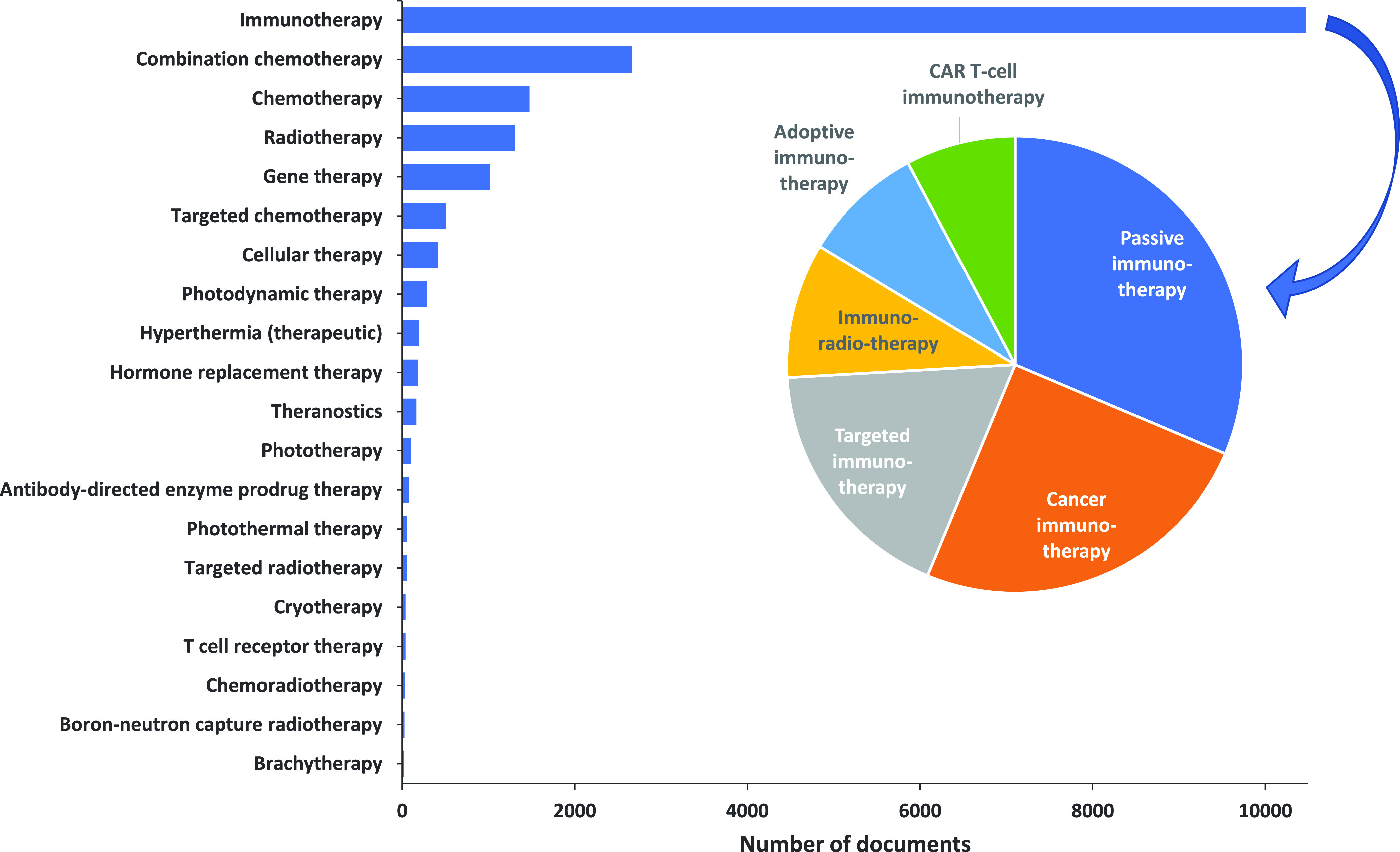
Therapies explored in the ADC-related publications.

Figure 10 presents the number of ADC-related documents in the CAS Collection concerning various types of therapies. Immunotherapy understandably accounts for the highest number of documents. Indeed, a sound biological rationale supports the research into combining ADCs with immunotherapy to overcome the incidence of resistance and improve patient outcomes.^275^ Most immunotherapy-related papers involve passive immunotherapy (Figure 10, inset pie chart). Passive immunotherapy agents produce rapid antitumor responses by direct administration of immune-cell factors, such as cytokines or antibodies. With passive immunotherapy, continued dosing may be required for a prolonged response since immune system memory is not engaged.^276,277^

**Figure 11:**
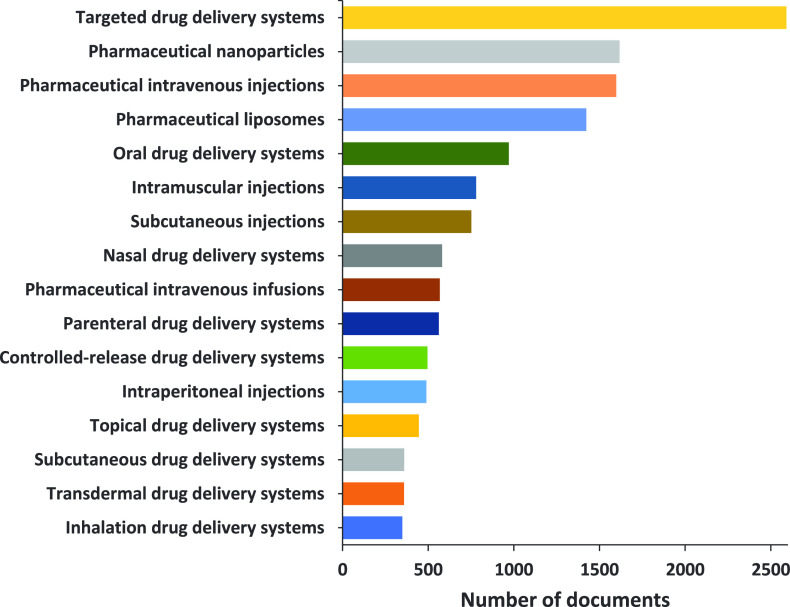
Drug delivery systems explored in the ADC-related publications.

Targeted intravenous drug delivery systems for nanoparticles are the most common drug delivery systems explored for ADCs (Figure 11). Indeed, intravenous administration into the bloodstream is the preferred route for ADCs in order to avoid digestion of antibodies by gastric acid and proteolytic enzymes with oral administration.^278^ At present, all approved ADCs are administered via the intravenous route, and the therapeutic capacity of other routes of ADC administration is rarely explored. Subcutaneous, intramuscular, intravitreal, inhalable, intra-articular, and intratumoral drug delivery methods have all been used to improve the therapeutic indexes of antibodies,^279^ with subcutaneous and intratumoral routes considered especially promising.^280,281^ However, with respect to ADCs, there is still a need to explore and evaluate the therapeutic potential of alternative routes of administration.

**Figure 12:**
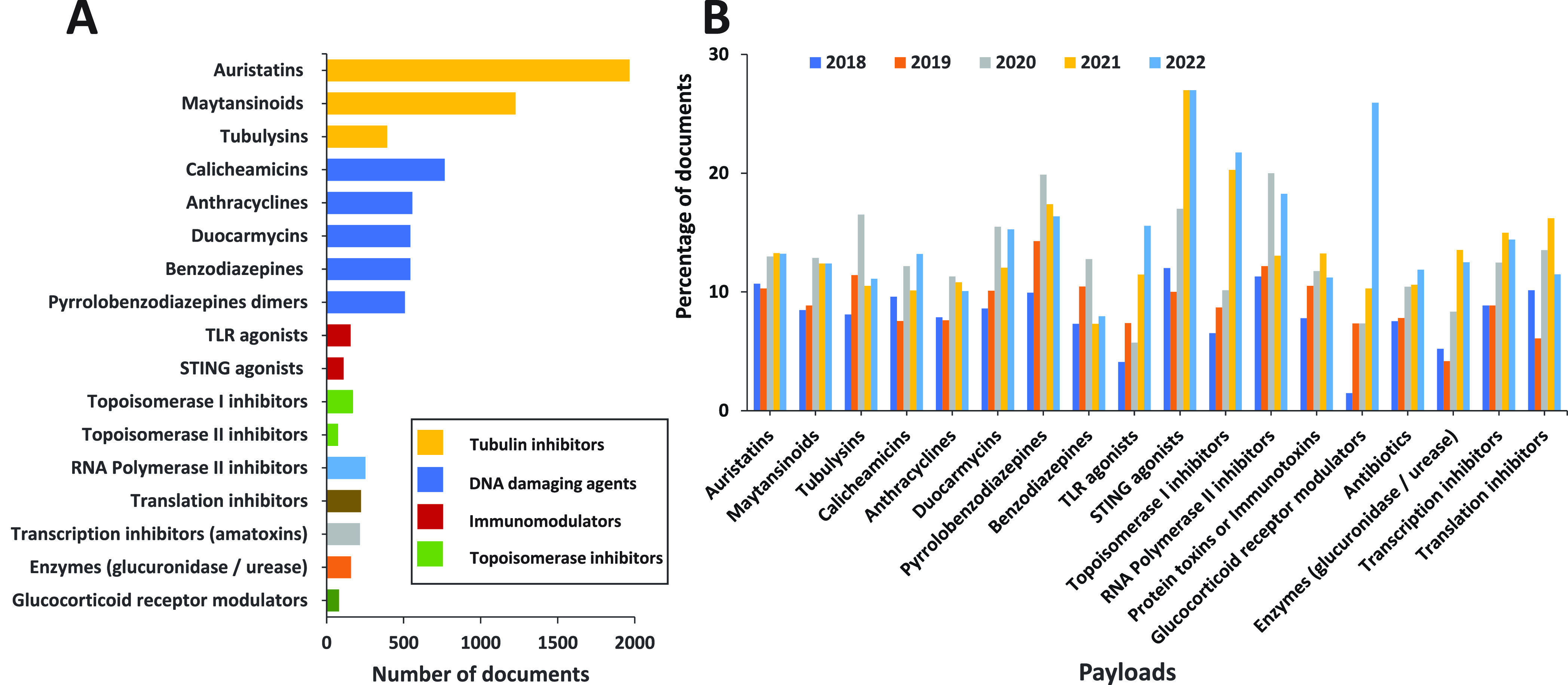
ADC payloads explored in the scientific publications: (A) Number of publications exploring ADC payloads. (B) Trends in number of publications exploring ADC payloads during the years 2018–2022.

Cytotoxic payloads are major components of ADCs, with the tubulin inhibitors and DNA damaging agents being the most widely explored, and until recently the major classes of compounds used in ADCs.^17,19,22,88,127^ With the approval of trastuzumab deruxtecan (Enhertu) in 2019, the diversification of ADC payloads has been appreciated as a key approach in the ADC development, and a number of new classes of compounds have been examined as potential payloads.^282^ In the CAS Content Collection, in terms of the number of published documents, auristatins and calicheamicins are the major representatives of tubulin inhibitors and DNA damaging agents, respectively (Figure 12A). As a sign of the payload diversification efforts, STING agonists, glucocorticoid receptor modulators, and topoisomerase I inhibitors exhibit the fastest consistent yearly growth in the number of documents (Figure 12B). The success of immune checkpoint inhibitors targeting the adaptive immune system has greatly stimulated interest in exploring immune-stimulating ADC payloads such as STING agonists and TLR agonists.^282^ Glucocorticoid receptor modulators are the primary treatment for various immune diseases, and targeted delivery via an ADC may provide significant efficacy at doses that do not lead to unwanted side effects.^283^ Topoisomerase I inhibitors, particularly camptothecin analogs, constitute a successful ADC payload family, and are a part of two recently FDA approved drugs, trastuzumab deruxtecan, and sacituzumab govitecan.^284,285^

**Figure 13:**
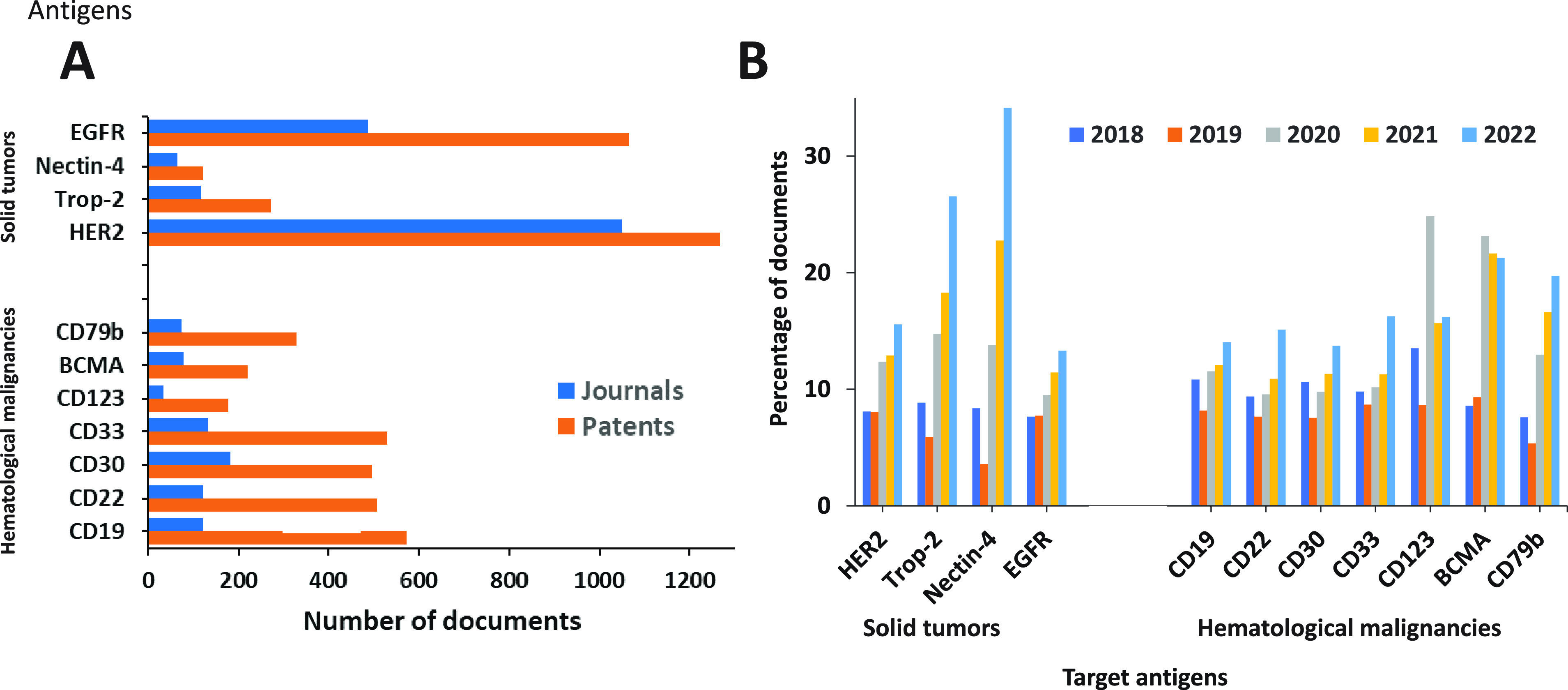
ADC target antigens explored in the scientific publications for solid tumors and hematological malignancies: (A) Number of publications exploring target antigens. (B) Trends in growth of publications exploring target antigens during the years 2018–2022.

The number of documents and growth trends related to target antigens of ADCs according to the CAS Content Collection are listed in Figure 13. While HER2 and EGFR remain the most widely explored target antigens for solid tumors (Figure 13A), Trop-2 and Nectin-4 antigens have exhibited consistent and steady growth in the last five years (Figure 13B).^286,287^ For hematological malignancies, CD30, CD19, CD22, and CD33 are the most extensively examined with almost triple the number of patents as compared to journal articles (Figure 13A), while CD79B shows the fastest growth in number of documents over the last five years (Figure 13B).

**Figure 14:**
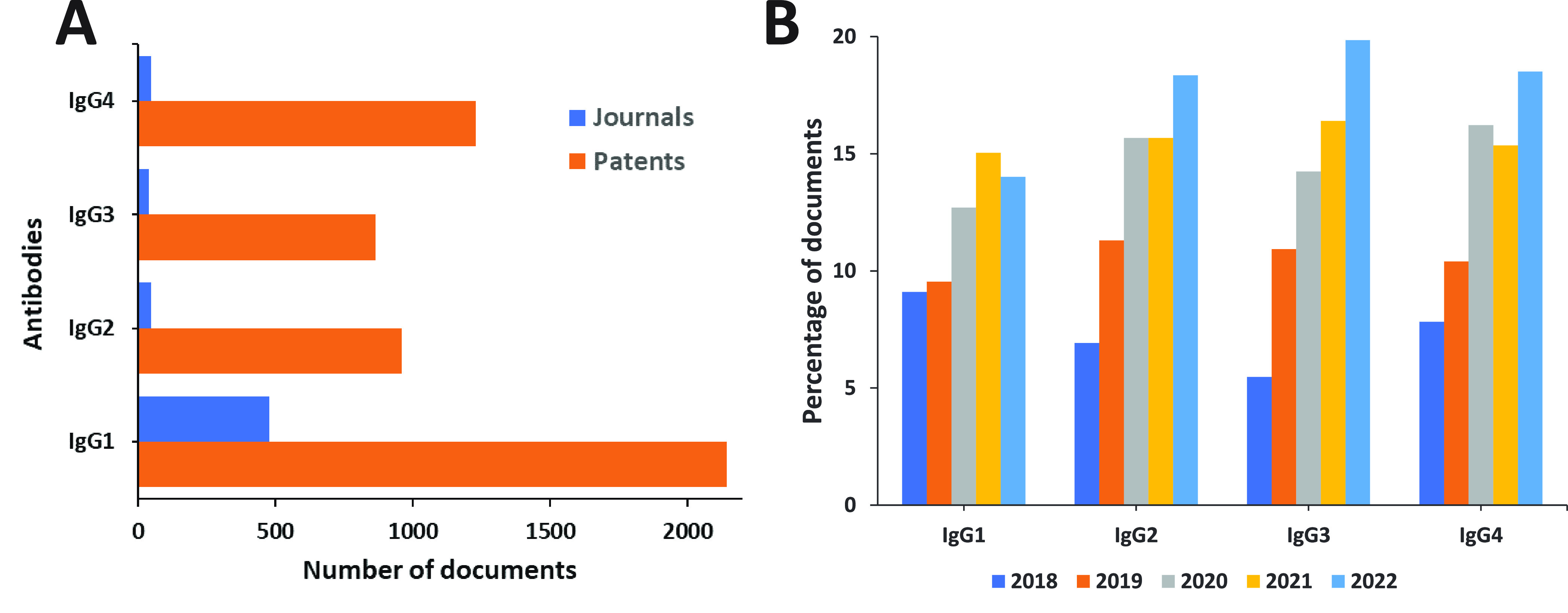
ADC antibodies explored in the scientific publications: (A) Number of publications exploring ADC antibodies. (B) Trends in number of publications exploring ADC antibodies during the years 2018–2022.

We also examined the distribution and trends in published documents pertaining to antibodies used in ADCs, especially the immunoglobulin G (IgG) isotype, which is the most frequently used isotype for cancer immunotherapy (Figure 14). IgG1 and IgG4 are the IgG subtypes that are used in most formulations (and the only ones used in approved ADCs) in line with established knowledge, with the number of patents clearly dominating journal articles by ∼4- and 25-fold, respectively (Figure 14A). Although the four subclasses of IgG have >90% homology, they exhibit distinctive profiles with respect to the hinge region length, the number of interchain disulfide bonds, and Fc-effector functions.^288^ IgG3 displays the highest affinity binding to most Fc-γ receptors, but is susceptible to proteolysis and aggregation and is avoided in ADC design due to its short circulating half-life.^289^ Of the remaining subclasses, IgG1 demonstrates the highest affinity for all Fc-γ receptors. Publications discussing the use of IgG2 and IgG3 in ADCs have increased more rapidly in recent years than those discussing the use of IgG1 (Figure 14B).

**Figure 15:**
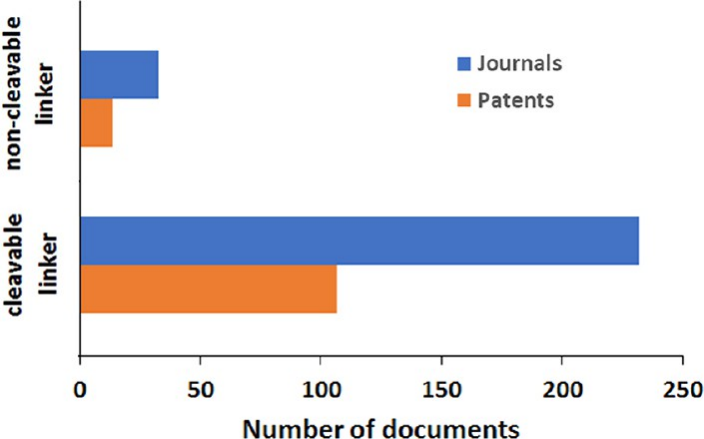
Antibody-payload linker types explored in scientific publications.

Figure 15 shows the antibody-payload linker types as represented in the CAS Content Collection. The cleavable linkers markedly dominate; they are the preferred linker type in the therapeutic ADCs because of their versatility, providing possibility for a controlled payload release.

**Figure 16:**
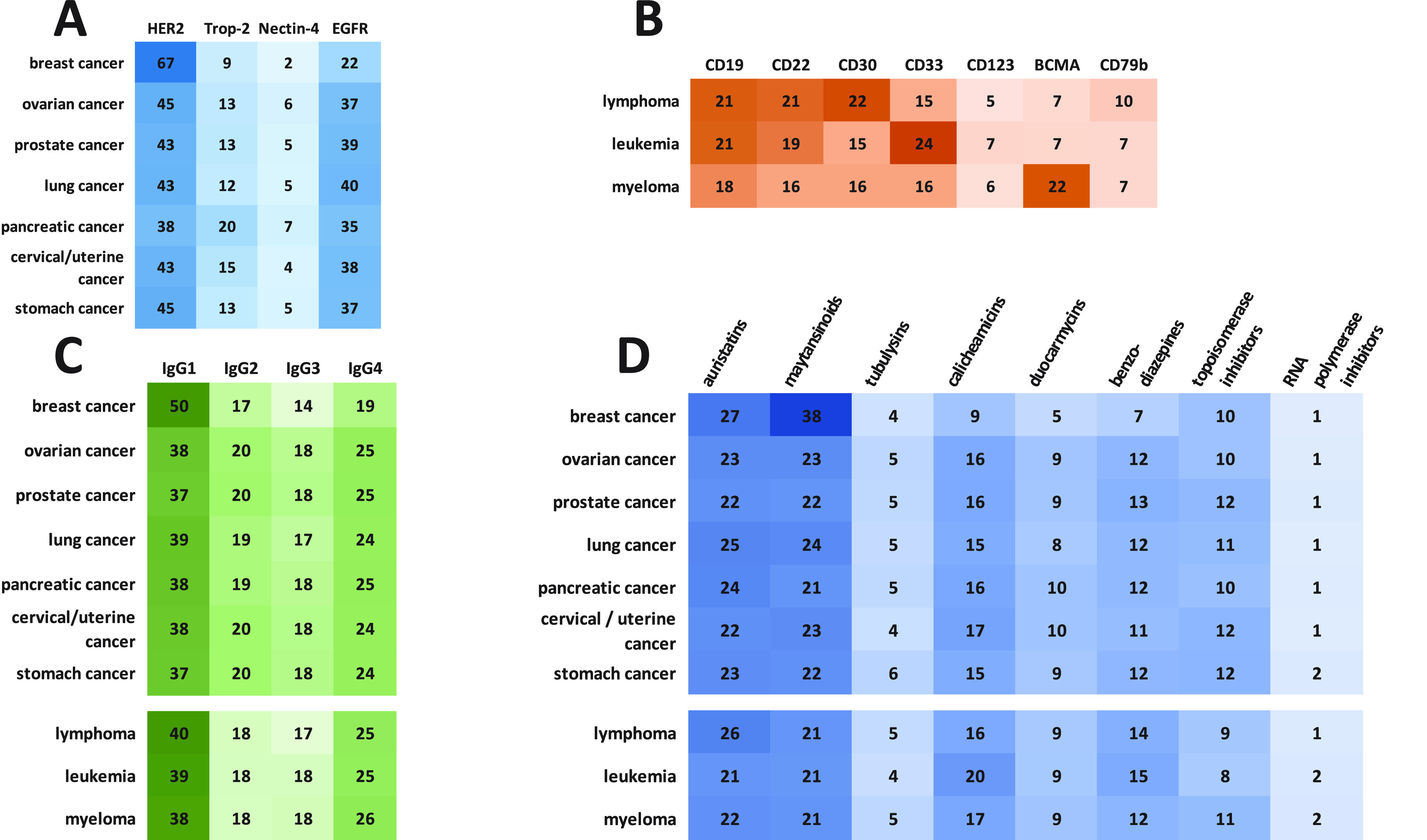
Correlations between different concept pairings are shown as heat maps. ADC target antigens and (A) solid tumors and (B) hematological malignancies. (C) ADC antibodies and types of cancers and (D) ADC payloads and types of cancers (numbers represent percentage of documents related to the given disease). Darker shades correspond to a higher number.

Figure 16 presents correlations in the examined documents between various cancers and ADC target antigens (Figure 16A and B), ADC antibodies (Figure 16C), and ADC payloads (Figure 16D), calculated as the percentage of documents related to the given disease. With solid tumors (Figure 16A), the strongest correlation is between breast cancer and HER2 as target antigen, while among hematological malignancies (Figure 16B) lymphoma correlates strongly and comparably with CD19, CD22, and CD30, leukemia–with CD33 and CD19, and myeloma, with BCMA. With respect to ADC payloads (Figure 16D), the strongest correlation is between breast cancer and the class of maytansinoids, followed by auristatins with similar trends observed for lymphoma.

**Figure 17:**
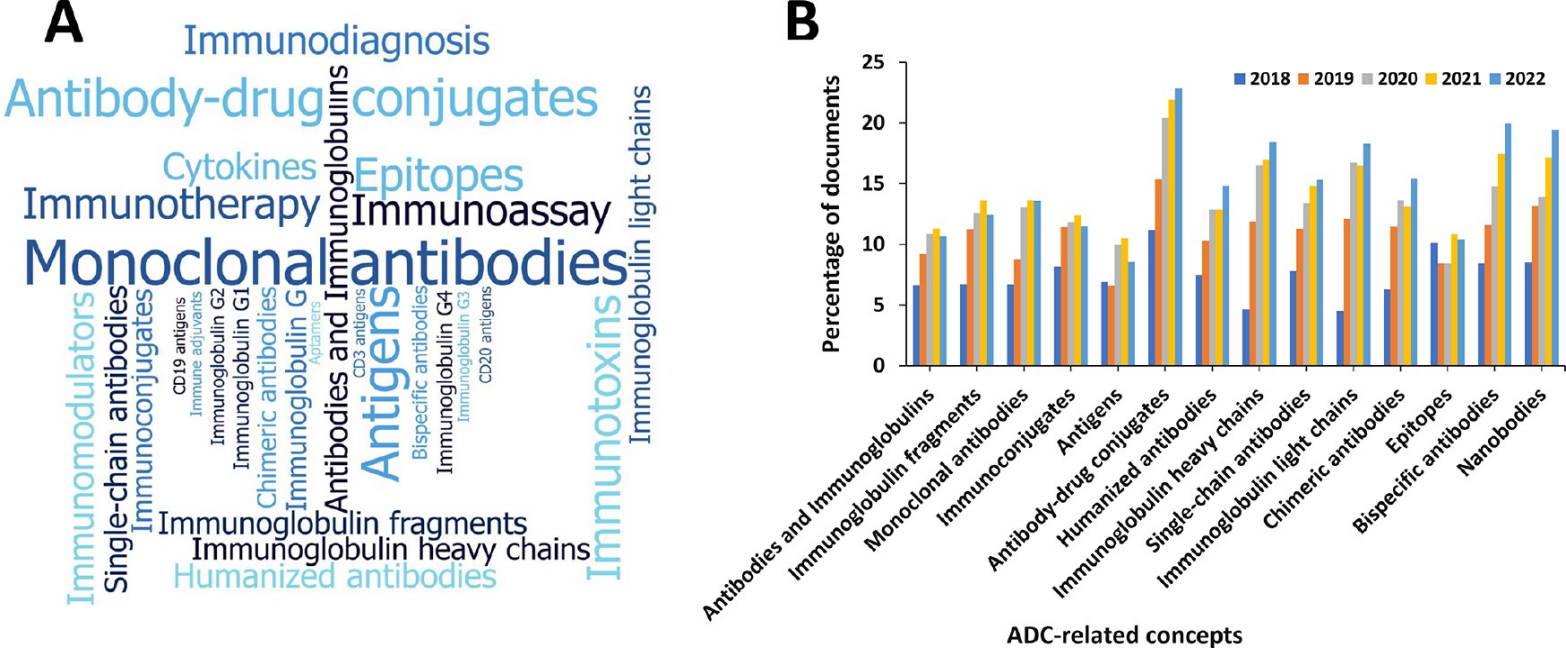
(A) A word cloud of the most widely used ADC-related concepts in the CAS Content Collection. (B) Yearly growth in the number of documents (percentage) over the 2010–2022 period for ADC-related concepts.

Among the ADC-related concepts in the CAS Content Collection, monoclonal antibodies is clearly the major one, along with antibody–drug conjugates, and antigens (Figure 17A). Bispecific antibodies and nanobodies, along with the antibody–drug conjugates, exhibit the fastest growth rate with respect to the number of published documents (Figure 17B).

**Figure 18:**
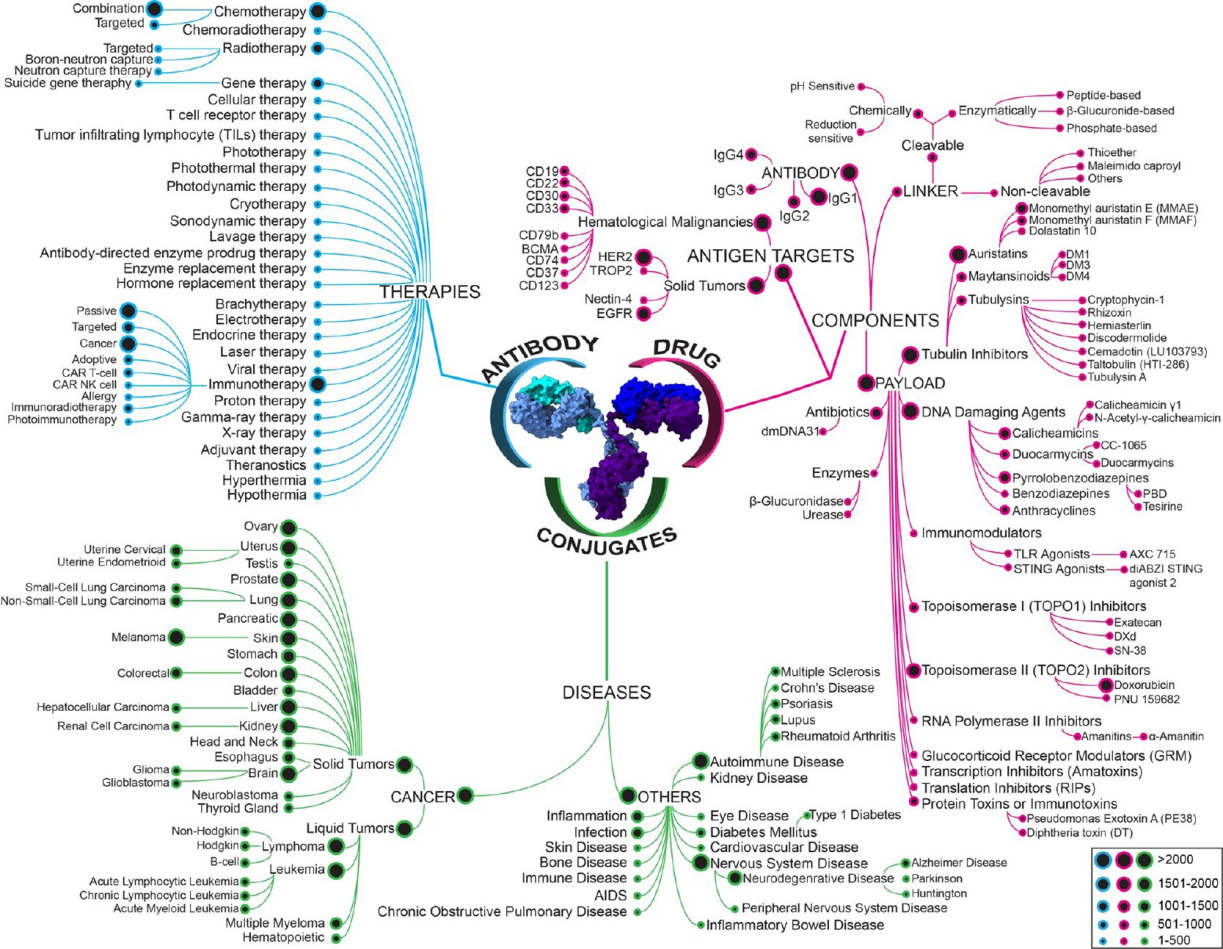
ADC Concept Map. Size of the dot at each concept/topic corresponds to the number of documents (journals and patents) in the CAS Content Collection related to the given concept/topic.

Figure 18 represents a map of the ADC-related concepts with an indication of the number of documents in the CAS Content Collection related to the given concept/topic.

## 4 ADC-Based Combination Therapies

Development of ADC resistance is a major drawback to their therapeutic potential. Many mechanisms of resistance exist^290^ including:

- Downregulation of/change in antigen expression^291,292^ – decreases the binding of an ADC to the target antigen and might even increase toxicity due to prolonged presence of ADC in the bloodstream.
- Decreased internalization of the ADC-bound antigen^291,292^ – could result from increased recycling of the target antigen preventing release of cytotoxic load in desired locations.
- Inefficient binding of the ADC to its target antigen^291,292^ – resulting from changes in the target (such as truncation) or increased interactions with other binding partners reducing affinity of ADC for target antigen.
- Inefficient/incomplete/improper degradation of ADCs inside lysosomes leading to poor release profiles of cytotoxic payloads and reducing therapeutic effectiveness.^293^
- Poor release from lysosomes^292^ – many cytotoxic drugs tend to be charged molecules that require active transport from the lysosome into the cytoplasm and change in expression of lysosomal transporters which can affect concentration of payloads achieved in the cytoplasm.^267^
- Overexpression of efflux pumps such as multidrug resistant transporter 1 (MDR1) that actively transport the released cytotoxic payload from the cytoplasm to outside of the tumor cell reducing therapeutic effectiveness of ADC.^292^

These resistance mechanisms reduce the effectiveness of ADCs in cancer therapy. The simplistic but vital reasoning behind use of combination therapy is to circumvent drawbacks (such as resistance mechanisms) of both types of therapies (including reducing toxicities associated with them) to boost the overall therapeutic efficacy and is especially true for an aggressive illness such as cancer with a high mortality rate.^294^

### 4.1 ADC and Conventional Chemotherapy

Conventional cancer therapies tend to include a combination of chemotherapeutic drugs, radiation therapy and surgery.^295^ Classical chemotherapeutic drugs such as altretamine^296^ and gemcitabine^297^ exert their antitumor effects by alkylating or cross-linking DNA.^298,299^ or by shutting down DNA synthesis by competing with endogenous nucleotides.^300^ While effective, conventional chemotherapeutic drugs are toxic, show significant side effects because of insufficient selectivity^301^ and are subject to a variety of resistance mechanisms.^302^ All of these factors greatly reduce their effectiveness. Combining a targeted approach such as ADCs with standard or conventional untargeted chemotherapeutic approaches has been shown to overcome the drawbacks and improve survival rates.^303^ Perhaps the most well-known chemotherapeutic-ADC combinations involve gemcitabine, a nucleoside analog that exerts antitumor activity by interfering with DNA synthesis.^300^ An early study of an ADC-chemotherapeutic drug combination involved gemcitabine and brentuximab vedotin (SGN-35), a CD30 targeted ADC^304^ and this combination is still being tested for the treatment of various types of cancers.^305−307^ While no rationale has been given for the effectiveness of the gemcitabine/brentuximab vedotin combination, one hypothesis is that brentuximab vedotin and gemcitabine target neoplastic Reed Sternberg cells and stromal cells, respectively and exert synergistic effects.^304^ Expression levels of surface antigens are crucial for the success of ADC therapy, and changes in their levels are associated with decreased therapeutic efficacy. Gemcitabine administration has been linked to increased expression of HER2 by as much as 2.5-fold.^308^ Co-administration of gemcitabine with trastuzumab emtansine, a HER2-specific ADC, boosted the antitumor effect achieved by the ADC in pancreatic ductal adenocarcinoma.^308^ Other instances of synergistic effects have been observed when gemcitabine was coadministered with camidanlumab tesirinean (ADCT-301),^309^ an ADC specific for CD25 (interleukin-2 receptor alpha chain)^310^ or with Oba01 ADC, an ADC targeting death receptor 5 (DR5).^311^ Other chemotherapeutic drugs that are being explored include alkylating agents cisplatin,^312−314^ carboplatin^315−317^ and cyclophosphamide (ClinicalTrials.gov ID: NCT01042379)^318^ and topoisomerase II inhibitor doxorubicin^317^ in combination with ADCs whose targets are cell surface glycoproteins such as LYPD3,^312^ CD205^313^ and LRG1^314^ among others.

ADC combinations with not only chemotherapeutic agents but also antibody-based therapy have been also explored. Thus, antiangiogenic agents can stimulate ADC penetration and tumor cell exposure. Combination of anetumab ravtansine or mirvetuximab soravansine with bevacizumab has enhanced efficacy in preclinical models of ovarian cancer. A recent study combined mirvetuximab soravtansine and bevacizumab in ovarian cancer patients with encouraging results in the pivotal AURELIA trial.^319^ Anetumab ravtansine in combination with bevacizumab has been tested for treatment of ovarian cancer.^319^ Clinical trials such as KAITLIN, KRISTINE, and MARIANNE were designed on the basis of synergistic antitumor activity with trastuzumab emtansine in combination with pertuzumab.^319^

### 4.2 ADC and Immune Checkpoint Inhibitors

Immune checkpoint molecules are proteins that regulate the magnitude, type and duration of immune response^320,321^ they are classified as either inhibitory or stimulatory depending upon the pathways they influence.^322^ Meant to act as guards against excessive immune response,^323^ they can also act as avenues for tumor cells to escape immunosurveillance.^320,324^ A few well-known examples of inhibitory immune checkpoint molecules include programmed cell death protein-1 (PD-1),^325^ cytotoxic T lymphocyte-associated antigen-4 (CTLA-4; also known as B7, CD152),^326^ B and T lymphocyte attenuator (BTLA; also known as CD272),^327^ and lymphocyte-activation gene 3 (LAG-3; also known as CD223)^328,329^ among others. For the stimulatory pathways, glycoproteins such as OX40 receptors (also known as CD134 and TNFRSF4)^330,331^ and 4–1BB (also known as CD137)^332^ are few well-known examples of immune checkpoint molecules. Expressed on cell surface, immune checkpoint molecules modulate the activity of T cells^320^ and it is this feature that can be exploited to our advantage. Inhibition of immune checkpoint inhibitors reduces the suppression of immune response to cancers, activating T cells to kill cancer cells.^333,334^ Alternatively, activating stimulatory immune checkpoint molecules either by preventing interactions with their endogenous ligands or by directly activating them achieves the same effect, i.e., T cell activation and proliferation resulting in increased antitumor effects.^335−338^ Immune checkpoint molecules are successful targets for FDA approved immunotherapeutic drugs such as the PD-1 inhibitors pembrolizumab (Keytruda)^339^ and cemiplimab (Libtayo),^340^ and the CTLA-4 inhibitors ipilimumab (Yervoy)^341^ and recently approved tremelimumab (Imjudo).^342^ In addition, CA-170 (a small molecule targeting PD-L1, PD-L2 and VISTA),^343^ ieramilimab/LAG252 (targeting LAG-3)^344^ (ClinicalTrials.gov ID: NCT02460224^345^), and sabatolimab/MBG453 (targeting TIM-3)^346,347^ (ClinicalTrials.gov ID: NCT04266301^348^) are examples of immunotherapeutic agents currently in clinical trials.

The development of immune checkpoint modulators has been immensely useful especially in the treatment of solid cancers such as nonsmall cell lung carcinoma (NSCLC)^349^ and melanoma^350,351^ among others. Long-term remission, a feat that was considered very hard if not impossible to achieve, has been made possible by immune checkpoint inhibitor (ICI) therapy.^352^ Unfortunately, the fraction of patients that actually show such long-term remission remains small.^353^ A recent study^354^ suggests that immune checkpoint inhibitor therapies become ineffective against chemotherapy-treated triple-negative breast cancer because TP53 mutations alter the expression levels of immune checkpoint molecules, allowing tumor cells to undergo “immune exclusion”. As with other treatment modalities, resistance reduces the effectiveness and utility of immune checkpoint inhibitors.^355^ As such, they are often used in combination^356,357^ with either other immune checkpoint modulators^358^ or conventional chemotherapeutic agents to boost their therapeutic efficacy.^359^ More recently, there has been an increasing use of ADCs in combination with immunotherapeutic agents.^360,361^ The ability of ADC to trigger immune responses to cancer cells suggests that they could exhibit synergism with immune checkpoint modulators.^360^

The most often used immunotherapeutic agents in combination with ADCs appears to be confined to PD-1 inhibitors with pembrolizumab^316,362−364^ and nivolumab^365−370^ dominating and followed by atezolizumab,^371,372^ durvalumab^373−375^ and toripalimab^376−380^ and are currently in various stages of clinical trials. A few representative examples of patents for ADC immunotherapy combination therapy are WO2018160538,^381^ US20220133902,^382^ WO2022242692,^383^ WO2018110515.^384^ Most ADCs used in these combination therapies appear to be directed toward HER2^371,380,385−388^ with a smaller number directed toward targets such as LIV-1,^389^ nectin-4,^390^ TROP2^391,392^ and B7–H3 (also known as CD276).^393^ Recently, ADCs have been developed to target immune checkpoint molecules such as PD-L1^394^ and B7–H3^395^ by acting both to prevent immune suppression by binding directly to the immune checkpoint molecules and to carry cytotoxic payloads. A recent example of such a bifunctional ADC was a small molecule immunomodulator attached to an anti-PD-L1 antibody via a cleavable disulfide linker, dubbed an immune modulating ADC (IM-ADC).^396^ The released payload was found to exert its antitumor effect by inducing CD8^+^ and CD4+ T cytotoxic lymphocyte infiltration.^396^ In addition, the payload increased PD-L1 expression, especially in tumor cells, thereby increasing the effectiveness of subsequent rounds of treatment with the same ADC.

Tumor associated macrophages (TAMs) are an important part of the tumor microenvironment (TME) and have been linked to protumor effects including initiation^397−399^ and progression^400^ among others. The presence of TAMs has been linked to the antitumor efficacy of the anti-CD-30 antibodies, SGN-30^401^ and SGN-40.^402^ TAMs have been shown to enhance the uptake of a nontargeted ADC and the release of its payload, leading to an enhanced bystander effect^403^ which enhances its toxicity to tumor cells with low or variable antigen expression. A major difference between the two macrophage subtypes – M1 and – M2 lies in their functional activities. M1 and M2 macrophages are associated with antitumor (cell death) and pro-tumor (cell proliferation) effects, respectively.^404^ Efforts have been made to reprogram M2 TAMs to act similar to antitumor M1 TAMs by utilizing a peptide.^405^

### 4.3 Sequential/Staggered Therapy

While combination therapies use multiple drugs or therapies simultaneously, sequential or staggered therapies use the sequential administration of multiple anticancer therapies in a specific order interspersed with pauses to maximize their antitumor effects while minimizing their toxicities.^406^ Growing literature evidence suggests that in some instances sequential therapy may be more effective than combination therapy, especially immunotherapies,^407^ and that the order of administration plays a crucial role in its success.^408^ Sequential therapy have shown promise in breast cancers,^409^ renal cell carcinomas,^410^ and lung cancers.^411^ The cBR96-doxorubicin ADC was shown to be more effective when administered before the tubulin inhibitor paclitaxel when compared to coadministration.^412^ The ADC consisting of a doxorubicin payload attached to the BR96 antibody via a cleavable hydrazone linker, increased the sensitivity of tumor cells to paclitaxel.^412^ The increased effectiveness of sequential therapy over combination therapy might be attributed to reduced internalization of the ADC by paclitaxel.^412^

## 5 ADCs beyond Oncology

Until recently, ADCs in preclinical and clinical development have been applied exclusively for oncology indications, with cytotoxic warheads targeted to antigen-expressing cancer cells. However, the concept of site-specific release of pharmacologically active small molecules for alleviating pathogenic cellular activities with minimal off-target effects by using an ADC-mediated delivery platform might also be effectively applicable for any disease area. Thus, over the recent years, researchers have examined opportunities to develop ADCs beyond cancer, for other disease indications including autoimmune disease, inflammatory and immune disorders, difficult-to-treat bacterial infections, and atherosclerosis, with payloads varying from glucocorticoid receptor modulators and kinase inhibitors to antibiotics and siRNA.^413−417^ Multiple factors may affect the effective development of an ADC including the choice of target, payload efficiency and mode-of-action, linker design, and conjugation method. With the growing knowledge of the mechanism of action of ADCs, it is highly anticipated that the development of such compounds in therapeutic areas outside of oncology and hematology will rapidly intensify.

### 5.1 ADCs against Infectious Diseases: Antibody–Antibiotic Conjugates

The effectiveness of antibiotic treatment for bacterial infections has been compromised by the development of widespread drug resistance. In response, antibody–antibiotic conjugates (AAC) were developed as a countermeasure.^418^ Analogously to ADCs where the antibodies are used to deliver cytotoxic drugs to the antigen-expressing cells, AACs use antibodies to deliver antibiotics to the target bacteria. These AACs combine the specificity of a bacterial antigen-specific monoclonal antibody with the bactericidal capacity of a potent antibiotic^419−421^ via a linker which ensures an efficient release of antibiotics at the target site. This design takes advantage of the improved absorption, distribution, metabolism, and elimination (ADME) properties of the antibodies.^178^ Antibiotics used for developing AACs must be highly potent, nonimmunogenic, soluble, and stable under physiological conditions.^421^

For example, Genentech has developed an AAC to combat hard-to-treat, invasive *Staphylococcus aureus* infections named DSTA4637S – an IV-administered THIOMAB-type AAC.^178^ It is composed of an IgG-type b-GlcNAc-WTA monoclonal antibody. The light chain of this antibody is connected to a rifamycin class antibiotic (dmDNA31) via a protease cleavable MC-ValCit-PABQ linker.^422,423^ This linker has a lysosomal protease-cleavable site, which helps in releasing the antibiotic payload inside the bacteria. Upon administration, the AAC prodrug enters the circulation; subsequently, the antibody portion of the AAC binds to bGlcNAc modification of teichoic acid (a polyanionic glycopolymer present in the peptidoglycan layer of the bacterial cell wall). This leads to the formation of a phagolysosome; the AAC is then opsonized into the intracellular environment. Once inside, host proteases cleave the linker releasing the dmDNA31 antibiotic exhibiting a strong bactericidal action against intracellular *S. aureus* bacteria (Clinical trial ID#: NCT03162250).^422^ Unfortunately, this AAC has been discontinued in clinical trials for undisclosed reasons, but provides inspiration for further antibacterial development.

More recently, AACs are being developed against bacterial biofilms,^424^ the hypothesis being that an antibody can be used to anchor the antibiotic to the surface of the bacteria within the biofilm. Using a trigger, such as an external small molecule, releases the antibiotic at the bacterial cell surface. Studies were done using mitomycin C, an antibiotic with a well-known antitumor effect^425,426^ which was found effective in controlling *S. aureus* biofilms *in vitro* and *in vivo*. Reduction of biofilms is important in reducing the population of persistent (difficult to kill) bacterial cells (rendering infections more treatable) and in preventing nosocomial infections from medical implants such as i.v. tubing, catheters, and other medical equipment.^427^ While research in antibody–antibiotic conjugates is limited, they appear to have significant promise as antibacterial agents.

### 5.2 ADCs as Immunomodulatory Agents

Glucocorticoids are efficient anti-inflammatory drugs, yet their use is dose-limited by systemic toxicity, causing severe side effects such as immunosuppression and metabolic disorders. It has been suggested that applying the ADC strategy developed for oncological malignancies may provide a solution for avoiding glucocorticoid toxicity.

Glucocorticoids exert their anti-inflammatory effects by suppressing the release of tumor-necrosis factor-α (TNF-α) and other cytokines by macrophages.^22^ Thus, an anti-CD163 dexamethasone conjugate that selectively delivers the glucocorticoid to macrophages has been designed and tested. It elicited reduced TNF-α secretion *in vitro* and was 50-fold more active *in vivo* than the nonconjugated dexamethasone in animal models.^428−431^ Another glucocorticoid, a fluticasone propionate analog, was conjugated onto an anti-CD74 mAb targeting B-cells and was reported to exhibit immuno-suppressive activity in human B cells.^173^ A glucocorticoid-based ADC, ABBV-3373, has been developed by conjugation of a proprietary dexamethasone derivative on the anti-TNF-α adalimumab, against autoimmune disease^432,433^ and specifically rheumatoid arthritis.^434^ The glucocorticoid released after cell internalization and lysosomal escape activates the glucocorticoid receptor pathway and provokes an anti-inflammatory cascade in nucleus.^434^ The data indicate that anti-TNF ADC delivering a glucocorticoid receptor modulator (GRM) payload into activated immune cells may provide enhanced efficacy against immune mediated diseases while minimizing systemic adverse effects associated with standard glucocorticoid treatment.

Table 2 provides some examples of ADCs tested for nononcological indications, with their targets and payloads.^22,131,413−417^

**Table 2:** Examples of ADC Strategy Tested to Modulate Pathogenic Cellular Activity in Non-Oncology Indications^a^

Target	Payload	Payload class	Disease	Ref
E-selectin (CD62E)	dexamethasone	GRM	inflammatory disorders	(435)
CD163	dexamethasone	GRM	inflammatory disorders	(428)
CD70	budesonide	GRM	immune diseases	(436)
CD74	fluticasone propionate	GRM	autoimmune diseases, systemic lupus erythematosus	(173)
Prolactin Receptor (PRLR)	glucocorticoid	GRM	undisclosed	(437)
TNFα	proprietary dexamethasone derivative	GRM	autoimmune disease, rheumatoid arthritis	(432−434)
CD71	siRNA	siRNA	muscular diseases	(438)
CD19, B220	siRNA	siRNA	myasthenia gravis	(439)
*S. aureus*	rifampicin analog	antibiotic	infectious disease	(440, 441)
β-GlcNAc WTA				
Chemokine receptor CXCR4	dasatinib	kinase Inhibitor	hematological disorders	(174)
Leukocyte integrin CD11a	LXR agonist	LXR agonist	atherosclerosis, inflammation	(442)
Leukocyte integrin CD11a	GSK256066	PDE4 Inhibitor	chronic inflammation	(175)
IL-6 receptor	alendronate	bisphosphonate	rheumatoid arthritis	(443)
CD30	monomethyl auristatin E (MMAE)	microtubule Inhibitor	steroid-refractory acute graft-vs-host disease; severe active diffuse cutaneous systemic sclerosis	(444, 445)

a Abbreviations: GRM, Glucocorticoid Receptor Modulator; LXR, Liver X Receptor; PDE4, Phosphodiesterase 4; TNFα, Tumor Necrosis Factor α.

### 5.3 ADCs across Different Indications

Aiming at neurodegenerative diseases such as Alzheimer’s and Parkinson’s diseases is complicated by the existence of the blood–brain barrier (BBB). To augment brain delivery of antibody therapeutics, endogenous macromolecule transportation pathways such as receptor-mediated transcytosis and carrier-mediated transport have been explored recently.^446^ Invasive strategies, such as ultrasound, microbubbles, and direct injection into the brain (e.g., intracerebroventricular delivery), have also been used to deliver ADC across the blood-brain barrier.^447,448^

## 6 Private Investment

**Figure 19:**
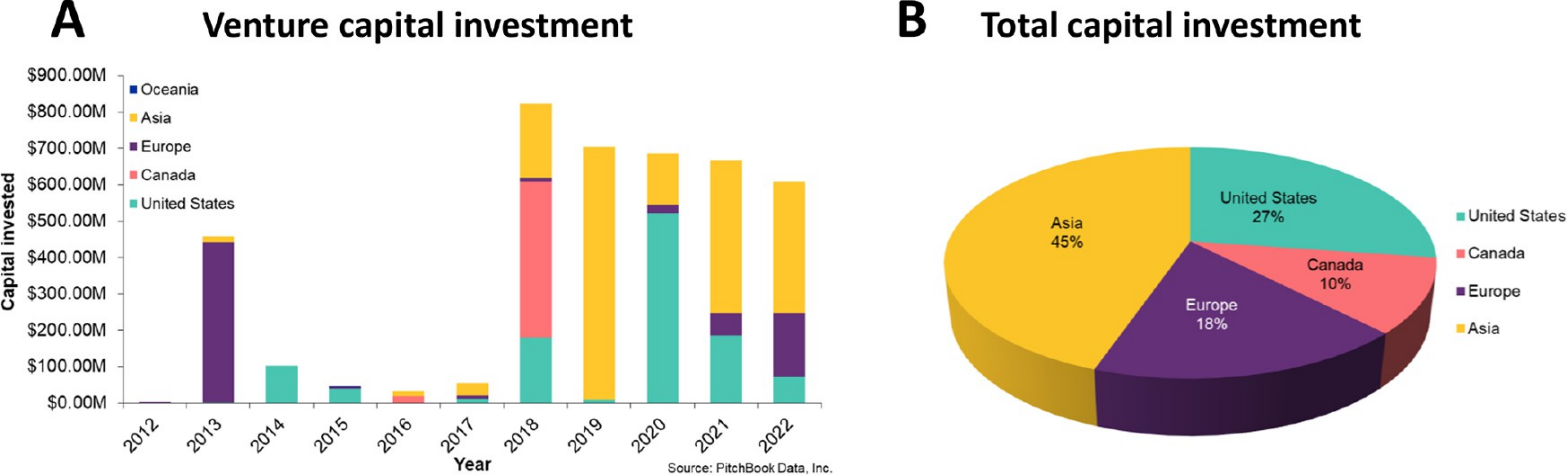
Capital invested by global region for the period 2012–2022 in the antibody–drug conjugate field: (A) Venture capital investment. (B) Total capital investments.

Analyzing the collective international private investments in the ADC sector offers a valuable understanding of the business fascination with the commercial potential of this domain. Conducting a search for ADCs on PitchBook,^449^ an online platform for investment data, reveals comprehensive capital undertakings in this field. The search revealed that capital investments showed a dramatic and substantial increase after 2017 in this field (Figure 19A). Subsequent years show more or less sustained interest in ADCs with the amount of capital invested being > $600 M (Figure 19A). A breakdown across global regions reveals that the lion’s share of investments are from Asia, followed by United States with Europe and Canada making up the rest (Figure 19B). The venture capital investment data in this area clearly shows significant recent interest in ADCs, endorsing their therapeutic and commercial potential.

## 7 ADC Clinical Development

### 7.1 Preclinical Development

**Figure 20:**
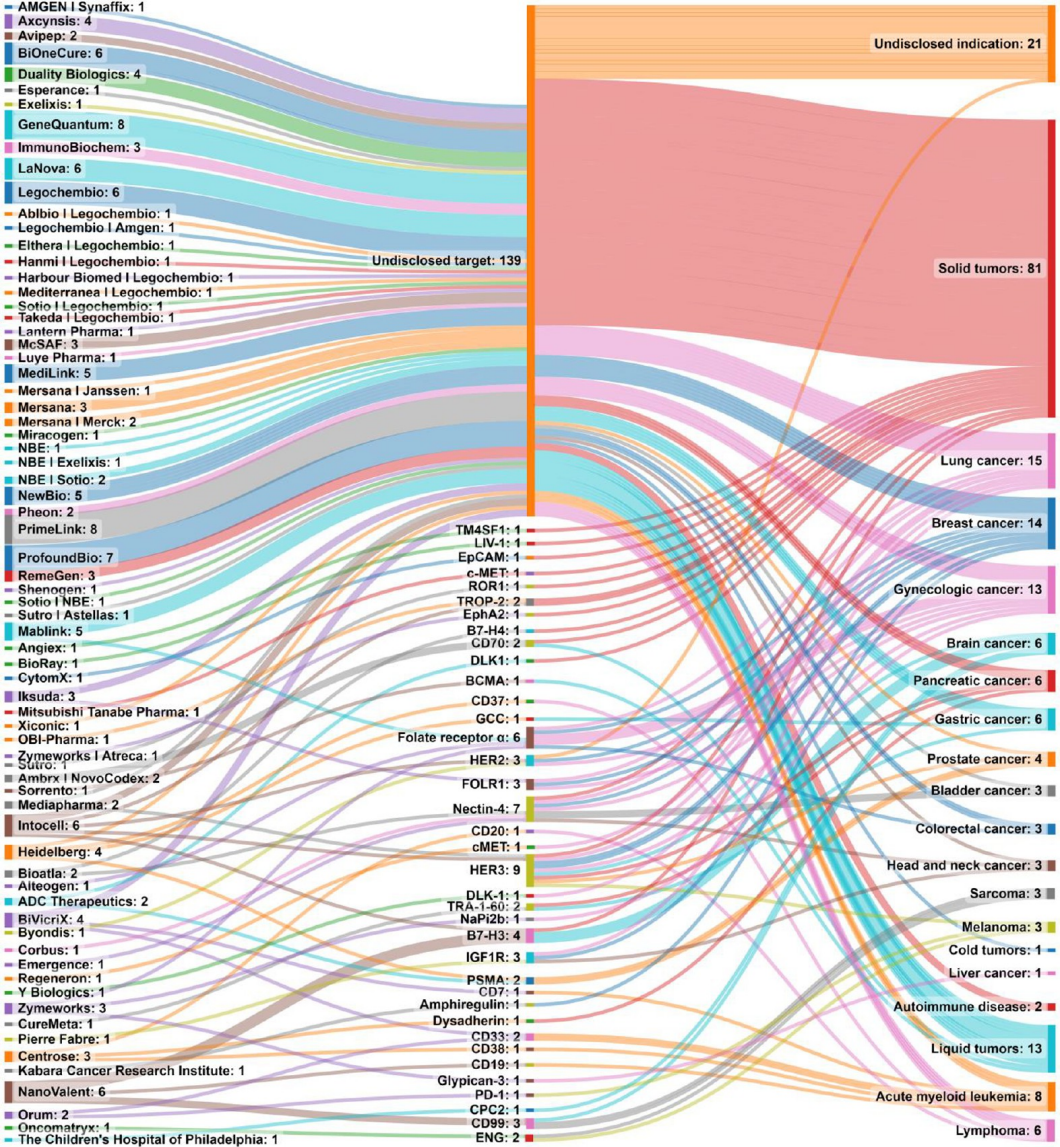
Organizations conducting preclinical ADC research with the number of ADC candidates in their pipeline (left), target antigen (middle), and disease indication (right).

Examination of preclinical ADC development reveals that over 50 worldwide organizations, mainly from the United States, Europe, and Asia, are currently conducting research on over 160 ADC preclinical candidates (Supporting Information, Table S1). Table S1 reveals organizations conducting preclinical ADC research, their ADC drug candidates, and target antigens, along with target indications. Figure 20 is a visual representation of these organizations and their number of ADC candidate drugs, target antigens, and disease indications. A few of these companies have disclosed the target antigens for their ADC. Antigens HER3, Nectin-4, Folate receptor α, B7H3, CD99, and IGF1R are leading the way with the most ADC drug candidates utilizings these targets for disease treatment. Of disclosed preclinical indications ∼50% are categorized into the broader solid tumors and hematological malignancy indication with the other 50% attributed to more specific indications. Solid tumor research is heavily favored currently for preclinical development with the largest number of ADC drug candidates focused on this indication. Disclosed solid tumors with the largest research focus include, lung, breast, gynecologic, brain, pancreatic, and gastric cancers. In general, hematological malignancies appear to have lesser research efforts being directed toward them, with acute myeloid leukemia and lymphoma being the exceptions. Outside of oncology, Duality Biologics is performing preclinical research for the use of ADCs in the treatment of autoimmune disease.

### 7.2 Clinical Development

**Figure 21:**
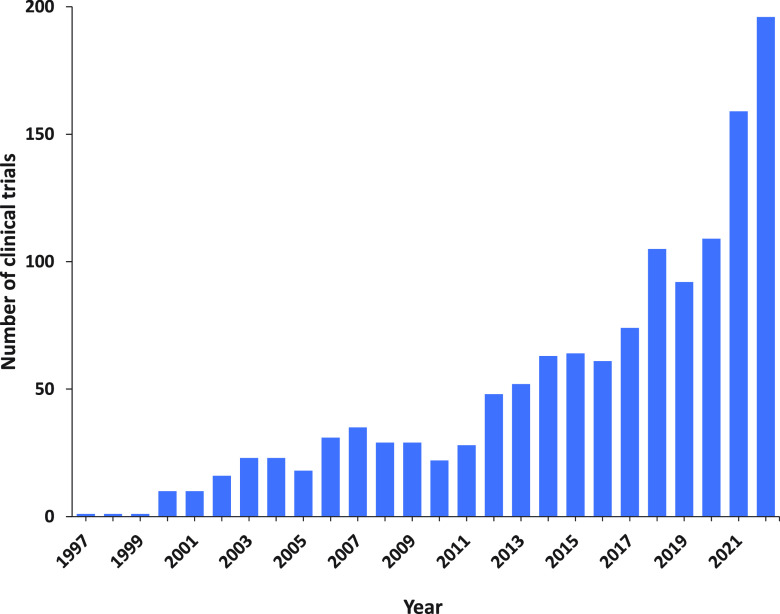
Number of ADC clinical trials by year.

A representative selection of ADC clinical trials has been examined within this section to gain an overall view of the past, present, and future states of clinical development. A selection of approximately 1,500 ADC clinical trials from https://clinicaltrials.gov are examined against time, clinical trial phase, status, and disease indications. These trials reveal that ADCs are slowly growing in clinical development, with Figure 21 showing an oscillating curve starting in 1997 with more consistent and steady growth starting around 2012 and continuing beyond.

**Figure 22:**
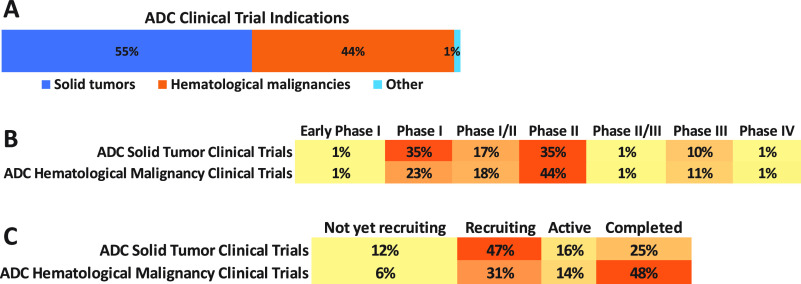
(A) ADC clinical trial indications; (B) Percentage of ADC clinical trials in various phases for the treatment of solid tumors and hematological malignancies; (C) Percentage of ADC clinical trials in various statuses for the treatment of solid tumors and hematological malignancies.

Examination of all ADC clinical trials against their indications further revealed that nearly all clinical trials target the treatment of both solid tumors and hematological malignancies. ADC clinical trials targeting solid tumors make up 55% of all ADC clinical trials, with hematological malignancies making up 44%. Only 1% of all ADC clinical trials target a disease beyond oncology (Figure 22A). With ADCs historically targeting cancers, much room is available for expansion into other disease treatment indications, such as autoimmune diseases, rheumatoid arthritis and diffuse cutaneous systemic sclerosis, which are in current active clinical trials.^450,451^

Analysis of ADC clinical trial phases reveals that most oncology clinical trials are in early phase development. Solid tumors have 88% of their trials in early stage clinical trials from early Phase I trials through Phase II trials with hematological malignancies encompassing 86% (Figure 22B). Examining these clinical trials, a step further, by status, shows ADC clinical trials treating solid tumors have a higher percentage of trials in the not yet recruiting, recruiting, and active statuses than trials treating hematological malignancies. 75% of current clinical trials for solid tumor indications are currently active or getting ready to be active in the clinical trial pipeline (Figure 22C) versus 51% for hematological malignancy indications. While ADC clinical trials focused on oncology are largely equal when it comes to phases of study, currently solid tumor indications have a more active presence in the clinical trial pipeline over hematological malignancy indications.

**Figure 23:**
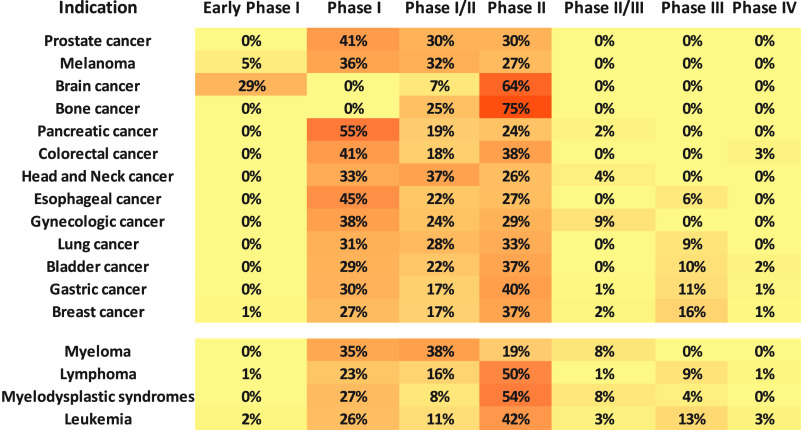
Percentage of ADC clinical trials in various phases for the treatment of specific solid tumors and hematological malignancies.

**Figure 24:**
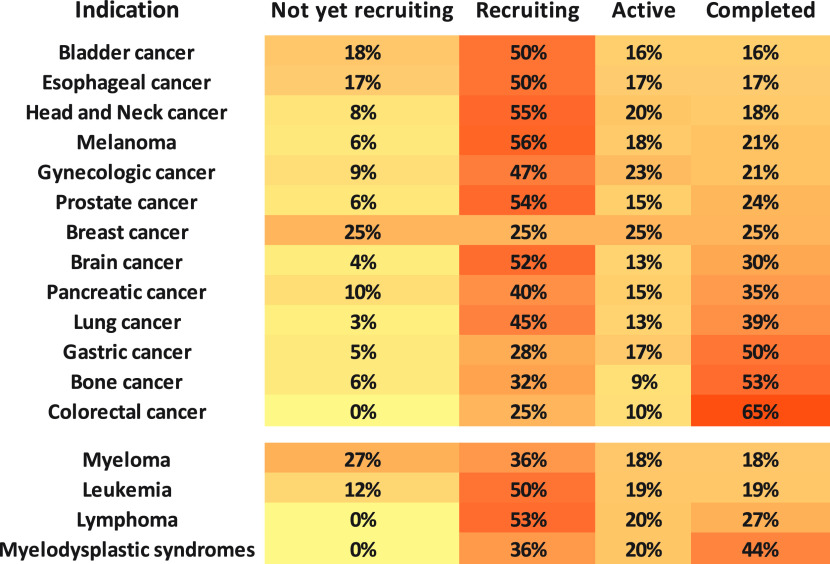
Percentage of ADC clinical trials in various statuses for the treatment of specific solid tumors and hematological malignancies.

Clinical trials that disclose a specific tumor indication are characterized by the trial phase and stage in Figures 23 and 24. Prostate, melanoma, brain, bone, and pancreatic cancer are the solid tumor indications with the largest percentage of trials in early stage clinical development from early Phase I trials through Phase II trials. On the other hand, breast, gastric, bladder, and lung cancer are more established in the clinical pipeline with the highest percentage of late-stage trials. In respect to hematological malignancies, myeloma has the largest percentage of clinical trials in early phase development with leukemia and lymphoma having the largest percentage of late-stage trials.

Examining the above clinical trials, by status, shows ADC clinical trials treating bladder, esophageal, head and neck, melanoma, and gynecologic cancer have the highest percentage of trials in the not yet recruiting, recruiting, and active statuses for solid tumor indications. 79–84% of current clinical trials for solid tumor indications are currently active or getting ready to be active in the clinical trial pipeline (Figure 23). Pancreatic, lung, gastric, bone, and colorectal cancer have the greatest percentage of completed trials ranging from 35 to 65%, respectively. The hematological malignancy indication myeloma has the highest percentage of trials (81%) in the not yet recruiting, recruiting, and active statuses contrasting with myelodysplastic syndrome, which has 44% of its trials in the completed status.

A selection of ADC clinical trials focusing on the treatment of solid tumors is highlighted in Table 3 to display the variety of ADC candidates in clinical development along with their sponsors, targeted solid tumor indications, target antigen, and phases. Only clinical trials currently in recruiting or active status are showcased to reveal the most current and promising ADC candidates. Over 135 ADC candidates are currently in clinical development for solid tumor indications (Supporting Information, Table S2). ADC candidates focusing on the treatment of lung, gastric, pancreatic, colorectal, breast, gynecologic, prostate, and bladder cancers, along with head and neck cancers, are all currently highly represented in the clinical pipeline (Table 3). Bispecific ADC candidates are also included as they have entered Phase I and II clinical trials for the treatment of various solid tumors including breast, lung, and esophageal cancer.

**Table 3:** Highlighted ADC Clinical Trials with Solid Tumor Indications in the Development Pipeline

ADC intervention	Sponsor	Solid tumor indications	Target	NCT number	Clinical trial phase
ABBV-400	AbbVie	Non-Small Cell Lung Cancer|Gastresophageal Cancer|Colorectal Cancer	cMET	NCT05029882	Phase I
ADCT-901	ADC Therapeutics	Advanced Solid Tumors	KAAG1	NCT04972981	Phase I
ARX517	Ambrx	Advanced Solid Tumors	PSMA	NCT04662580	Phase I
ASN004	Kirilys Therapeutics	Solid Tumors	5T4	NCT04410224	Phase I
AZD9592	AstraZeneca	Non-Small Cell Lung Cancer|Head and Neck Cancer	cMET l EGFR(bispecific)	NCT05647122	Phase I
BAY-2315497	Bayer	Prostate Cancer	PSMA	NCT03724747	Phase I
BYON3521	Byondis	Solid Tumors	cMET	NCT05323045	Phase I
CMG901	Keymed Biosciences Co.	Gastric Cancer l Pancreatic Cancer	Claudin 18.2	NCT04805307	Phase I
DS-6000a	Daiichi Sankyo	Renal Cell Carcinoma| Gynecologic Cancer	CDH6	NCT04707248	Phase I
HS-20093	Shanghai Hansoh Biomedical Co.	Advanced Solid Tumors	B7H3	NCT05276609	Phase I
IBI-343	Innovent Biologics	Advanced Solid Tumors	Claudin 18.2	NCT05458219	Phase I
IMGN151	ImmunoGen	Gynecologic Cancer	FRα	NCT05527184	Phase I
M1231	EMD Serono Research and Development Institute	Esophageal Cancer| Non-Small Cell Lung Cancer	MUC1-EGFR (bispecific)	NCT04695847	Phase I
MYTX-011	Mythic Therapeutics	Lung Cancer	cMET	NCT05652868	Phase I
ORM-5029	Orum Therapeutics	HER2-positive Breast Cancer	HER2	NCT05511844	Phase I
PYX-201	Pyxis Oncology	Advanced Solid Tumors	Extradomain B fibronectin	NCT05720117	Phase I
STRO-002	Sutro Biopharma	Gynecologic Cancer	CD74	NCT03748186	Phase I
TORL-1–23	TORL Biotherapeutics	Advanced Solid Tumor| Gynecologic Cancer| Lung Cancer	Claudin 18.2	NCT05103683	Phase I
XMT-1660	Mersana Therapeutics	Breast Cancer| Gynecologic Cancer	B7H4	NCT05377996	Phase I
YL202	MediLink Therapeutics	Non-Small Cell Lung Cancer| Breast Cancer	HER3	NCT05653752	Phase I
Zanidatamab Zovodotin	Zymeworks	HER2-expressing Cancers	HER2 domain II l HER2 domain IV (Bispecific)	NCT03821233	Phase I
9MW2821	Mabwell	Solid Tumors	Nectin-4	NCT05216965	Phase I |Phase II
AZD8205	AstraZeneca	Breast Cancer|Biliary Tract Carcinoma| Gynecologic Cancer	B7H4	NCT05123482	Phase I |Phase II
BB-1705	Bliss Biopharmaceutical (Hangzhou) Co.	Solid Tumor	EGFR	NCT05217693	Phase I |Phase II
BDC-1001	Bolt Biotherapeutics	HER2 Positive Solid Tumors	HER2	NCT04278144	Phase I |Phase II
BIO-106	BiOneCure Therapeutics	Advanced Solid Tumor	TROP2	NCT05320588	Phase I |Phase II
DB-1303	DualityBio	HER2 Positive Advanced Solid Tumor	HER2	NCT05150691	Phase I |Phase II
FOR46	Fortis Therapeutics	Prostate Cancer	CD46	NCT05011188	Phase I |Phase II
LM-302	Turning Point Therapeutics	Advanced Solid Tumor	Claudin 18.2	NCT05001516	Phase I |Phase II
MRG004A	Shanghai Miracogen	Advanced or Metastatic Solid Tumors	Tissue Factor	NCT04843709	Phase I |Phase II
NBE-002	NBE-Therapeutics	Breast Cancer	ROR1	NCT04441099	Phase I |Phase II
OBI-999	OBI Pharma	Advanced Solid Tumor	Globo H	NCT04084366	Phase I |Phase II
Ozuriftamab Vedotin	BioAtla	Non-Small Cell Lung Cancer| Triple Negative Breast Cancer| Melanoma| Head and Neck Cancer	ROR2	NCT03504488	Phase I |Phase II
PRO1184	ProfoundBio	Gynecologic Cancer| Lung Cancer | Breast Cancer	FRα	NCT05579366	Phase I |Phase II
REGN5093	Regeneron Pharmaceuticals	NSCLC	cMET l CMET (Bispecific)	NCT04077099	Phase I |Phase II
SKB264	Klus Pharma	Gynecologic Cancer| Gastric Cancer| Bladder Cancer| Lung Cancer| Head and Neck Cancer| Breast Cancer	TROP2	NCT04152499	Phase I |Phase II
SOT102	SOTIO Biotech	Gastric Cancer| Pancreatic Cancer	Claudin 18.2	NCT05525286	Phase I |Phase II
W0101	Pierre Fabre	Advanced or Metastatic Solid Tumors	IGF-1R	NCT03316638	Phase I |Phase II
Zilovertamab vedotin	Merck	Bladder Carcinoma	ROR1	NCT05562830	Phase I |Phase II
Mecbotamab Vedotin	BioAtla	Non-Small Cell Lung Cancer	AXL	NCT04681131	Phase II
CX-2009	CytomX Therapeutics	Breast Cancer	CD166	NCT04596150	Phase II
MORAb-202	Bristol-Myers Squibb	Non-Small Cell Lung Cancer	Fos-related antigen	NCT05577715	Phase II
RC108	RemeGen Co.	Gastric Cancer	cMET	NCT05628857	Phase II
Vobramitamab Duocarmazine	MacroGenics	Prostate Cancer	B7H3	NCT05551117	Phase II
ARX78	Jiangsu HengRui Medicine Co.	HER2-positive Breast Cancer	HER2	NCT05426486	Phase II |Phase III
Enfortumab Vedotin	Astellas Pharma	Bladder Cancer	Nectin-4	NCT03474107	Phase III
Mirvetuximab Soravtansine	ImmunoGen	Gynecologic Cancer	FRα	NCT04209855	Phase III
MRG002	Shanghai Miracogen	Advanced or Metastatic Urothelium Cancer	HER2	NCT05754853	Phase III
Patritumab Deruxtecan	Daiichi Sankyo	Nonsmall Cell Lung Cancer	HER3	NCT05338970	Phase III
RC48	RemeGen Co.	Bladder Cancer	HER	NCT05302284	Phase III
SAR-408701	Sanofi	Non-Small Cell Lung Cancer Metastatic	CEACAM5	NCT04154956	Phase III
Telisotuzumab Vedotin	AbbVie	Non-Small Cell Lung Cancer	cMET	NCT04928846	Phase III
Tisotumab Vedotin	Seagen	Gynecologic Cancer	Tissue Factor	NCT04697628	Phase III
Trastuzumab Emtansine	Hoffmann-La Roche	Breast Cancer	HER2	NCT01772472	Phase III
Trastuzumab Duocarmazine	Byondis	Metastatic Breast Cancer	HER2	NCT03262935	Phase III
Upifitimab Rilsodotin	Mersana Therapeutics	Gynecologic Cancer	NaPi2b	NCT05329545	Phase III
Sacituzumab Govitecan	Gilead Sciences	Solid Tumors	TROP2	NCT04319198	Phase IV

A more exhaustive list of ADC candidates in clinical development for all indications, along with their companies and target antigens, can be located in Table S2. The most utilized target antigen for ADCs currently in clinical trials is HER2, followed by TROP2, Claudin 18.2, cMET, and B7H3, respectively. Also, around 40 ADC candidates currently in clinical trials are topoisomerase I inhibitors which impact DNA replication in cancer cells, resulting in cancer cell death.

ADC clinical trials focusing on the treatment of hematological malignancies are highlighted in Table 4, similar to solid tumors. This table showcases the variety of ADC candidates currently being researched for the treatment of hematological malignancies along with their sponsor, hematological malignancy indication, target antigen, and phase. Clinical trials in recruiting or active status are highlighted. The total of ADC candidates currently in clinical development for hematological malignancies is less than solid tumors at around 30 ADC drug candidates compared to greater than 135 for solid tumors (Supporting Information, Table S2). ADC candidates focusing on the treatment of multiple myeloma and various types of leukemia and lymphoma are all currently being researched in the clinical development pipeline (Table 4).

**Table 4:** Highlighted ADC Clinical Trials with Hematological Malignancies Indications in the Development Pipeline

ADC intervention	Sponsor	Hematological malignancy indications	Target	Clinical trial phase	NCT number
INA03	INATHERYS	Acute Lymphoblastic Leukemia |Acute Myeloid Leukemia	CD71	Early Phase I	NCT03957915
ABBV-319	AbbVie	Diffuse Large B-Cell Lymphoma|Lymphocytic Leukemia|Follicular Lymphoma	CD19	Phase I	NCT05512390
CC-99712	Celgene	Multiple Myeloma	BCMA	Phase I	NCT04036461
CS5001	CStone Pharmaceuticals	Advanced Lymphoma	PTK7	Phase I	NCT05279300
F0002	Shanghai Fudan-Zhangjiang Bio-Pharmaceutical Co.	CD30+ hematological malignancies	CD30	Phase I	NCT03894150
JBH492	Novartis	Non-Hodgkin’s Lymphoma|Lymphocytic Leukemia	CCR7	Phase I	NCT04240704
Moxetumomab Pasudotox	National Cancer Institute	Hairy Cell Leukemia	CD22	Phase I	NCT03805932
MRG001	Shanghai Miracogen	B-cell Non-Hodgkin Lymphoma	CD20	Phase I	NCT05155839
TRS005	Zhejiang Teruisi Pharmaceutical	B-cell Non-Hodgkin’s Lymphoma	CD20	Phase I	NCT05395533
ADCT-602	ADC Therapeutics	Acute Lymphoblastic Leukemia	CD22	Phase I |Phase II	NCT03698552
BN301	BioNova Pharmaceuticals (Shanghai)	Non Hodgkin’s Lymphoma|Diffuse Large B Cell Lymphoma|Follicular Lymphoma	CD74	Phase I |Phase II	NCT05611853
CX-2029	CytomX Therapeutics	Diffuse Large B Cell Lymphoma	CD71	Phase I |Phase II	NCT03543813
HDP-101	Heidelberg Pharma	Multiple Myeloma|Plasma Cell Disorder	BCMA	Phase I |Phase II	NCT04879043
PRO1160	ProfoundBio	Non Hodgkin’s Lymphoma	CD70	Phase I |Phase II	NCT05721222
STI-6129	Sorrento Therapeutics	Multiple Myeloma	CD38	Phase I |Phase II	NCT05308225
Trastuzumab Emtansine	National Cancer Institute	Advanced Lymphoma|Refractory Plasma Cell Myeloma	HER2	Phase II	NCT04439110
Zilovertamab vedotin	Merck	Diffuse Large B-Cell Lymphoma	ROR1	Phase II |Phase III	NCT05139017
Belantamab mafodotin	GlaxoSmithKline	Multiple Myeloma	BCMA	Phase III	NCT04162210
Gemtuzumab Ozogamicin	Gruppo Italiano Malattie EMatologiche dell’Adulto	Acute Myeloid Leukemia	CD33	Phase III	NCT04168502
Loncastuximab Tesirine	ADC Therapeutics	Diffuse Large B-Cell Lymphoma	CD19	Phase III	NCT04384484
Polatuzumab Vedotin	Hoffmann-La Roche	Diffuse Large B-Cell Lymphoma	CD79	Phase III	NCT03274492
Brentuximab vedotin	Takeda	Anaplastic Large-cell Lymphoma	CD30	Phase IV	NCT01909934
Inotuzumab ozogamicin	Pfizer	Leukemia| Precursor B-Cell Lymphoblastic Leukemia-Lymphoma	CD22	Phase IV	NCT03677596

While most ADC clinical trials focus on oncology, there are a few studies exploring research in emerging areas of interest. These include trials looking at the treatment of autoimmune disorders diffuse cutaneous systemic sclerosis and rheumatoid arthritis, along with amyloidosis (Table 5).

**Table 5:** Highlighted ADC Clinical Trials with Non-Oncology Indications in the Development Pipeline

ADC intervention	Sponsor	Indications	Target	Clinical trial phase	NCT number
Brentuximab vedotin	Seagen	Diffuse Cutaneous Systemic Sclerosis	CD30	Phase I |Phase II	NCT03222492
STI-6129	Sorrento Therapeutics	Light Chain Amyloidosis	CD38	Phase I |Phase II	NCT04316442
ABBV-154	AbbVie	Polymyalgia Rheumatica	TNF	Phase II	NCT04972968
ABBV-154	AbbVie	Rheumatoid Arthritis	TNF	Phase II	NCT04888585
Belantamab mafodotin	GlaxoSmithKline	Amyloidosis	BCMA	Phase II	NCT04617925

### 7.3 Approved ADCs

Currently, there are 15 approved ADCs that have received regulatory approval anywhere in the world (Table 6).

**Table 6:** List of Approved ADCs,^21,115,452−456^ with the Number of Related Documents in the CAS Content Collection^b^

Drug	Trade name	CAS REG #	Maker	Condition	Target	Approval year	mAb	Linker	Payload	Payload action	DAR	Conjugation	No. documents
Gemtuzumab ozogamicin^51,52^	Mylotarg	220578–59–6	Pfizer/Wyeth	relapsed acute myelogenous leukemia	CD33	2000; 2017	humanized IgG4k	hydrazone, acid cleavable	N-acetyl-γ-calicheamicin (ozogamicin)	DNA cleavage	2–3	Lys	2066
Brentuximab vedotin^53,54^	Adcetris	914088–09–8	Seagen Genetics, Millennium/Takeda	relapsed Hodgkin lymphoma, relapsed anaplastic large cell lymphoma	CD30	2011	chimeric IgG1	Val-Cit, enzyme cleavable	MMAE/Auristatin	microtubule Inhibitor	4	Cys	1738
Trastuzumab emtansine^50,55^	Kadcyla	1018448–65–1	Genentech, Roche	metastatic HER2-positive breast cancer	HER2	2013	humanized IgG1	MCC, noncleavable	DM1/Maytansinoid	microtubule Inhibitor	3.5	Lys	1507
Inotuzumab ozogamicin^48,56^	Besponsa	635715–01–4	Pfizer/Wyeth	CD22-positive acute lympho-blastic leukemia	CD22	2017	humanized IgG4	hydrazone, acid cleavable	N-acetyl-γ-calicheamicin (ozogamicin)	DNA cleavage	6	Lys	518
Moxetumomab pasudotox^57,58^	Lumoxiti	1020748–57–5	AstraZeneca	relapsed or refractory hairy cell leukemia	CD22	2018	-	mc-VC-PABC enzyme cleavable	Pseudomonas Exotoxin A (PE38)	peptide toxin class	-	Cys	172
Polatuzumab vedotin-piiq^59,60^	Polivy	1313206–42–6	Genentech, Roche	diffuse large B-cell lymphoma	CD79	2019	humanized IgG1	Val–Cit, enzyme cleavable	MMAE/Auristatin	microtubule Inhibitor	3.5	Cys	200
Enfortumab vedotin^61,62^	Padcev	1346452–25–2	Astellas/Seagen Genetics	locally advanced or metastatic urothelial cancer	Nectin-4	2019	humanized IgG1	Val-Cit, enzyme cleavable	MMAE/Auristatin	microtubule Inhibitor	3.8	Cys	179
Trastuzumab deruxtecan^63,64^	Enhertu	1826843–81–5	AstraZeneca/Daiichi Sankyo	unresectable or metastatic HER2-positive breast cancer	HER2	2019	humanized IgG1	maleimide—GGFG enzyme cleavable	DXd/Camptothecin	TOPO1 Inhibitor	8	Cys	291
Sacituzumab govitecan^65,66^	Trodelvy	1491917–83–9	Immuno-medics	metastatic triple-negative breast cancer	Trop-2	2020	humanized IgG1	CL2A acid cleavable	SN-38/Camptothecin	TOPO1 Inhibitor	7.6	Cys	237
Belantamab mafodotin-blmf^67,68^	Blenrep	2050232–20–5	GlaxoSmithKline (GSK)	relapsed or refractory multiple myeloma	BCMA	2020^a^	humanized IgG1	MC noncleavable	MMAF/Auristatin	microtubule Inhibitor	4	Cys	132
Loncastuximab tesirine-lpyl^69,70^	Zynlonta	1879918–31–6	ADC Therapeutics	large B-cell lymphoma	CD19	2021	IgG1	enzyme cleavable	SG3199/PBD dimer	DNA cleavage	2.3	Cys	66
Tisotumab vedotin-tftv^71,72^	Tivdak	1418731–10–8	Seagen Inc.	recurrent or metastatic cervical cancer	Tissue factor	2021	IgG1	enzyme cleavable	MMAE/Auristatin	microtubule Inhibitor	4	Cys	55
Cetuximab Sarotalocan^21,73,74^	Akalux	2166339–33–7	Rakuten Medical	unresectable locally advanced, recurrent head and neck cancer	EGFR	2021	IgG1	N/A	IRDye700DX	photosensitizer	1.3–3.8	Lys	2
Disitamab Vedotin^75,76^	Aidixi	2136633–23–1	RemeGen	HER2-overexpressing gastric cancer	HER2	2021	IgG1	enzyme cleavable	MMAE	microtubule Inhibitor	4	Cys	15
Mirvetuximab soravtansine^77,78^	Elahere	1453084–37–1	ImmunoGen	platinum-resistant ovarian cancer	FRα	2022	IgG1	enzyme cleavable	DM4	microtubule inhibitor	3.4	Cys	87

a Withdrawn on Nov 22, 2022.^79^

b Abbreviations: B cell maturation antigen (BCMA); cluster of differentiation (CD); cleavable PEG8- and triazole-containing PABC–peptide– MC linker (CL2A); derivative of maytansine (DM1); exatecan derivative (DXd); glycine–glycine–phenylalanine–glycine tetrapeptide linker (GGFG); human epidermal growth factor receptor 2 (HER2); maleimidocaproyl (MC); 4-maleimidomethyl cyclohexane-1-carboxylate (MCC); monomethyl auristatin E (MMAE); monomethyl auristatin F (MMAF); active metabolite of the topoisomerase I inhibitor irinotecan (SN-38); tumor-associated calcium signal transducer 2 (TROP-2).

#### 7.3.1 Hematological Malignancies

- Gemtuzumab ozogamicin (Mylotarg) contains an anti-CD33 humanized IgG4κ monoclonal antibody connected to ozogamicin, a calicheamicin derivative payload, via a cleavable hydrazone linker.^457^ It binds preferentially to cells expressing the CD33 surface antigen, leading to the internalization of the gemtuzumab ozogamicin ADC and cleavage of the linker within the lysosomes via acid hydrolysis, with the subsequent calicheamicin reduction by glutathione inducing double-strand DNA breaks, resulting in cell death.^458,459^ Gemtuzumab ozogamicin was the first ADC to reach the clinic, approved by the FDA in 2000 for the treatment of relapsed or refractory CD33-positive acute myeloid leukemia (AML).^460^ The accelerated approval required postmarketing trials to be conducted to confirm treatment efficacy. The negative results from those studies (NCT00085709; ISRCTN17161961) prompted Pfizer to withdraw gemtuzumab ozogamicin from the market in 2010.^461−463^ Later on, based on the positive results of subsequent trials (NCT00927498; NCT00091234), using reduced dosing strategies, gemtuzumab ozogamicin was reapproved by the USFDA in 2017 for treatment of newly diagnosed CD33-positive AML as well as for relapsed or refractory CD33-positive AML.^127,463,464^
- Brentuximab vedotin (Adcetris) was the second ADC to receive accelerated US FDA approval in 2011 based on Phase II trials in patients with relapsed Hodgkin’s lymphoma or systemic anaplastic large cell lymphoma.^43,465,466^ It comprises MMAE conjugated to an anti-CD30 antibody via an enzyme cleavable Val-Cit linker.^44,467^ Hodgkin’s lymphoma cells, as well as malignant cells of anaplastic large cell lymphoma express high levels of CD30.^44^ In 2015, brentuximab vedotin received full approval from the US FDA based on the results of the Phase III trial.^468^ A recently reported randomized Phase III trial provides compelling evidence in favor of brentuximab vedotin for treating cutaneous T cell lymphoma.^469^ The benefit of brentuximab vedotin has been examined in randomized studies in combination with approved chemotherapeutic agents (NCT01712490; NCT01777152), as well as in combination with immune checkpoint inhibitors (NCT02684292; NCT03138499).^44,88^
- Inotuzumab ozogamicin (Besponsa) is the second ADC using the calicheamicin ozogamicin payload, linked to a humanized mAb targeting the B cell antigen CD22, with an average DAR of 5–7. CD22 is a cell surface antigen in the majority of B-cell acute lymphoblastic leukemia.^470,471^ The safety and efficacy of inotuzumab ozogamicin was evaluated in an open-label, randomized, international, multicenter Phase III study (INO-VATE 1022).^472^ It was approved by the US FDA in 2017 against relapsed or refractory acute lymphoblastic leukemia.^127,473,474^
- Moxetumomab pasudotox (Lumoxiti) consists of moxetumomab targeting CD22 conjugated to a 38kD fragment of Pseudomonas exotoxin A (PE38).^58^ CD22 is expressed on mature B cells and to a larger extent on 100% of hairy cells, which provides an ideal therapeutic target for the treatment of hairy cell leukemia, a rare hematological malignancy, characterized by splenomegaly, hemorrhage, and an accumulation of abnormal B lymphocytes.^475−477^ Upon binding to CD22, moxetumomab pasudotox is internalized, its mc-VC-PABC linker cleaved by proteases and the catalytic domain of the exotoxin is released inside cancer cells leading to inhibition of translation of proteins and apoptosis. US FDA approved Lumoxiti of AstraZeneca in 2018 for the treatment of patients with hairy cell leukemia who have previously failed to receive at least two systemic therapies (including purine nucleoside analogs).^478^ Moxetumomab pasudotox was thus the first new drug approved for the treatment of hairy cell leukemia in the past 20 years.^88^
- Polatuzumab vedotin (Polivy) contains a humanized antibody targeting CD79b antigen, linked to microtubule-disrupting MMAE payload via a protease-cleavable dipeptide linker (mc-VC-PABC) with an average DAR of 3.5.^479^ CD79b is expressed on >90% of B-cell non-Hodgkin lymphomas and has proven to be a promising antibody target.^480,481^ Polatuzumab vedotin selectively binds to CD79b upon administration, followed by endocytosis and proteolytic cleavage to release MMAE inducing cell cycle arrest and subsequent cell death. Polivy was approved by the US FDA in 2019, for use in treatment of diffuse large B-cell lymphoma, the most common type of non-Hodgkin lymphomas, in patients who have received at least two prior therapies.^482^
- Belantamab mafodotin (Blenrep) is composed of a humanized anti-B cell maturation antigen (BCMA) mAb coupled with cytotoxic agent MMAF, a mitotic inhibitor, through a noncleavable maleimidocaproyl (MC) linker. Belantamab mafodotin has an average DAR of 4. BCMA is a transmembrane glycoprotein explicitly overexpressed on the surface of multiple myeloma cells.^483^ Upon internalization and degradation in lysosomes belantamab mafodotin releases MMAF inside multiple myeloma cells, which inhibits cell division by blocking microtubule polymerization, resulting in cell cycle arrest and inducing caspase-3-dependent apoptosis. As a result, belantamab mafodotin is effective at killing cancer cells overexpressing BCMA. The US FDA approved Blenrep in 2020 for the treatment of multiple myeloma, based on the results of the DREAMM-2 clinical trial.^484^
- Loncastuximab tesirine (Zynlonta) comprises a humanized mAb targeting CD19 conjugated to pyrrolobenzodiazepine (PBD) dimer via a cleavable (valine-alanine dipeptide) maleimide type linker, with an average DAR of ∼2.3.^485,486^ The PBD dimer is a novel generation of cytotoxic payload for ADC development.^152^ It binds to DNA and causes strong cross-linking that prevents DNA strand separation, thus preventing DNA transcription and replication and killing the cell.^487^ Zynlonta received accelerated approval by the US FDA in 2021, for the treatment of patients with large B-cell lymphoma after two or more lines of systemic therapy. The approval of Zynlonta was based on data from the LOTIS-2 trial.^488^

#### 7.3.2 Solid Tumors

- Trastuzumab emtansine (Kadcyla) includes an anti-HER2 humanized IgG1 monoclonal antibody connected to a DM1 payload via a noncleavable MCC linker. Because of the noncleavable linker present in trastuzumab emtansine, after entry into the HER2-positive cancer cell, mAb proteolysis inside lysosomes is necessary to release the free DM1 payload.^266,489^ Upon its release from the lysosome, DM1 binds to tubulin at the vinca-binding site and inhibits tubulin polymerization, inducing mitotic arrest and cell death.^490^ Trastuzumab emtansine was approved by the US FDA in 2013 as a single-agent treatment for HER2-positive metastatic breast cancer in patients previously administered trastuzumab and a taxane, either separately or in combination.^491^ In 2019, this was extended to include HER2- positive early breast cancer in patients with residual invasive disease after neoadjuvant taxane-based chemotherapy and trastuzumab-based treatment.^127,492^
- Enfortumab vedotin (Padcev) is approved by the US FDA for the treatment of patients with locally advanced or metastatic urothelial cancer.^493^ It comprises a fully human antinectin-4 IgG1κ monoclonal antibody (AGS-22C3), linked to MMAE via a protease-cleavable linker (MC-VC-PABC), with an average DAR of ∼3.8.^494^ Nectin-4 is a transmembrane protein, which is abundantly expressed in several malignancies, especially in urothelial carcinoma, thus being a compelling target for ADC molecular design. An accelerated approval was granted by the FDA in 2019, and a regular approval was further granted in 2021 based on results from an open-label, randomized, multicenter Phase III study (EV-301).^495−497^
- Enfortumab vedotin-ejfv (Padcev, Astellas Pharma) is a member of the first approved ADC-based combination therapy formulation: on April 3, 2023, the US FDA granted accelerated approval to enfortumab vedotin-ejfv with pembrolizumab (Keytruda, Merck) for treatment of locally advanced or metastatic urothelial carcinoma in patients ineligible for cisplatin-containing chemotherapy.^80^

- Trastuzumab deruxtecan (Enhertu) is a HER2-targeted ADC for the treatment of patients with unresectable or metastatic HER2-positive breast cancer who have received two or more prior anti-HER2 based regimens in the metastatic setting.^498^ It includes a humanized HER2 antibody (trastuzumab) conjugated to a topoisomerase I inhibitor (DXd) as a payload through an enzymatically cleavable tetrapeptide-based linker with an average DAR of 7–8. DXd is a very powerful payload,^499^ and the tetrapeptide-based linker technology stabilizes the ADC in plasma to reduce the risk of systemic toxicity.^500^ Enhertu was approved by the US FDA in 2019 based on positive results from a single-arm, multicenter, Phase II DESTINY-Breast01 study.^501^ Subsequently, Enhertu has also been approved for gastric cancer.^502^
- Sacituzumab govitecan (Trodelvy) comprises a humanized monoclonal antibody targeting Trop-2 conjugated to a topoisomerase I inhibitor SN-38 by means of a hydrolyzable linker (CL2A) with an average DAR of ∼7.6. Overexpression of Trop-2, a 40-kDa glycoprotein that plays a role as transducer of intracellular calcium signaling,^503,504^ was observed in the majority of solid tumors, including triple-negative breast cancer.^505^ The payload SN-38 inhibits DNA topoisomerase I thus causing DNA single strand breaks and eventually leads to cell death.^506^ The CL2A linker improves the binding ratio of Trop-2 antibody to SN-38, with higher toxic concentration in tumor but lower concentration in nontarget tissues.^507^ Linker optimization allows both controlled release of the drug and diffusion through the cell membrane, enabling the drug to kill neighboring tumor cells (the bystander effect).^508^ Sacituzumab govitecan received accelerated approval by the US FDA in 2020, for the treatment of patients with unresectable locally advanced or metastatic triple-negative breast cancer who have received two or more prior systemic therapies, at least one of them for metastatic disease. The clinical efficacy of sacituzumab govitecan was further confirmed in a multicenter, open-label, randomized trial (ASCENT),^509^ which promoted the US FDA to grant a regular approval.^510,511^
- Cetuximab sarotalocan (Akalux) includes an anti-EGFR chimeric monoclonal antibody, cetuximab, conjugated with IRDye700DX, a near-infrared photosensitizing dye.^512^ The average DAR of cetuximab sarotalocan was in the 1.3–3.8 range. EGFR is amply expressed on the surface of multiple kinds of solid tumors, including head and neck squamous cell carcinomas, esophageal cancer, lung cancer, colon cancer, pancreatic cancer and other solid tumors.^513^ Cetuximab sarotalocan targets EGFR and is locally activated using a laser to accurately induce the rapid death of cancer cells without damaging surrounding normal tissues.^514^ Cetuximab sarotalocan was approved by the Pharmaceuticals and Medical Devices Agency (PMDA) of Japan in 2019 as a treatment product of near-infrared photoimmunotherapy for unresectable locally advanced or recurrent head and neck squamous cell carcinoma.^73^ The approval was supported by the positive data from a multicenter, open-label Phase IIa trial.^515^
- Disitamab vedotin (Aidixi) consists of a humanized HER2 antibody, a cathepsin cleavable linker (mc-VC-PABC), and a cytotoxic agent, MMAE, with an average DAR ∼ 4.^516^ In June 2021, disitamab vedotin was conditionally approved by the National Medical Products Administration (NMPA) of China for the treatment of patients with locally advanced or metastatic gastric cancer, including gastroesophageal junction adenocarcinoma, who have received at least 2 types of systemic chemotherapy. The approval was supported by the results of the RC48-C008 study, which demonstrated a clinically significant response and survival benefit for patients receiving the drug.^517^ Disitamab vedotin has also conditionally approval by NMPA in December 2021 for treatment of patients with HER2 positive locally advanced or metastatic urothelial cancer who have also previously received platinum-containing chemotherapy treatment. It was also supported by the results of the RC48-C005 study, an open-label, multicenter, single-arm, nonrandomized Phase II study.^518^
- Tisotumab vedotin (Tivdak) contains a fully humanized mAb binding to tissue factor, a cleavable mc-VC-PABC linker, and an antimitotic agent, MMAE, with an average DAR of 4.^519^ Tissue factor is overexpressed on several solid tumors.^520^ Bystander effect, and antibody-dependent cellular cytotoxicity and phagocytosis have been also reported to be involved in the mechanism of action of tisotumab vedotin.^520^ Tivdak was approved by the US FDA in September 2021, for patients with recurrent or metastatic cervical cancer with disease progression on or after chemotherapy. The approval was supported by findings from the innovaTV 204 study, a multicenter, open-label, single-arm, Phase II trial.^521^
- Mirvetuximab soravtansine (Elahere) comprises a humanized mAb targeting folate receptor alpha (FRα) conjugated to a potent cytotoxic DM4 by a cleavable linker (sulfo-SPDB), for the treatment of ovarian cancer as orphan drug designation.^522^ FRα is expressed at high levels in most cases of epithelial ovarian cancer as well as in endometrial cancer and lung adenocarcinoma.^522^ Most normal tissues do not express FRα, making it a promising target for ADC.^522^ The hydrophilicity of the linker is increased by the introduction of a sulfonate group, while the addition of methyl groups α to the disulfide moiety reduces premature release of the drug in circulation. Mirvetuximab soravtansine was approved by the US FDA in November 2022 for the treatment of patients with FRα positive, platinum-resistant epithelial ovarian, fallopian tube, or primary peritoneal cancer, who have received one to three prior systemic treatment regimens.^78,523^

#### 7.3.3 Combination Therapy

In addition to the above approved ADCs, on April 3, 2023, the FDA granted accelerated approval to enfortumab vedotin-ejfv (Padcev, Astellas Pharma) with pembrolizumab (Keytruda, Merck) for treatment of locally advanced or metastatic urothelial carcinoma in patients ineligible for cisplatin-containing chemotherapy.^80,524^ Another two combination treatments including pembrolizumab (Keytruda, Merck) are in a late stage of clinical trials: datopotamab deruxtecan (Dato-DXd) with pembrolizumab (Keytruda) plus platinum-based chemotherapy,^525,526^ and sacituzumab govitecan-hziy (Trodelvy, Gilead) and pembrolizumab (Keytruda),^527,528^ both for treatment of non-small cell lung cancer (NSCLC).

## 8 Noteworthy Patents

ADC patents within the CAS Content Collection continue to grow not only in numbers but also in ADC technological diversity. The diversity of ADC compounds, linker technology, bioconjugation techniques, target antigen moieties, and diseases treated are highlighted with a selection of notable patents presented in Table 7.

**Table 7:** Notable ADC Patents

Patent number	Year	Assignee, location	Description
WO2009140242	2009	Genentech, USA	Analysis of ADCs by bead-based affinity capture and mass spectrometry
US9364554B2	2013	Centrose, USA	Extracellular-targeted drug conjugates (not internalized) in which an antibody or other targeting agent is linked to a drug through a linker
WO2017009258	2017	Genmab, Denmark	Axl-specific antibody–drug conjugates for cancer treatment
US10772965	2018	RC Biotechnologies, USA	Covalent linkers in ADCs and methods of making and using the same. Provides novel and advantageous compositions having a linker capable of covalently coupling one or more free thiols of an antibody which can be used in ADCs.
WO2019219891	2019	Daiichi Sankyo, Japan	Anti-MUC1 ADCs for treating cancer, infection, autoimmune disease, or immunodeficiency. The conjugates consist of exatecan derivatives coupled to anti MUC1 antibodies.
US20190099499	2019	Pfizer, USA	Cysteine-engineered ADCs for site-specific conjugation.
CN109106951	2019	Sichuan Baili Pharmaceutical, China	Camptothecin-antibody conjugate and application in treating tumors, autoimmune, or infectious diseases
WO2019126691	2019	Mersana Therapeutics, USA	ADCs comprising PBD drug moieties and methods of using these conjugates as therapeutics and/or diagnostics.
WO2020112588	2020	Bristol-Myers Squibb, USA	Engineering ADCs with glutamine-containing extensions on the C-terminus of the light chain using transglutaminase for improved stability and pharmacokinetics
US20200114018	2020	Genentech, USA	Methods of treating residual breast cancer with the ADC trastuzumab emtansine
WO2020180121	2020	LegoChem Biosciences, South Korea| Y-Biologics, South Korea	ADC comprising an antibody binding to DLK1 or an antigen-binding fragment, and a pharmaceutical agent
WO2020006449	2020	GO Therapeutics, USA	Antiglyco-MUC1 antibodies and antigen-binding fragments that specifically bind to a cancer-specific glycosylation variant of MUC1 treat cancer
WO2020075817	2020	Takeda, Japan	Method for manufacturing an ADC by using a microreactor. The method includes mixing, using a microreactor, a solution containing triscarboxyethyl phosphine, and IgG antibody in a reduction reaction initiated by TCEP, and a solution containing a TCEP inhibitor
WO2021076196	2021	Genentech, USA| Hoffmann-La Roche, Switzerland	Anti-cd79b immunoconjugates to treat diffuse large b-cell lymphoma comprising anti-CD79b antibodies in combination with an anti-CD20 antibody and one or more chemotherapeutic agents (such as gemcitabine and oxaliplatin)
WO2021202984	2021	Mersana Therapeutics, USA	ADCs comprising STING agonists and their use for the treatment of cancer
WO2021259928	2021	Sapreme Technologies, Netherlands| Charite - Universitaetsmedizin Berlin, Germany	ADC or an antibody-oligonucleotide conjugate comprising a VHH and a saponin or a ligand-saponin conjugate
WO2021222783	2021	Angiex, USA	ADC comprising an anti-TM4SFl antibody or an antigen-binding fragment and a proteasome inhibitor with an anti-TM4SFl antibody or antigen-binding fragment with a modified IgG Fc region with one or more substitutions
WO2022199429	2022	Chengdu Scimount Pharmatech, China	Preparation method for dual-drug-linker of ADC and use in cancer treatment
US20220226494	2022	AbbVie, USA	Anti-EGFR ADCs which inhibit Bcl-xL
WO2022262772	2022	Beijing Sinotau Bio-Pharmaceuticals Technology, China	Engineering of HER3 antibody and use in antibody–drug conjugates for cancer immunotherapy
WO2022136642	2022	Sotio Biotech, Czech Republic	Tumor-specific claudin 18.2 ADCs where the antibody or fragment exhibits increased binding to tumor tissue expressing CLDN18.2 over healthy tissue expressing CLDN18.2
WO2022153195	2022	Memorial Sloan Kettering Cancer Center, USA|Tri-Institutional Therapeutics Discovery Institute, USA	Anti-DLL3 antibody–drug conjugate
WO2022184082	2022	Sorrento Therapeutics, USA|Levena (Suzhou) Biopharma, China	Antibody–drug conjugates comprising an anti-B-cell maturation antigen antibody
US20220162308	2022	Novartis, Switzerland	Engineering anti-CD48 antibody–drug conjugates for use in cancer immunotherapy
WO2023274974	2023	ADC Therapeutics, Switzerland| MedImmune, USA	Combination therapy using anti-CD19 ADCs and anti-CD79b conjugates
WO2023070125	2023	Academia Sinica, Taiwan| Liu, Fu-Tong, Taiwan	Antibody–drug conjugate for reducing glycosylation of membrane glycoprotein comprising an antibody or antigen-binding fragment, an oligosaccharyltransferase inhibitor, and a linker
WO2023275112	2023	Rigshospitalet, Denmark| University of Copenhagen, Denmark	ADCs comprising humanized antibodies targeting uPARAP
WO2023281445	2023	TechnoPhage, Portugal|Faculty of Veterinary Medicine of the University of Lisbon, Portugal	Highly specific rabbit anti-cNHL single domain antibodies conjugated with antitumor payload for delivery through the blood brain barrier to the CNS for cancer immunotherapy.

## 9 Outlook and Perspectives

Over the past decade, ADCs have made tremendous progress as a result of optimization of the choice of cytotoxic agents, conjugation strategies, better selection of targeted antigens, and improved antibody engineering. However, despite their sophisticated design, ADCs are still associated with certain limitations and the emergence of resistance mechanisms. To overcome these limitations, new antibody formats, new delivery systems, antigenic targets, cytotoxic payloads, and site-specific conjugation methods have continued to be developed and advanced.

### 9.1 Major Challenges and Perspectives for Antibody–Drug Conjugate Development

The advance of ADCs involves certain challenges that researchers and developers need to overcome in their efforts to design successful ADCs:

- **Complex design and manufacturing.** ADCs are complex molecules that require precise conjugation of the antibody, linker, and cytotoxic drug payload. The manufacturing process can be challenging and involves multiple steps, increasing the complexity and cost of production.
- **Target selection:** Identifying appropriate target antigens that are selectively expressed on cancer cells is a critical challenge. The target antigen should be highly specific to cancer cells to minimize off-target effects and maximize therapeutic efficacy. However, not all cancers have well-defined target antigens, and heterogeneity of antigen expression within tumors can further complicate target selection.
- **Heterogeneity of target expression.** Even when a target antigen is identified, its expression can vary within and between tumors. Heterogeneous antigen expression may result in incomplete target binding and reduce the effectiveness of ADC therapy. Moreover, antigen loss or downregulation can occur during treatment, leading to acquired resistance.
- **Payload selection and optimization.** Selecting an appropriate cytotoxic drug payload is crucial for the potency and effectiveness of ADCs. The cytotoxic drug should have high lethality against cancer cells while maintaining stability during conjugation and circulation. Optimizing the delicate balance between drug potency and linker stability is a complex task that requires careful consideration of the desired mechanism of action and the specific characteristics of the target cancer type.
- **Linker design and stability.** Designing an optimal linker that balances stability, selective cleavage, and efficient drug release is a significant challenge. The linker must be stable during circulation to minimize premature drug release, but it should also be able to efficiently release the cytotoxic drug inside the target cell. Achieving the right balance between linker stability and cleavability is crucial for maximizing therapeutic efficacy.
- **Pharmacokinetics and biodistribution.** Optimizing the pharmacokinetic properties of ADCs is essential for effective drug delivery to tumor sites. ADCs need to have appropriate systemic circulation, tumor penetration, and retention within the tumor microenvironment. Achieving optimal pharmacokinetics and biodistribution can be challenging due to factors such as rapid clearance, limited tumor penetration, and inadequate tumor-specific accumulation.
- **Maximum tolerated dose (MTD).** According to a recent report, the MTDs of ADCs and the related payload small molecule drugs in humans are nearly the same after normalization for cytotoxic agent content,^529,530^ i.e., current ADCs do not substantially increase the MTDs of their conjugated drugs regardless of their broad diversity. Thus, mechanisms that may provide further improvements in efficacy and tolerability need to be further explored, with in-depth analysis of the pharmacokinetic/pharmacodynamic formulation profiles for optimizing ADC clinical dosing strategies.
- **Manufacturing complexity and scale-up.** ADCs are complex molecules that require precise conjugation of the antibody, linker, and payload. The manufacturing process needs to be scalable, reproducible, and cost-effective. Ensuring consistent quality control throughout the manufacturing process is a significant challenge, especially when dealing with multiple components and their interactions.
- **DAR heterogeneity.** Achieving a consistent and predictable DAR can be difficult for multiple reasons, including manufacturing challenges such as conjugation chemistry and purification; different conjugation techniques leading to higher or lower drug loading on the antibodies; heterogeneity in antibody population, etc.
- **Immunogenicity and safety.** ADCs can induce immune responses, leading to reduced efficacy and potential safety concerns. Minimizing immunogenicity is a challenge that requires careful antibody engineering and testing. Ensuring the safety profile of ADCs, including minimizing off-target effects and potential toxicities, is also a critical consideration.
- **Off-target effects.** While ADCs are designed to target cancer cells selectively, there is a possibility of off-target effects, where the ADC binds to noncancerous cells expressing low levels of the target antigen. This can lead to undesired toxicity in healthy tissues and potential side effects.
- **Resistance mechanisms.** Cancer cells can develop resistance to ADC therapy through various mechanisms, such as antigen loss, altered drug uptake, or drug efflux pumps. These resistance mechanisms can limit the effectiveness of ADCs and reduce treatment efficacy over time.
- **Regulatory approval.** ADCs involve the combination of different components, including an antibody, cytotoxic drug, and linker. Each component may require separate regulatory approval processes, which can be time-consuming and expensive. Meeting regulatory requirements for safety and efficacy is a significant challenge in ADC development.
- **Cost.** ADC therapy can be costly due to the complexity of manufacturing, including the need for highly specialized equipment and processes. The high cost can limit accessibility and affordability for patients.

Addressing these challenges requires continuous advancements in antibody engineering, linker technology, payload design, and the understanding of tumor biology. Collaboration among researchers, pharmaceutical companies, and regulatory agencies is essential to overcome these challenges and bring effective ADC therapies to patients.

An important advance in the development and use of ADC is in **combination therapies**.^3,275,319,531,532^ Combining ADCs with other treatment modalities, such as chemotherapy, radiation therapy, or immunotherapies, is an area of active research. Researchers investigate synergistic effects and explore combination strategies to enhance therapeutic outcomes and overcome resistance mechanisms. Noteworthy, on April 3, 2023, the FDA granted accelerated approval to the combination of enfortumab vedotin-ejfv (Padcev, Astellas Pharma) with pembrolizumab (Keytruda, Merck) for treatment of locally advanced or metastatic urothelial carcinoma in patients ineligible for cisplatin-containing chemotherapy.^80^

Another significant advance in ADC development is **companion diagnostics**.^3,533−536^ Biomarker identification and patient stratification based on target expression levels or other predictive factors can help select the most appropriate patients for treatment and improve the clinical outcome of the ADC treatment.

**Nanobody-Enhanced ADCs** offer another possibility to enhance therapeutic effects. Nanobodies are small, stable, and highly specific, making them suitable for targeting tumor-specific antigens.^537,538^ When used as the targeting ligands in ADCs, nanobodies offer several advantages: (i) Reduced immunogenicity: nanobodies are derived from camelids or engineered from human antibodies, which can lead to reduced immunogenicity compared to larger antibodies; (ii) Enhanced tissue penetration: their smaller size can improve tissue penetration within solid tumors; (iii) Rapid clearance from circulation: this can reduce off-target effects and systemic toxicity.

Bispecific antibodies are engineered to simultaneously bind to two different antigens or epitopes.^539^ This enables them to target two distinct molecular targets, often with therapeutic advantages. **Bispecific antibodies in ADCs** can simultaneously target tumor-specific antigens and immune cells, facilitating immune-mediated killing of cancer cells.^540,541^ This can enhance therapeutic effects and potentially reduce resistance. For example, a bispecific ADC can bind to a tumor antigen on one arm and CD3 on the other, engaging T cells for tumor cell destruction.

**Trispecific antibodies** are designed to bind to three different targets or antigens. They offer even greater targeting specificity, potentially allowing for more precise delivery of cytotoxic payloads to tumor cells while sparing healthy tissues.^542^

By integration of these advanced antibody formats into ADCs, it becomes possible to achieve several goals simultaneously: enhanced target specificity, improved tissue penetration, engagement of the immune system, and reduction in systemic toxicity. These advanced ADC strategies hold great promise for improving the efficacy and safety of cancer therapies and other targeted treatments.

ADC design and use are constantly evolving, driven by advancements in antibody engineering, linker technology, payload development and diversification, and improvements in our understanding of the biology of cancer and other diseases. Enhancements in the specificity, potency, and safety of ADC are necessary to improve their therapeutic indexes and clinical effectiveness.^18^

In conclusion, ADCs are a promising therapeutic modality, particularly in combination with chemotherapies, immune checkpoint inhibitors, or other therapies. In the past decade, the development of novel targets, linkers, and payloads have fueled advances in ADC, including applications beyond oncology. ADCs provide the possibility of endowing drugs from the past with improved pharmacokinetics and reduced toxicity, making them useful once more.

## Supporting Information Available

ADC preclinical trials; ADC clinical trials (PDF)
