# Supplementary material for: The Evolving Landscape of Antibody–Drug Conjugates: In Depth Analysis of Recent Research Progress
Source: Bioconjug Chem. 2023 Oct 11;34(11):1951–2000. doi: 10.1021/acs.bioconjchem.3c00374 (PMC10655051; doi:10.1021/acs.bioconjchem.3c00374)
Supplement: Supplementary file 1 — bc3c00374_si_001.pdf [file bc3c00374_si_001.pdf]

Supporting Information

The Evolving Landscape of Antibody-Drug Conjugates: In Depth Analysis of Recent Research Progress

Janet M. Sasso†1, Rumiana Tenchov†1, Robert Bird[1], Kavita A. Iyer[2], Krittika Ralhan[2], Yacidzohara Rodriguez[1], Qiongqiong Angela Zhou*1

[1]CAS, a division of the American Chemical Society, Columbus OH 43210, USA

[2]ACS International India Pvt. Ltd., Pune-411044, India

†Authors J.M.S. and R.T. contributed equally to this paper

*Corresponding author: qzhou@cas.org

Table S1. ADC preclinical trials

ADC Name	Company	Condition	Target	Citation
ADCT-212	ADC Therapeutics, Switzerland	Prostate Cancer	PSMA	https://www.adctherapeutics.com/our-pipeline/
ADCT-701	ADC Therapeutics, Switzerland	Solid tumors	DLK1	https://www.adctherapeutics.com/our-pipeline/
AGX101	Angiex, USA	Solid tumors	TM4SF1	https://angiex.com/pipeline
ALT-Q5	Alteogen, South Korea	Ovarian cancer	FRα	http://www.alteogen.com/en/pipeline_1/
AMGEN/Synaffix Collaboration	AMGEN, USA/ Synaffix, Netherlands	Undisclosed	Undisclosed	https://synaffix.com/partnered-pipeline/
ARX305 | NCB002	Ambrx, USA/ NovoCodex, China	Solid and Hematologic tumors	CD70	https://ambrx.com/pipeline/
ATRC-301	Zymeworks, Canada l Atreca, USA	Solid tumors	EphA2	https://www.zymeworks.com/pipeline/
AT-00	Axcynsis Therapeutics, Singapore	Undisclosed	Undisclosed	https://axcynsis.com/technology/
AT-01	Axcynsis Therapeutics, Singapore	Liquid tumors l Solid tumors	Undisclosed	https://axcynsis.com/technology/
AT-03	Axcynsis Therapeutics, Singapore	Solid tumors	Undisclosed	https://axcynsis.com/technology/

AT-04	Axcynsis Therapeutics, Singapore	Solid tumors	Undisclosed	https://axcynsis.com/technology/
AVP10	Avipep Therapeutics, Australia	Hodgkin's lymphoma l Cutaneous lymphoma l Anaplastic large cell lymphoma	Undisclosed	http://avipep.com/technology/pipeline/
AVP20	Avipep Therapeutics, Australia	Undisclosed	Undisclosed	http://avipep.com/technology/pipeline/
BA1302	Luye Pharma, China	Colorectal cancer l Breast cancer l Non-small cell lung cancer l Pancreatic cancer	Undisclosed	https://www.luye.cn/lvye_en/innovate.php#zycpx
BA3151	Bioatla, USA	Solid tumors	B7H4	https://www.bioatla.com/cab-portfolio/
BA3361	Bioatla, USA	Solid tumors	Nectin-4	https://www.bioatla.com/cab-portfolio/
BCMA ADC	Sorrento, USA	Liquid tumors	BCMA	https://sorrentotherapeutics.com/research/pipeline/
BIO-107	BiOneCure Therapeutics, USA	Acute myeloid leukemia	Undisclosed	https://www.bionecure.com/pipeline/
BIO-108	BiOneCure Therapeutics, USA	Solid tumors	Undisclosed	https://www.bionecure.com/pipeline/

BIO-109	BiOneCure Therapeutics, USA	Solid tumors	Undisclosed	https://www.bionecure.com/pipeline/
BIO-110	BiOneCure Therapeutics, USA	Solid tumors	Undisclosed	https://www.bionecure.com/pipeline/
BIO-112	BiOneCure Therapeutics, USA	Solid tumors	Undisclosed	https://www.bionecure.com/pipeline/
BIO-201	BiOneCure Therapeutics, USA	Solid tumors	Undisclosed	https://www.bionecure.com/pipeline/
BRY812	BioRay Pharmaceutical, China	Solid tumors	LIV-1	http://www.biopharm.com/index.php?m=list&a=index&classid=21
BVX001	BiVictriX Therapeutics, UK	Acute myeloid leukemia	CD7 I CD33 (bispecific)	https://bivictrix.com/science/
BVX002	BiVictriX Therapeutics, UK	Ovarian cancer	Undisclosed	https://bivictrix.com/science/
BVX003	BiVictriX Therapeutics, UK	Bladder cancer	Undisclosed	https://bivictrix.com/science/
BYON4413	Byondis, Netherlands	Solid tumors	CD123	https://www.byondis.com/pipeline
CM-09	CureMeta, USA	Gastric Cancer I Pancreatic cancer	TRA-1-60	https://www.curemeta.com/cancer-target-and-antibody
CRB-701 I SYS6002	Corbus, USA	Bladder cancer I	Nectin-4	https://www.corbuspharma.com/our-pipeline

		Solid Tumors		
CS-2051	CytomX Therapeutics, USA	Solid tumors	EpCAM	https://cytomx.com/pipeline/
CX-2051	CytomX Therapeutics, USA	Solid tumors	EpCAM	https://cytomx.com/pipeline/
D3-GPC2-PBD	The Children's Hospital of Philadelphia, USA	Neuroblastoma	CPC2	https://jitc.bmj.com/content/10/12/e004704.long
DB-1311	Duality Biologics, China	Solid tumors	Undisclosed	https://www.dualitybiologics.com/pipeline.html
DB-1312	Duality Biologics, China	Solid tumors	Undisclosed	https://www.dualitybiologics.com/pipeline.html
DB-2304	Duality Biologics, China	Autoimmune Disease	Undisclosed	https://www.dualitybiologics.com/pipeline.html
DB-2306	Duality Biologics, China	Autoimmune Disease	Undisclosed	https://www.dualitybiologics.com/pipeline.html
EDC1 (DYS-ADC)	Centrose, USA	Pancreatic Cancer	Dysadherin	https://www.centrosepharma.com/edc1
EDC8	Centrose, USA	Acute myeloid leukemia	CD38	https://www.centrosepharma.com/edc8
EDC9 (EDC-CD20)	Centrose, USA	Diffuse large B cell lymphoma	CD20	https://www.centrosepharma.com/edc9

EP-400	Esperance Pharmaceutical, USA	Solid tumors l Liquid tumors	multiple	http://www.esperancepharma.com/media/user/file/Esperance%20Executive%20Summary%202016.pdf
ETx-22	Emergence Therapeutics, USA l Mablink, France	Bladder cancer l Breast cancer l Ovarian cancer l Head and neck cancer l Lung cancer	Nectin-4	https://emergencetx.com/
GMF-1A3 / GMF-1A3-MMAE	Kabara Cancer Research Institute, USA	Breast cancer	Amphiregulin	https://doi.org/10.1093/abt/tbac020
GQ1007	GeneQuantum Healthcare, China	Cold tumors	Undisclosed	http://www.genequantum.com/#/common/product?pid=12&id=13&type=9
GQ1009	GeneQuantum Healthcare, China	Undisclosed	Undisclosed	http://www.genequantum.com/#/common/product?pid=12&id=13&type=11
GQ1010	GeneQuantum Healthcare, China	Lung cancer l Breast cancer l Solid tumors	Undisclosed	http://www.genequantum.com/#/common/product?pid=12&id=13&type=10
GQ1011	GeneQuantum	Undisclosed	Undisclosed	http://www.genequantum.com/#/common/product?pid=12&id=13&type=12

	Healthcare, China			
GQ1014	GeneQuantum Healthcare, China	Undisclosed	Undisclosed	http://www.genequantum.com/#/common/product?pid=12&id=13&type=13
GQ1015	GeneQuantum Healthcare, China	Undisclosed	Undisclosed	http://www.genequantum.com/#/common/product?pid=12&id=13&type=14
GQ1016	GeneQuantum Healthcare, China	Undisclosed	Undisclosed	http://www.genequantum.com/#/common/product?pid=12&id=13&type=15
GQ1018	GeneQuantum Healthcare, China	Undisclosed	Undisclosed	http://www.genequantum.com/#/common/product?pid=12&id=13&type=16
HDP-102	Heidelberg Pharma, Germany	Non-Hodgkin's lymphoma	CD37	https://heidelberg-pharma.com/en/research-development/portfolio-overview
HDP-103	Heidelberg Pharma, Germany	Prostate Cancer	PSMA	https://heidelberg-pharma.com/en/research-development/portfolio-overview
HDP-104	Heidelberg Pharma, Germany	Gastrointestinal tumor	Guanylyl cyclase C	https://heidelberg-pharma.com/en/research-development/portfolio-overview
HDP-XX	Heidelberg Pharma, Germany	Solid and Hematologic tumors	Undisclosed	https://heidelberg-pharma.com/en/research-development/portfolio-overview
IKS012	Iksuda Therapeutics, UK	Ovarian cancer l Lung cancer	FOLR1	https://iksuda.com/pipeline/

		l Breast cancer		
IKS02	Iksuda Therapeutics, UK	Solid tumors	Undisclosed	https://iksuda.com/pipeline/
IKS04	Iksuda Therapeutics, UK	Solid tumors	Undisclosed	https://iksuda.com/pipeline/
IMB-212	ImmunoBiochem, Canada	Solid tumors	Undisclosed	https://www.immunobiochem.com/pipeline
IMB-213I	ImmunoBiochem, Canada	Solid tumors	Undisclosed	https://www.immunobiochem.com/pipeline
Intocell ADC 1	Intocell, South Korea	Solid tumors	B7H3	https://intocell.com/bbs/content.php?co_id=pipeline
Intocell ADC 2	Intocell, South Korea	Solid tumors	TROP2	https://intocell.com/bbs/content.php?co_id=pipeline
Intocell ADC 3	Intocell, South Korea	Undisclosed	HER2	https://intocell.com/bbs/content.php?co_id=pipeline
Intocell ADC 4	Intocell, South Korea	Solid tumors	HER3	https://intocell.com/bbs/content.php?co_id=pipeline
Intocell ADC 5	Intocell, South Korea	Undisclosed	Undisclosed	https://intocell.com/bbs/content.php?co_id=pipeline
Intocell ADC 6	Intocell, South Korea	Undisclosed	Undisclosed	https://intocell.com/bbs/content.php?co_id=pipeline
IO-0001	Orum Therapeutics, South Korea	Melanoma	PD-1	https://docsend.com/view/enn7dddfbbn74sr2
Janssen/Mersana Collaboration	Mersana Therapettics l Janssen	Undisclosed	Undisclosed	https://www.mersana.com/pipeline/overview/
Lantern Pharma ADC	Lantern Pharma, USA	Solid tumors	Undisclosed	https://www.lanternpharma.com/pipeline#lp100
LCB02A	Legochembio, South Korea	Solid tumors l	Undisclosed	https://www.legochembio.com/pipeline/pipeline.php?lang=k

	l Harbour Biomed, China	Liquid tumors		
LCB04A	Legochembio, South Korea	Solid tumors l Liquid tumors	Undisclosed	https://www.legochembio.com/pipeline/pipeline.php?lang=k
LCB06A	Legochembio, South Korea	Solid tumors l Liquid tumors	Undisclosed	https://www.legochembio.com/pipeline/pipeline.php?lang=k
LCB07A	Legochembio, South Korea l ABL Bio, South Korea	Solid tumors l Liquid tumors	Undisclosed	https://www.legochembio.com/pipeline/pipeline.php?lang=k
LCB09A	Legochembio, South Korea	Solid tumors l Liquid tumors	Undisclosed	https://www.legochembio.com/pipeline/pipeline.php?lang=k
LCB12A	Legochembio, South Korea l Hanmi, South Korea	Undisclosed	Undisclosed	https://www.legochembio.com/pipeline/pipeline.php?lang=k
LCB18A	Legochembio, South Korea	Undisclosed	Undisclosed	https://www.legochembio.com/pipeline/pipeline.php?lang=k
LCB19A	Legochembio, South Korea l ATG antengene, China	Undisclosed	Undisclosed	https://www.legochembio.com/pipeline/pipeline.php?lang=k
LCB20A	Legochembio, South Korea l Sotio, Czech Republic	Undisclosed	Undisclosed	https://www.legochembio.com/pipeline/pipeline.php?lang=k

LCB36A	Legochembio, South Korea	Undisclosed	Undisclosed	https://www.legochembio.com/pipeline/pipeline.php?lang=k
LCB42A	Legochembio, South Korea l Amgen, USA	Undisclosed	Undisclosed	https://www.legochembio.com/pipeline/pipeline.php?lang=k
LCB67	Legochembio, South Korea l Y Biologics, South Korea	Small-cell lung cancer l Hepatocellular carcinoma l Neuroblastoma l Myelodysplastic syndrome l Acute myeloid leukemia	DLK1	https://www.legochembio.com/pipeline/pipeline.php?lang=k
LCB69	Legochembio, South Korea l Takeda, Japan	Solid tumors l Liquid tumors	Undisclosed	https://www.legochembio.com/pipeline/pipeline.php?lang=k
LCB84	Legochembio, South Korea l Mediterranea, Lebanon	Solid tumors	Undisclosed	https://www.legochembio.com/pipeline/pipeline.php?lang=k
LCB85	Legochembio, South Korea l Iksuda, USA	Solid tumors l Liquid tumors	Undisclosed	https://www.legochembio.com/pipeline/pipeline.php?lang=k
LCB91	Legochembio, South Korea	Solid tumors l	Undisclosed	https://www.legochembio.com/pipeline/pipeline.php?lang=k

		Liquid tumors		
LCB97	Legochembio, South Korea l Elthera, Switzerland	Solid tumors	Undisclosed	https://www.legochembio.com/pipeline/pipeline.php?lang=k
LM-001	LaNova Medicines, China	Solid tumors	Undisclosed	https://www.lanovamedicines.com/en/pipeline
LM-004	LaNova Medicines, China	Solid tumors	Undisclosed	https://www.lanovamedicines.com/en/pipeline
LM-005	LaNova Medicines, China	Solid tumors	Undisclosed	https://www.lanovamedicines.com/en/pipeline
LM-006	LaNova Medicines, China	Solid tumors	Undisclosed	https://www.lanovamedicines.com/en/pipeline
LM-007	LaNova Medicines, China	Solid tumors	Undisclosed	https://www.lanovamedicines.com/en/pipeline
LM-008	LaNova Medicines, China	Solid tumors	Undisclosed	https://www.lanovamedicines.com/en/pipeline
MBK-103	Mablink, France	Gynecological cancer l Breast Cancer l Non-small cell lung cancer l Colorectal cancer	FRα	https://www.mablink.com/mbk-103

MBK-104	Mablink, France	Solid tumors	Undisclosed	https://www.mablink.com/adc-pipeline
MBK-105	Mablink, France	Solid tumors	Undisclosed	https://www.mablink.com/adc-pipeline
MBK-101	Mablink, France	Acute myeloid leukemia	Undisclosed	https://www.mablink.com/adc-pipeline
MBK-102	Mablink, France	Non-Hodgkin's lymphoma	Undisclosed	https://www.mablink.com/adc-pipeline
Merck/Mersana Collaboration 1	Mersana Therapeutics, USA l Merck, USA	Undisclosed	Undisclosed	https://www.mersana.com/pipeline/overview/
Merck/Mersana Collaboration 2	Mersana Therapeutics, USA l Merck, USA	Undisclosed	Undisclosed	https://www.mersana.com/pipeline/overview/
MP-1959-ADC	Mediapharma, Italy	Breast cancer l Prostate cancer l Ovarian cancer l Pancreatic cancer l Lung cancer l Melanoma l Glioblastoma l Neuroblastoma	Gal-3 BP	https://www.mediapharma.it/product-pipeline

MP-EV20-ADC	Mediapharma, Italy	Breast cancer (wild type or resistant to Trastuzumab and T-DM1) l Melanoma l Lung cancer l Colorectal cancer l Prostate cancer	HER3	https://www.mediapharma.it/product-pipeline
MRG006	Miracogen, China	Solid tumors	Undisclosed	http://www.miracogen.com.cn/en/Index/lists/catid/9.html
McSAF01	McSAF, France	Solid tumors	Undisclosed	https://mcsaf.fr/mcsaf-inside-oncology/
McSAF02	McSAF, France	Liquid tumors	Undisclosed	https://mcsaf.fr/mcsaf-inside-oncology/
MT-8633	Mitsubishi Tanabe Pharma	Solid tumor	c-MET	https://www.mt-pharma.co.jp/e/develop/pipeline.html
MediLink Therapeutics project 3	MediLink Therapeutics, China	Solid tumors	Undisclosed	https://www.medilinkthera.com/pipeline
MediLink Therapeutics project 4	MediLink Therapeutics, China	Solid tumors	Undisclosed	https://www.medilinkthera.com/pipeline
MediLink Therapeutics project 5	MediLink Therapeutics, China	Solid tumors	Undisclosed	https://www.medilinkthera.com/pipeline
MediLink Therapeutics project 6	MediLink Therapeutics, China	Solid tumors	Undisclosed	https://www.medilinkthera.com/pipeline

MediLink Therapeutics project 7	MediLink Therapeutics, China	Solid tumors	Undisclosed	https://www.medilinkthera.com/pipeline
NBE Partner Program I	NBE Therapeutics, Switzerland l Sotio, Czech Republic	Solid tumors	Undisclosed	https://nbe-therapeutics.com/pipeline
NBE Partner Program II	NBE Therapeutics, Switzerland l Sotio, Czech Republic	Solid tumors	Undisclosed	https://nbe-therapeutics.com/pipeline
NBE Partner Program III	NBE Therapeutics, Switzerland l Exelixis, USA	Cancer	Undisclosed	https://nbe-therapeutics.com/pipeline
NBE-105	NBE Therapeutics, Switzerland	Solid tumors	Undisclosed	https://nbe-therapeutics.com/pipeline
NBT528	NBE Therapeutics, Switzerland	Hodgkin's lymphoma	Undisclosed	http://www.newbiotherapeutics.com/list/?26_1.html
NBT558	NBE Therapeutics, Switzerland	Small-cell lung cancer	Undisclosed	http://www.newbiotherapeutics.com/list/?26_1.html
NBT568	NBE Therapeutics, Switzerland	Ovarian cancer	Undisclosed	http://www.newbiotherapeutics.com/list/?26_1.html
NBT578	NBE Therapeutics, Switzerland	Lung cancer l Gastric cancer	Undisclosed	http://www.newbiotherapeutics.com/list/?26_1.html

NV101	NanoValent Pharmaceuticals, USA	Ewing sarcoma	CD99	http://nanovalent.com/pipeline/
NV102	NanoValent Pharmaceuticals, USA	Acute lymphoblastic leukemia l Acute myeloid leukemia	CD19	http://nanovalent.com/pipeline/
NV103	NanoValent Pharmaceuticals, USA	Ewing sarcoma l Glioblastoma multiforme	CD99	http://nanovalent.com/pipeline/
NV104	NanoValent Pharmaceuticals, USA	Glioblastoma multiforme	B7H3	http://nanovalent.com/pipeline/
NV105	NanoValent Pharmaceuticals, USA	Glioblastoma multiforme	B7H3	http://nanovalent.com/pipeline/
NV106	NanoValent Pharmaceuticals, USA	Glioblastoma multiforme	B7H3	http://nanovalent.com/pipeline/
OMTX703	Oncomatryx, Spain	Ewing sarcoma l Uveal melanoma l Peripheral nerve sheath tumor	Endoglin	https://oncomatryx.com/adc-omtx703/
ORM-6151	Orum Therapeutics, South Korea	Acute myeloid leukemia	CD33	https://docsend.com/view/gkrqtx49dwmthe28

PHN-010	Pheon Therapeutics, UK	Solid tumors	Undisclosed	https://pheontx.com/pipeline/
PHN-020	Pheon Therapeutics, UK	Solid tumors	Undisclosed	https://pheontx.com/pipeline/
PLBA-001	PrimeLink Bio, China	Lung cancer	Undisclosed	http://en.primelinkbio.com/about_us_1/1.html
PLBA-002	PrimeLink Bio, China	Ovarian cancer	Undisclosed	http://en.primelinkbio.com/about_us_1/1.html
PLBA-003	PrimeLink Bio, China	Solid tumors	Undisclosed	http://en.primelinkbio.com/about_us_1/1.html
PLBA-004	PrimeLink Bio, China	Solid tumors	Undisclosed	http://en.primelinkbio.com/about_us_1/1.html
PLBA-005	PrimeLink Bio, China	Ovarian cancer	Undisclosed	http://en.primelinkbio.com/about_us_1/1.html
PLBC-001	PrimeLink Bio, China	Acute myeloid leukemia	Undisclosed	http://en.primelinkbio.com/about_us_1/1.html
PLBC-002	PrimeLink Bio, China	Breast cancer	Undisclosed	http://en.primelinkbio.com/about_us_1/1.html
PLBC-003	PrimeLink Bio, China	Pancreatic cancer	Undisclosed	http://en.primelinkbio.com/about_us_1/1.html
PRO1106	ProfoundBio, China	Solid tumors	Undisclosed	https://profoundbio.com/Pipeline
PRO1107	ProfoundBio, China	Solid tumors	Undisclosed	https://profoundbio.com/Pipeline
PRO1109	ProfoundBio, China	Solid tumors	Undisclosed	https://profoundbio.com/Pipeline
PRO1135	ProfoundBio, China	Solid tumors	Undisclosed	https://profoundbio.com/Pipeline
PRO1173	ProfoundBio, China	Solid tumors	Undisclosed	https://profoundbio.com/Pipeline

PRO1286	ProfoundBio, China	Solid tumors	Undisclosed	https://profoundbio.com/Pipeline
PRO1291	ProfoundBio, China	Solid tumors	Undisclosed	https://profoundbio.com/Pipeline
R-992	OBI-Pharma, Taiwan	Solid tumors	TROP2	https://www.obipharma.com/pipeline/
RC168	RemeGen, China	Solid tumors	Undisclosed	http://www.remegen.com/?v=listing&cid=92#cpgx
RC178	RemeGen, China	Solid tumors	Undisclosed	http://www.remegen.com/?v=listing&cid=92#cpgx
RC188	RemeGen, China	Solid tumors	Undisclosed	http://www.remegen.com/?v=listing&cid=92#cpgx
Shenogen ADC	Shenogen, China	Solid tumors	Undisclosed	http://www.shenogen.com/EN/products.aspx?BaseInfoCateId=49&CateId=49
SOT106	Sotio, Czech Republic l LegoChem Biosciences, South Korea	Solid tumors	Undisclosed	https://sotio.com/pipeline/antibody-drug-conjugates/sot106
SOT107	Sotio, Czech Republic l NBE Therapeutics, Switzerland	Solid tumors	Undisclosed	https://sotio.com/pipeline/antibody-drug-conjugates/sot107
STRO-003	Sutro Biopharma, USA	Solid tumors	ROR1	https://www.sutrobio.com/pipeline/
Sutro/Astellas Immunostimulatory ADC collaboration	Sutro Biopharma, USA l Astellas, Japan	Solid tumors	Undisclosed	https://www.sutrobio.com/pipeline/

XCN-010	Xiconic Pharmaceuticals, USA	Breast cancer l Non-small cell lung cancer l Prostate cancer l Mantle cell lymphoma l Gastric cancer l Ovarian cancer l Pancreatic cancer	Undisclosed	https://www.xiconicpharma.com/products
XB010	Exelixis, USA	Non-small cell lung cancer l Head and neck cancer l Gastric cancer l Breast cancer	Undisclosed	https://www.exelixis.com/pipeline/
XMT-2056	Mersana Therapeutics, USA	Breast cancer l Gastric cancer l Non-small cell lung cancer l Colorectal cancer	Undisclosed	https://www.mersana.com/xmt-2056/

XMT-2068	Mersana Therapeutics, USA	Undisclosed	Undisclosed	https://www.mersana.com/pipeline/overview/
XMT-2175	Mersana Therapeutics, USA	Undisclosed	Undisclosed	https://www.mersana.com/pipeline/overview/
YBL-001	Y Biologics, South Korea	Small-cell lung cancer l Liver cancer	DLK-1	https://ybiologics.com/en/sub/pipline/adc.php
ZW191	Zymeworks, Canada	Gynecologic cancer	FRα	https://www.zymeworks.com/pipeline/
ZW220	Zymeworks, Canada	Gynecologic cancer l Lung cancer	NaPi2b	https://www.zymeworks.com/pipeline/
ZW251	Zymeworks, Canada	Liver cancer l Hepatocelluar carcinoma	Glypican-3	https://www.zymeworks.com/pipeline/

Table S2. ADC clinical trials

ADC Name	Company	Condition	Target	mAb	Linker	Payload	Payload Action	Citation
7MW 3711	Mabwell, USA	Solid tumors	B7H3	...	...	...	Apoptosis stimulants, DNA topoisomerase I inhibitor	https://Mabwell.com/en/index.html#reloaded
9MW 2821	Mabwell, USA	Solid tumors	Nectin-4	MW282	Enzyme cleavable	MMAE	Tubulin polymerization inhibitor	https://Mabwell.com/en/index.html#reloaded
9MW 2921	Mabwell, USA	Solid tumors	TROP2	...	Enzyme cleavable	TOPli	Topoisomerase inhibitor	https://Mabwell.com/en/index.html#reloaded
A166 ADC	Klus Pharma, USA	Breast cancer	HER2	Trastuzumab	Enzyme cleavable	Duostatin-5	Microtubule inhibitor	https://www.kluspharma.com/pipeline
ABBV-011	AbbVie, USA	Small cell lung cancer	SEZ6	IgG1	Acid-labile hydrazine linker	Calicheamicin	DNA-damaging agent	https://www.abbvie.com/science/pipeline.html
ABBV-154	AbbVie, USA	Crohn's Disease I Polymyalgia Rheumatica	TNF	Adalimumab	...	Steroid	Glucocorticoid receptor agonist	https://www.abbvie.com/science/pipeline.html

ABBV-155	AbbVie, USA	Solid and Hematologic tumors	CD276	Mirzotamab	Peptide cleavable linker	Clezutoclax	BCL2 family protein inhibitor	https://www.abbvie.com/science/pipeline.html
ABBV-319	AbbVie, USA	Hematologic tumors	CD19	IgG1	...	GRM Steroid	Glucocorticoid receptor agonist	https://www.abbvie.com/science/pipeline.html
ABBV-400	AbbVie, USA	Solid tumors	c-Met	Telisotuzumab	Cleavable valine-alanine linker	...	Topoisomerase 1 inhibitor	https://www.abbvie.com/science/pipeline.html
ABBV-637	AbbVie, USA	Non-small cell lung cancer	EGFR	...	...	...	EGFR, BCL2 family protein inhibitor	https://www.abbvie.com/science/pipeline.html
ABBV-706	AbbVie, USA	Small cell lung cancer	SEZ6	...	...	...	DNA topoisomerase I inhibitors	https://www.abbvie.com/science/pipeline.html
ADCT-602	ADC Therapeutics, Switzerland	Acute Lymphoblastic Leukemia	CD22	Igαβ	Enzyme cleavable	PBD-Dimer	DNA cleavage	https://www.adctherapeutics.com/our-pipeline/
ADCT-601 | Mipasetamab	ADC Therapeutics, Switzerland	Solid tumors	AXL	IgG1κ	Enzyme cleavable	SG3199	DNA cross linking agents	https://www.adctherapeutics.com/our-pipeline/

Uzoptirine								
ADCT-901	ADC Therapeutics, Switzerland	Solid tumors	KAAG1	IgG1	Enzyme cleavable	SG3199	DNA cross linking agents	https://www.adctherapeutics.com/our-pipeline/
ALT-P7	Alteogen, South Korea	Breast cancer	HER2	IgG1k	Enzyme cleavable	MMAE	Tubulin inhibitor	http://www.alteogen.com/en/pipeline_1/
AMT-151	Multitude Therapeutics, USA	Solid tumor l Gynecologic cancer l Lung cancer l Breast cancer l Pancreatic Cancer	FRα	...	...	...	Potentially leads to decreased proliferation of tumor cells expressing FRa	https://www.multitudetherapeutics.info/portfolio
Anetumab Ravtansine | BAY 94-9343	Bayer, Germany	Lung adenocarcinoma l Malignant pleural mesothelioma l Ovarian Cancer	Mesothelin	IgG1	Reducible disulfide linker	DM4	Tubulin inhibitor	https://www.pharmaceutical-technology.com/data-insights/anetumab-ravtansine-bayer-ovarian-cancer-likelihood-of-approval/

ARX517	Ambrx, USA	Prostate Cancer	PSMA	IgG1κ	Non-cleavable PEG linker	AS269	Microtubule inhibitor	https://ambrx.com/pipeline/
ARX788	Ambrx, USA	Breast cancer l Gastric cancer	HER2	Trastuzumab-based antibody	Non-cleavable linker	AS269	Microtubule inhibitor	https://ambrx.com/pipeline/
ASN-004 (ASN004)	Asana BioSciences, USA l Mersana Therapeutics, USA	Solid tumors	5T4	scFvFc antibody	Cleavable linker	Dolaflexin	Microtubule disruptor	https://www.asanabiosciences.com/pipeline
ATG-022	Antengene, China	Solid tumors	Claudin 18.2	IMAB362	Enzyme cleavable linker	MMAE	Immunomodulator	https://www.antengene.com/research.html
AVP04	Avipep Therapeutics, Australia	Adenocarcinoma	…	…	…	…	…	http://avipep.com/technology/pipeline/
AZD5335	AstraZeneca, UK	Ovarian cancer l Lung cancer	FRα	…	Cleavable linker	AZ14170132	Topoisomerase 1 inhibitor	https://clinicaltrials.gov/ct2/show/NCT05797168?term=AZD5335&draw=2&rank=1
AZD8205	AstraZeneca, UK	Solid tumors	B7H4	…	Peptide linkers	AZ14170133	Topoisomerase 1 inhibitor	https://www.astrazeneca.com/our-therapy-areas/pipeline.html

AZD9592	AstraZeneca, UK	Solid tumors l Non-small cell lung cancer l Head and neck cancer	cMET/ EGFR	IgG	Cleavable linker	AZ14170133	Topoisomerase 1 inhibitor	https://www.astrazeneca.com/content/dam/az/PDF/2022/Q3/Year-to-date_and_Q3_2022_results_presentation.pdf
BA1301	Luye Pharma Group, China	Gastric cancer l Pancreatic cancer l Esophageal cancer	Claudin 18.2	…	…	…	Tubulin polymerisation inhibitors	https://www.luye.cn/lvye_en/innovate.php#zycpx
BAT8010	Bio-Thera Solutions, China	Solid tumors	HER2	Humanized anti-HER2 antibody	Cleavable linker	Topoisomerase I inhibitor	Topoisomerase 1 inhibitor	https://www.bio-thera.com/plus/list.php?tid=54
BAT8006	Bio-Thera Solutions, China	Solid tumors	FRα	Humanized anti-FRα antibody	Cleavable linker	Exatecan	Topoisomerase 1 inhibitor	https://www.bio-thera.com/plus/list.php?tid=54

BAT8007	Bio-Thera Solutions, China	Solid tumors	Nectin-4	Humanized anti-nectin antibody	Cleavable linker	Topoisomerase I inhibitor	Topoisomerase 1 inhibitor	https://www.bio-thera.com/plus/list.php?tid=54
BAT8008	Bio-Thera Solutions, China	Solid tumors	TROP2	...	Cleavable linker	Topoisomerase I inhibitor	Topoisomerase 1 inhibitor	https://www.bio-thera.com/plus/list.php?tid=54
BAT8009	Bio-Thera Solutions, China	Solid tumors	B7H3	Anti-B7-H3 antibody	Cleavable linker	Exatecan	Topoisomerase 1 inhibitor	https://www.bio-thera.com/plus/list.php?tid=54
BB-1701 | HER2-Targeting ADC	Bliss Biopharmaceutical, India / Eisai, Japan	Solid tumors	HER2	IgG1κ	Enzyme cleavable	Eribulin	Microtubule disruptor	https://www.blissbiopharma.com/Innovation.html#Pipeline
BB-1705	Bliss Biopharmaceutical, India / Eisai, Japan	Solid tumors	EGFR	IgG1κ	Enzyme cleavable	Eribulin	Microtubule disruptor	https://www.blissbiopharma.com/Innovation.html#Pipeline

BDC-1001	Bolt Biotherapeutics, USA	Solid tumors	HER2	Trastuzumab biosimilar	Non-cleavable linker	Imbotolimod	TLR7/8 agonists	https://www.boltbio.com/pipeline-new/
BIO-106	BiOneCure Therapeutics, USA	Solid tumors	TROP2	…	Cleavable linker	TAM	Tubulin inhibitor	https://www.bionecure.com/pipeline/
BL-B01D1	SystImmune, USA	Breast Cancer l Solid tumors l Urinary tumors l Gastric cancer	EGFR/HER3	Izalontamab	Cleavable linker	Ed-04	Topoisomerase 1 inhibitor	https://systimmune.com/pipeline
BL-M02D1	SystImmune, USA	Lung cancer l Breast cancer l Gastrointestinal l Solid tumors	TROP2	Hu4D3	Cleavable linker	Ed-04	Topoisomerase 1 inhibitor	https://systimmune.com/pipeline
BL-M07D1	SystImmune, USA	Breast cancer l Gastric cancer	HER2	Trastuzumab	Cleavable linker	Ed-04	Topoisomerase 1 inhibitor	https://systimmune.com/pipeline
BMS986288 |	Bristol-Myers Squibb,	Solid tumors	CTLA-4	Ipilimumab	Enzyme cleavable	…	Cytotoxic T-lymphocyt	https://www.bms.com/researchers-and-partners/in-the-pipeline.html

BMS-986288	USA l CytomX, USA			probody			e antigen 4 inhibitors	
LM-305	LaNova Medicines, China l AstraZeneca, UK	Multiple Myeloma	GPRC5D	anti-GPRC5D antibody	Enzyme cleavable	MMAE	Tubulin inhibitor	https://www.lanovamedicines.com/en/pipeline
BYON3521	Byondis, Netherlands	Solid tumor	c-MET	SYD2884	Enzyme cleavable	Duocarmycin hydroxybenzamide azaindole	DNA alkylating agent	https://www.byondis.com/pipeline
CMG901	KeyMed, China / Mirocogen, China	Solid tumors l Gastric cancer l Pancreatic cancer	Claudin 18.2	anti-Claudin 18.2 monoclonal antibody	Cleavable linker	MMAE	Complement system protein stimulants; Immunomodulators	https://en.keymedbio.com/list-12-1.html#catid150
Camidanlumab tesirine | ADCT-301 | HuMax-	ADC Therapeutics, Switzerland	Hodgkin Lymphoma	CD25	HuMax-TAC	Enzyme cleavable	PBD-Dimer	Cell death stimulants; DNA cross linking agents	https://www.adctherapeutics.com/our-pipeline/

TAC-ADC								
CC-99712	Bristol-Myers Squibb, USA	Multiple myeloma	BCMA	IgG1κ	Noncleavable linker	Maytansine	Tubulin inhibitor	https://clinicaltrials.gov/ct2/show/NCT04036461
TRPH-222	Catalent, USA	Non-Hodgkin's lymphoma	CD22	Site-specific modified humanized IgG antibody	Noncleavable linker	Maytansine	Tubulin inhibitor	https://www.catalent.com/catalent-news/triphase-accelerator-and-catalent-announce-interim-results-of-a-dose-escalation-phase-1-clinical-trial-of-trph-222-in-patients-with-non-hodgkins-lymphoma/
Cofetuzumab pelidotin | PF-06647020 | ABBV-647	Pfizer, USA	Breast cancer l Non-small cell lung cancer l Ovarian cancer	PTK7	hu6M024, IgG1	Cleavable linker	Aur0101	Microtubule inhibitor	https://cdn.pfizer.com/pfizercom/product-pipeline/Pipeline_Update_04MAY2021_4.pdf?VersionId=K4xD6G3LfgQV9VpdrI1Tf0.WdLQqycb5
CS5001 l ABL202 l LCB71	CStone Pharmaceuticals, China l ABL	Chronic lymphocytic leukemia l Mantle	ROR1	…	Cleavable linker	PBD prodrug	Apoptosis stimulants	http://www.ablbio.com/en/company/pipeline01

	Bio, China	cell lymphoma l Acute lymphocytic leukemia l Breast cancer l Lung cancer						
CX-2029 l ABV-2029	CytomX Therapeutics, USA l AbbVie, USA	Solid tumor l Head and neck cancer l Non-small cell lung cancer l Diffuse large B cell lymphoma l Esophageal cancer	CD71	Masked form (pb-tx) of a proprietary anti-CD71 antibody	Cleavable linker	MMAE	Microtubule inhibitor	https://cytomx.com/pipeline/

Datopotamab deruxtecan | Dato-Dxd l DS-1062s	Daiichi Sankyo, Japan	Solid tumors l Non-small cell lung cancer l Endometrial cancer l Gastric cancer l Prostate cancer l Ovarian cancer l Colorectal cancer l Breast cancer	TROP2	Datopotamab	Tetrapeptide-based linker	Deruxtecan	Topoisomerase I inhibitor	https://www.daiichisankyo.com/rd/pipeline/#anc01
DB-1202	Duality Biologics, China	Solid tumors	...	...	...	...	...	https://clinicaltrials.gov/ct2/show/NCT05785728?term=DB-1202&draw=2&rank=1
DB-1303	Duality Biologics, China	Breast cancer l Gastric cancer l Endometrial cancer l Biliary tract	HER2	IgG1	Enzyme cleavable	P1003	Topoisomerase I inhibitor	https://www.dualitybiologics.com/pipeline.html

		cancer l Solid tumor l Uterine cancer						
DB-1305	Duality Biologics, China	Breast cancer l Non-small cell lung cancer l Colorectal cancer l Endometrial cancer l Gastric cancer l Lung cancer	TROP2	IgG1	Cleavable linker	P1021	Topoisomerase I inhibitor	https://www.dualitybiologics.com/pipeline.html
DB-1310	Duality Biologics, China	Breast cancer l Head and neck tumor l Non-small-cell lung cancer l Prostat	HER3	IgG1	Cleavable linker	Topoisomerase I inhibitor	Topoisomerase I inhibitor	https://www.dualitybiologics.com/pipeline.html

		e cancer						
Disitamab Vedotin l RC48	RemeGen, China	Gastric cancer l Bladder cancer l Breast cancerl Liver cancer l Gyncologic cancer l Lung cancer l Melanoma	HER2	Hertuzumab	Cleavable linker	MMAE	Tubulin polymerization inhibitor	http://www.remegen.com/?v=listing&cid=92#cpgx
DP303c	CSPC ZhongQi Pharmaceutical, China	Breast cancer l Ovarian cancer l Solid tumors l Gastric cancer	HER2	IgG1 (DP001)	Cleavable linker	MMAE	Tubulin polymerization inhibitor	https://clinicaltrials.gov/ct2/show/NCT05334810?term=DP303c&draw=2&rank=1
DS-6000a	Daiichi Sankyo, Japan	Renal Cell Carcinoma l Ovarian cancer	CDH6	IgG1	Cleavable linker	DX8951	Topoisomerase I inhibitor	https://www.daiichisankyo.com/rd/pipeline

DS-9606a	Daiichi Sankyo, Japan	Solid tumors l Prostate cancer l Melanoma l Pancreatic cancer l Hepatocellular cancer l Ovarian cancer l Renal cell carcinoma	Claudin-6	...	...	PDB dimer	...	https://ClinicalTrials.gov/show/NCT05394675 https://xueqiu.com/2874269426/221931800
DXC-007	Hangzhou Duoyi Biotechnology	Acute myeloid leukemia	CD33	...	...	Tub255	Tubulin inhibitor	http://www.dacbiotech.com/show-132-230-1.html
DX126-262 l DAC-001	Hangzhou Duoyi Biotechnology	Breast cancer l Gastric cancer	HER2	DX-CHO9	Cysteine linked	TUB114	Tubulin polymerization inhibitor	http://www.dacbiotech.com/en/show-142-191-1.html

EBC-129	Experimental Drug Development Centre, Singapore/ A*STAR research entities, Singapore	Solid tumors	CEACAM 5/6	...	Cleavable linker	MMAE	CEACAM inhibitor, n glycosylation binder	https://www.geneonline.com/first-made-in-singapore-antibody-drug-conjugate-approved-to-enter-clinical-trials/
F0002 | CD30-MCC-DM1	Fudan-Zhangjiang Bio-Pharmaceutical, China	Liquid tumors	CD30	cAC10	Stable SMCC-non cleavable linker	DM1	Tubulin inhibitor	https://clinicaltrials.gov/ct2/show/NCT03894150
EO-3021 l SYSA1801 l CPO102	CSPC ZhongQi Pharmaceutical l Conjupro Biotherapeutics	Solid tumors l Gastric cancer l Gastroesophageal junction cancer l Pancreatic cancer	Claudin 18.2	IgG1	Cleavable linker	MMAE	Tubulin inhibitor	https://clinicaltrials.gov/ct2/show/NCT05009966

ESG401 l STI-3258	Shanghai Escugen Biotechnology Co, China l Sorrento Therapeutics, USA	Breast cancer l Lung cancer l Colorectal cancer l Bladder cancer l Stomach cancer l Ovarian cancer	TROP2	IgG1	Cleavable linker	SN38	Topoisomerase I inhibitor	https://www.globenewswire.com/en/news-release/2023/06/05/2682017/0/en/Shanghai-Escugen-Biotechnology-Co-Ltd-a-partner-of-Levena-Biopharma-a-Sorrento-Company-releases-positive-results-from-a-first-in-human-study-of-ESG401-a-TROP2-Antibody-Drug-Conjuga.html
Farletuzumab Ecteribulin | MORAb-202	Bristol-Myers Squibb, USA	Gynecologic cancer l Gastric cancer l Non-small cell lung cancer l Breast cancer	Fos-related antigen	Farletuzumab	Cathepsin-cleavable linker	Eribulin	Microtubule disruptor	https://classic.clinicaltrials.gov/ct2/show/NCT03386942
FDA-018	Fudan-Zhangjiang Bio-Pharmaceutical, China	Solid tumors	TROP2	IgG	Acid-cleavable linker	SN38	Topoisomerase I inhibitor	https://clinicaltrials.gov/ct2/show/NCT05174637?term=FDA-018&draw=2&rank=1

FDA-022	Fudan-Zhangjiang Bio-Pharmaceutical, China	Solid tumors	HER2	...	...	BB05	topoisomerase I inhibitors	https://clinicaltrials.gov/ct2/show/NCT05564858?term=NCT05564858&draw=2&rank=1
FOR46	Fortis Therapeutics, USA	Multiple Myeloma l Prostate cancer	CD46	23AG2	Cleavable linker	MMAE	Tubulin inhibitor	https://fortistx.com/for46-pipeline/
FS-1502	Shanghai Fosun Pharmaceutical, China	Breast cancer l Gastric Cancer l Non-small cell lung cancer l Colorectal cancer l Solid tumors	HER2	Trastuzumab	Cleavable linker	MMAF	Tubulin inhibitor	https://www.fosunpharma.com/en/innovate/pipeline.html#a01
NBT508	NewBio Therapeutics, China	Non-Hodgkin's lymphoma	CD79b	...	Cleavable linker	MMAE	Tubulin inhibitor	http://www.newbiotherapeutics.com/list/?26_1.html

GB-251 l NBT828	Genor Biopharma, China l NewBio Therapeutics, China	Breast cancer	HER2	IgG1κ	Cleavable linker	MMAE	Tubulin inhibitor	http://www.newbiotherapeutics.com/list/?26_1.html
GQ1001	GeneQuantum Healthcare, China	Breast cancer l Gastric cancer l Solid tumors	HER2	Trastuzumab	Open-ring containing stable linker	DM1	Tubulin inhibitor	http://www.genequantum.com/#/common/product?pid=12&id=13&type=7
HDP-101	Heidelberg Pharma, Germany	Multiple myeloma	BCMA	anti-CD269 mAb	Cleavable linker	Amanitin	DNA-directed RNA polymerase inhibitor	https://heidelberg-pharma.com/en/research-development/portfolio-overview
GQ1005	GeneQuantum Healthcare, China	Breast cancer l Gastric cancer l Solid tumors	HER2	...	...	...	...	http://www.genequantum.com/#/common/product?pid=12&id=13&type=8
HS-20089	Hansoh Pharmaceutical, China	Solid tumors	B7H4	...	...	...	Tubulin inhibitor	https://clinicaltrials.gov/ct2/show/NCT05263479
HS-20093	Hansoh Pharmaceutical, China	Sarcoma l Osteosarcoma l	B7H3	IgG1	Cleavable linker	...	...	https://ascopubs.org/doi/pdf/10.1200/JCO.2023.41.16_suppl.3017?role=tab

		Solid tumor						
HTI-1066 | SHR-A1403	Hansoh Pharmaceutical, China	Pancreatic cancer	c-MET	IgG2	Non-cleavable linker	Auristatin analog SHR152852	Microtubule inhibitor	https://www.frontiersin.org/articles/10.3389/fonc.2021.634881/full
IBI-343	Innovent Biologics, China	Solid tumors	Claudin 18.2	…	…	…	Antibody-dependent cell cytotoxicity	https://www.innoventbio.com/ScienceAndProducts/Pipeline
IBI-354	Innovent Biologics, China	Solid tumors	HER2	…	…	Camptothecin derivative	Topoisomerase I inhibitor	https://www.innoventbio.com/ScienceAndProducts/Pipeline
IKS03 l LCB73	Iksuda, UK l Legochembio, South Korea l Light Chain Bioscience, Switzerland	Non-Hodgkin's lymphoma l Large B cell lymphoma l Follicular lymphoma l Acute lymphocytic	CD19	Anti-CD19	Enzyme cleavable (glucuronide-trigger technology)	PBD dimer pro-drug	DNA replication blocker	https://iksuda.com/pipeline/ https://www.legochembio.com/pipeline/pipeline.php?lang=k

		leukemia						
Ifinatamab Deruxtecan - DS-7300a	Daiichi Sankyo, Japan	Solid tumors	B7H3	IgG1κ	Enzyme cleavable	MAAA1181a	Topoisomerase I inhibitor	https://www.daiichisankyo.com/files/investors/library/quarterly_result/2021/FY2021%20Q2%20Presentation%20Material.pdf
IMGC936	Immunogen, USA l MacroGenics, USA	Non-small cell lung cancer l Gastric cancer l Pancreatic cancer l Breast cancer l Solid tumors	ADAM9	MGA021	Tripeptide linker	DM21C	Microtubule inhibitor	https://www.immunogen.com/what-we-do/our-pipeline/
IMGN151	Immunogen, USA	Gynecologic cancer l Non-small cell lung cancer l	FRα	B5327A	Tripeptide linker	DM21	Microtubule inhibitor	https://www.immunogen.com/what-we-do/our-pipeline/

		Breast cancer						
INA03	Inatherys, France	Leukemia	CD71	IgG4	...	MMAE	Microtubule inhibitor	https://ascopubs.org/doi/abs/10.1200/JCO.2023.41.16_suppl.7045?af=R
JBH492	Novartis, Switzerland	Non-Hodgkin's Lymphoma l Chronic lymphocytic leukemia	CCR7	IgG1	Cleavable linker	DM4	Microtubule inhibitor	https://clinicaltrials.gov/ct2/show/NCT04240704
JS107	Shanghai Junshi Bioscience, China	Pancreatic cancer l Gastric cancer	Claudin 18.2	...	Cleavable linker	MMAE	Microtubule inhibitor	https://www.junshipharma.com/en/rd-pipeline/#medical-content
JS108	Shanghai Junshi Bioscience, China	Solid tumor	TROP2	Anti-Trop2 mAb	...	Tub196	Tubulin inhibitor	https://clinicaltrials.gov/ct2/show/NCT04601285?term=JS108&draw=2&rank=1
JSKN003	Jiangsu Alphamab Biopharmaceuticals, China	Solid tumor	HER2	KN026	Dibenzocyclooctyne tetrapeptide linker	Topoisomerase I inhibitor	topoisomerase inhibitor	https://www.alphamabonc.com/en/pipeline/jskn003.html

Ladiratuzumab vedotin | SGN-LIV1A | Anti-LIV-1 ADC	Seagen, USA l Merck, USA	Breast Cancer l Solid tumors	LIV1	IgG1κ	Enzyme cleavable	MMAE	Microtubule inhibitor	https://www.seagen.com/science/pipeline
LCB14-0110	Legochembio, South Korea	Breast cancer l Gastric cancer l Ovarian cancer l Lung cancer l Colorectal cancer	HER2	Trastuzumab	LegoChem's proprietary linker	MMAF	Tubulin inhibitor	https://www.legochembio.com/pipeline/pipeline.php?lang=k
LM-302 l BMS-986476	Bristol-Myers Squibb, USA l LaNova Medicines, China	Solid tumors	Claudin 18.2	IgG1	Cleavable linker	MMAE	Microtubule inhibitor	https://www.bms.com/researchers-and-partners/in-the-pipeline.html

STRO-001 l BN301	Sutro Biopharma, USA l Bionova Pharma, USA	Non Hodgkin's lymphoma l Multiple myeloma l Follicular lymphoma l Mantle cell lymphoma l Indolent lymphoma	CD74	SP7219	Non-cleavable dibenzocyclooctyne (DBCO) linker	Maytansinoid	Tubulin inhibitor	https://www.sutrobio.com/pipeline/
CC-99712	Sutro Biopharma, USA l BMS, USA	Multiple myeloma	BCMA	IgG1κ	Non-cleavable linker	Maytansinoid	Tubulin inhibitor	https://www.sutrobio.com/pipeline/
Luveltamab Tazevibulin l STRO-002 l	Sutro Biopharma, USA	Gynecologic cancer l Non-small cell	FR1α	IgG1	Cleavable drug linker, SC239	SC209	Tubulin inhibitor	https://www.sutrobio.com/pipeline/

Luvelta		lung cancer						
M1231	Sutro Biopharma, USA l EMD Serono, USA l Merck, USA	Non-small cell lung cancer l Esophageal cancer	MUC1-EGFR	anti-MUC1/EGFR bispecific antibody	Cleavable linker	Hemiasterlin	Tubulin inhibitor	https://www.sutrobio.com/pipeline/
M9140	EMD Serono, USA	Colorectal cancer l Solid tumors	CEACAM5	…	…	Exatecan	Topoisomerase I inhibitor	https://classic.clinicaltrials.gov/ct2/show/NCT05464030
Mecbotamab Vedotin | BA3011 | CAB-Axl-ADC	BioAtla, USA	Solid tumors l Non-small cell lung cancer l Melanoma l Sarcoma	AXL	IgG1	Cleavable linker	MMAE	Tubulin inhibitor	https://www.bioatla.com/cab-portfolio/
MEN1309 | OBT076	Menarini Ricerche, Italy/ Oxford BioTher	Solid tumors	CD205	IgG2	Cleavable linker	DM4	Tubulin inhibitor	https://www.menarini.com/en-us/innovation-research/our-pipeline-and-products

	apeutics, UK							
MHB 036C	Minghui Pharmaceutical, China	Solid tumors	TROP2	…	Cleavable linker	…	Topoisomerase I inhibitor	https://www.biospace.com/article/releases/minghui-pharmaceutical-inc-announces-first-patient-dosing-in-phase-1-clinical-studies-of-both-the-antibody-drug-conjugate-programs-targeting-trop-2-or-b7-h3-respectively-for-treatment-of-advanced-or-metastatic-solid-tumors/
MHB 088C	Minghui Pharmaceutical, China	Solid tumors	B7H3	NM8074	Cleavable linker	…	Topoisomerase I inhibitor	https://www.biospace.com/article/releases/minghui-pharmaceutical-inc-announces-first-patient-dosing-in-phase-1-clinical-studies-of-both-the-antibody-drug-conjugate-programs-targeting-trop-2-or-b7-h3-respectively-for-treatment-of-advanced-or-metastatic-solid-tumors/
Mirzotamab Clezutoclax | ABBV-155	AbbVie, USA	Non-small cell lung cancer l Breast cancer	CD276	Mirzotamab	Peptide cleavable linker	Clezutoclax	BCL2 family protein inhibitor	https://clinicaltrials.gov/ct2/show/NCT03595059?term=abbv-155&draw=2&rank=1
MK-2870	Merck, USA l Sichuan Kelun-Biotech, China	Solid tumor	TROP2	…	…	Belotecan derivative	Topoisomerase inhibitor	https://www.merck.com/research/product-pipeline/

MRG 002	Miracogen, China	Breast cancer l Liver cancer l Non-small-cell lung cancer l Urothelial cancer l Biliary tract cancer l Gastric Cancer	HER2	Trastuzumab	Cleavable linker	MMAE	Tubulin inhibitor	http://www.miracogen.com.cn/en/Index/lists/catid/9.html
MRG 001	Miracogen, China	Non-Hodgkin's lymphoma	CD20	Chimeric anti-CD20 monoclonal antibody	Cleavable linker	MMAE	Tubulin inhibitor	http://www.miracogen.com.cn/en/Index/lists/catid/9.html
MRG 003	Miracogen, China	Gastric cancer l Head and Neck cancer l Biliary tract	EGFR	IgG1	Cleavable linker	MMAE	Tubulin inhibitor	http://www.miracogen.com.cn/en/Index/lists/catid/9.html

		cancer l Non-small-cell lung cancer l Nasopharyngeal carcinoma						
MRG 004A	Miracogen, China	Solid tumors	Tissue Factor	..	Enzyme cleavable	MMAE	Tubulin inhibitor	http://www.miracogen.com.cn/en/Index/lists/catid/9.html
Naratuximab emtansine | IMGN 529 | K7153A | Debio 1562	Debiopharm, Switzerland	Non-Hodgkin's lymphoma l Marginal zone lymphoma l Follicular lymphoma l Acute myeloid leukemia	CD37	K7153A	Non-cleavable linker	DM1	Tubulin polymerization inhibitor	https://www.debiopharm.com/pipeline/debio-1562/
MT-8633	Mitsubishi	Solid tumor	cMET	IgG2	Enzyme cleavable	PBD moiety	Replication inhibitor	https://www.mt-pharma.co.jp/e/develop/pipeline.html

l TR1801	Tanabe Pharma							
MYTX-011	Mythic Therapeutics, USA	Non-small cell lung cancer	cMET	IgG1	cleavable linker	MMAE	Tubulin inhibitor	https://mythictx.com/clinical-program/
NBE-002 l BI3702025	Boehringer Ingelheim, Germany/ NBE Therapeutics Switzerland	Breast cancer l Lung cancer l Gynecologic cancer	ROR1	IgG1	non-cleavable linker	PNU-159682	DNA intercalator l Type II DNA topoisomerase inhibitor	https://nbe-therapeutics.com/pipeline
OBI-999 | Anti-Globo H ADC	OBI-Pharma, Taiwan	Solid tumors	Globo H	IgG1 (OBI 888)	cleavable linker	MMAE	Tubulin inhibitor	https://www.obipharma.com/pipeline/
OMTX705	Oncomatryx, Spain	Pancreatic cancer l Breast cancer l Lung cancer	FAP α	OMTX005	cleavable linker	Tubulysin TAM470	Tubulin polymerization inhibitor	https://oncomatryx.com/adc-omtx705/
ORM-5029	Orum Therapeutics,	Breast Cancer	HER2	Pertuzumab	cleavable linker	SMol006	GSPT1 Degrader	https://docsend.com/view/dqym6ks36t73qz7k

	South Korea							
Ozuriftamab Vedotin | BA3021 | Anti-ROR2 ADC | CAB-ROR2-ADC	BioAtla, USA	Non-small cell lung cancer l Melanoma l Ovarian cancer l Head and Neck cancer	ROR 2	Ozuriftamab	cleavable linker	MMAE	Tubulin inhibitor	https://www.bioatla.com/cab-portfolio/
Pivekimab Sunirine | IMGN632 | IMGN632	Immunogen, USA	Acute myeloid leukemia l Blastic plasmacytoid dendritic cell neoplasm	CD123	IgG1	cleavable linker	Indolinobenzodiazepine pseudodimer	DNA alkylation	https://pubmed.ncbi.nlm.nih.gov/29661755/
Patritumab Deruxtecan l U3-1402	Daiichi Sankyo, Japan/ Amgen, USA	Non-small cell lung cancer l Breast cancer l Brain	HER 3	Patritumab	Peptide cleavable linker	DX-8951	Toposisomerase I inhibitor	https://www.daiichisankyo.com/rd/pipeline/#anc01

		metastasis						
Praluzatamab Ravtansine | CX-2009	CytomX Therapeutics, USA/ AbbVie, USA	Breast cancer l Non small cell lung cancer l Head and neck cancer l Gynecologic cancer	CD166	IgG1κ	cleavable linker	DM4	Tubulin inhibitor	https://clinicaltrials.gov/ct2/show/NCT03149549?term=CX-2009&draw=2&rank=1
PRO1160	ProfoundBio, China	Renal Cell Carcinoma l Nasopharyngeal l carcinoma l Non Hodgkin's lymphoma	CD70	...	cleavable linker	Exatecan	Topoisomerase I inhibitor	https://profoundbio.com/Pipeline
PRO1184	ProfoundBio, China	Gynecological cancer l Non-	FRα	...	cleavable linker	Exatecan	Topoisomerase I inhibitor	https://profoundbio.com/Pipeline

		small cell lung cancer l Mesothelioma l Breast cancer						
PYX-201	Pyxis Oncology, USA	Breast cancer l Head and neck cancer l Thyroid cancer	Extradomain B fibronectin	IgG1	cleavable linker	Aur0101	Tubulin inhibitor	https://pyxisoncology.com/portfolio/#adc
RC108	RemeGen, China	Solid tumors l Lung cancer l Gastric cancer	cMET	...	cleavable linker	MMAE	Tubulin inhibitor	http://www.remegen.com/?v=listing&cid=92#cpgx
RC118	RemeGen, China	Solid tumors	Claudin 18.2	...	Enzyme cleavable linker	MMAE	Tubulin inhibitor	http://www.remegen.com/?v=listing&cid=92#cpgx
RC88	RemeGen, China	Mesothelioma l Bile duct cancer l Pancreatic cancer l Ovarian	Mesothelin	...	Enzyme cleavable linker	MMAE	Tubulin inhibitor	http://www.remegen.com/?v=listing&cid=92#cpgx

		cancer l Lung cancer l Solid tumors						
REGN 5093	RemeGen, China	Lung cancer	cMET l CMET	IgG4p	Cleavable linker	M114	Proto oncogene protein c met inhibitor	https://ascopubs.org/doi/abs/10.1200/JCO.2022.40.16_suppl.TPS8593
SGN-ALPV	Seagen, USA	Ovarian cancer l l Lung cancer l Stomach cancer l Gynecologic cancer l Testicular cancer	ALPP / ALPPL2	IgG1	Cleavable linker	MMAE	Microtubule disruptor	https://seagenmedicalaffairs.com/pipeline/sgn-alpv-v5-english
SGN-B6A	Seagen, USA	Lung cancer l Head and Neck cancer l Breast cancer l Esophageal cancer l	Integrin β6	H2A2	Cleavable linker	MMAE	Microtubule disruptor	https://seagenmedicalaffairs.com/pipeline/sgn-b6a-v8-english

		Gastric cancer l Gynecologic cancer l Pancreatic cancer l Bladder cancer						
SGN-B7H4V	Seagen, USA	Gynecologic cancer l Breast cancer l Non-Small-Cell Lung cancer l Cholangiocarcinoma l Gallbladder Carcinoma l Adenoid Cystic Carcinoma	B7H4	IgG1	Cleavable linker	MMAE	Microtubule disruptor	https://seagenmedicalaffairs.com/pipeline/sgn-b7h4v-v6-english

SGN-PDL1V	Seagen, USA	Lung cancer l Head and Neck cancer l Esophageal cancer l Gynecologic cancer l Melanoma l Breast cancer	PD-L1	IgG2	Cleavable linker	MMAE	Microtubule disruptor	https://seagenmedicalaffairs.com/pipeline/sgn-pdl1v-v5-english
SGN-STNV	Seagen, USA	Lung cancer l Breast cancer l Gynecologic Cancer l Esophageal Neoplasms l Gastric Cancer l Colorectal Cancer l Pancreatic	STn	H2G12	Cleavable linker	MMAE	Microtubule disruptor	https://seagenmedicalaffairs.com/pipeline/sgn-stnv-v10-english

		Cancer l Appendix Cancer						
SHR-A1811 l Trastuzumab rezetecan	Seagen, USA l Hengrui, China	Breast cancer l Lung cancer l Gastric cancer l Colorectal Cancer l Solid tumor	HER2	IgG1k	Cleavable linker	Rezetecan	DNA replication inhibitor	https://www.hengrui.com/en/pipeline.html
SHR-A1904	Seagen, USA l Hengrui, China	Solid tumors l Pancreatic tumors	Claudin 18.2	IgG1k	...	...	...	https://www.hengrui.com/en/pipeline.html
SHR-A1921	Seagen, USA l Hengrui, China	Solid tumors	TROP2	IgG1	Cleavable linker	Toposisomerase I inhibitor	Toposisomerase I inhibitor	https://www.hengrui.com/en/pipeline.html
SHR-A2009	Seagen, USA l Hengrui, China	Solid tumors	HER3	...	...	...	...	https://www.hengrui.com/en/pipeline.html

SHR-A2102	Seagen, USA l Hengrui, China	Solid tumors	Nectin-4	…	…	…	…	https://www.hengrui.com/en/pipeline.html
SHR-A1912	Seagen, USA l Hengrui, China	B-cell lymphoma	CD79b	…	…	…	…	https://www.hengrui.com/en/pipeline.html
SKB264	Klus Pharma, USA	Solid tumors	TROP2			KL610023		https://www.kluspharma.com/pipeline
SKB315	Klus Pharma, USA	Solid tumors	Claudin 18.2	…	stable linker	…	DNA topoisomerase I inhibitor	https://www.kluspharma.com/pipeline
SOT102	Sotio, Czech Republic l NBE Therapeutics, Switzerland	Gastric Cancer l Pancreatic Cancer	Claudin 18.2	IgG1	Non-cleavable	PNU-159682	DNA replication inhibitor	https://sotio.com/pipeline/antibody-drug-conjugates/sot102
STI-6129	Sorrento Therapeutics, USA	Amyloidosis l Multiple Myeloma l T-ALL l Esophageal cancer	CD38	STI-5171	A non-polyethylene glycol linker	Duostatin	Microtubule inhibitor	https://sorrentotherapeutics.com/research/pipeline/

SYD985 l Trastuzumab Duocarmazine	Byondis, Netherlands	Breast cancer l Endometrial cancer	HER2	Trastuzumab	Cleavable linker	Duocarmycin hydroxybenzamide azaindole	alkylating agent	https://www.byondis.com/pipeline
Telisotuzumab Vedotin | ABBV399 | Teliso-V | ABT-700-vcMMAE	AbbVie, USA	Non-small cell lung cancer	cMET	telisotuzumab	Cleavable linker	MMAE	Microtubule inhibitor	https://classic.clinicaltrials.gov/ct2/show/NCT03539536
TORL123	TORL Biotherapeutics, USA	Gynecologic cancer l Endometrial cancer l Non-small cell lung cancer	Claudin6	...	Cleavable linker	MMAE	Microtubule inhibitor	https://www.prnewswire.com/news-releases/torl-biotherapeutics-announces-initial-results-of-novel-claudin-6-cldn6-targeted-antibody-drug-conjugate-adc-torl-1-23-selected-for-presentation-at-the-2023-american-society-of-clinical-oncology-asco-annual-meeting-301840400.html?tc=eml_cleartime

TORL-2-307	TORL Biotherapeutics, USA	Gastric cancer l Pacreatic cancer	Claudin 18.2	…	Cleavable linker	MMAE	Microtubule inhibitor	https://clinicaltrials.gov/ct2/show/NCT05156866?term=TORL+Biotherapeutics&draw=2&rank=2
TQB2102	Chia Tai Tianqing Pharmaceutical, China l Nanjing Shunxin Pharmaceutical, China	Advanced Cancers	HER2	…	Cleavable linker	…	DNA topoisomerase I inhibitor	https://clinicaltrials.gov/ct2/show/NCT05735496?term=TQB2102&draw=2&rank=1
TRPH-222 (CD22-4AP)	Triphase Accelerator Corporation, Canada	Non-Hodgkin's lymphoma l Acute lymphoid leukemia	CD22	IgG	protease insensitive 4AP linker	Maytansinoid	Tubulin inhibitor	https://clinicaltrials.gov/ct2/show/NCT03682796?term=TRPH-222-100&draw=2&rank=1
TRS005	Teruisi Pharma	Non-Hodgkin's Lymphoma	CD20	…	Cleavable linker	MMAE	Tubulin inhibitor	https://www.teruisipharm.com/products/Working-on-the-product

TQB2103	Chia Tai Tianqing Pharmaceutical, China l Nanjing Shunxin Pharmaceutical, China	Solid tumor	Claudin 18.2	...	enzymatically cleavable linker	DDDXD	DNA topoisomerase I inhibitor	https://clinicaltrials.gov/ct2/show/NCT05867563?term=TQB2103&draw=2&rank=1
Trastuzumab mafodotin l IKS014	Iksuda, UK l LegoChem Biosciences, South Korea	Breast cancer l Gastric cancer	HER2	IgG1	cleavable linker	MMAF	Tubulin inhibitor	https://iksuda.com/pipeline/
Tusamitamab Ravtansine | SAR408701 | SAR 408701 | SAR-408701	Innovent Biologics, China l Sanofi, France	Non-small cell lung cancer	CEACAM5	IgG1κ	SPDB linker [N-succinimidyl 4-(2-pyridyldithio)butanoate]	DM4	Tubulin polymerization inhibitor	https://www.sanofi.com/en/media-room/press-releases/2022/2022-08-04-15-30-00-2492626

Upifitamab Rilsodotin	Mersana Therapeutics, USA	Ovarian cancer	Sodium-dependent phosphate transport protein 2B	IgG1κ	Cleavable linker	Auristatin F-hydroxypropylaminde	Microtubule inhibitor	https://www.mersana.com/pipeline/xmt-1536/
Vobramitamab Duocarmazine	MacroGenics, USA	Prostate cancer l Melanoma l Pancreatic cancer l Hepatocellular cancer l Gynecologic cancer l Renal cell cancer	B7H3	IgG1κ	Cleavable linker	DUBA	DNA damaging	https://macrogenics.com/pipeline/
W0101	Pierre Fabre, France	Lung cancer l Head and	IGF-1R	hz208F2-4	non-cleavable	Auristatin derivative	Apoptosis stimulant	https://aacrjournals.org/mct/article/19/1/168/274175/Efficacy-of-the-Antibody-Drug-Conjugate-W0101-in

		neck cancer l Breast cancer						
XB002	Exelixis, USA l Zymeworks, Canada	Non-small cell lung cancer l Cervical cancer l Head and neck cancer l Pancreatic cancer l Esophageal cancer l Prostate cancer l Breast cancer l Gynecologic cancer	Tissue factor	…	Cleavable Linker	Auristatin F	Tubulin polymerization	https://www.exelixis.com/pipeline/
XMT-1660	Mersana Therapeutics, USA	Breast cancer l Gynecologic cancer	B7H4	IgG1	Cleavable Linker	Auristatin derivative	Microtubule inhibitor	https://www.mersana.com/xmt-1660/

XZP-KM501	Sihuan Pharmaceutical, China	Solid tumor l Breast cancer	HER2 domain II l HER2 domain IV	A biparatopic (targeting two different non-overlapping epitopes on ERBB2, on extracellular domains 2 (ECD2) and 4 (ECD4))	...	MMAE	Tubulin inhibitor	https://www1.hkexnews.hk/listedco/listconews/sehk/2023/0324/2023032400211.pdf
YL201	MediLink Therapeutics, China	Solid tumors	...	IgG1	Cleavable Linker	Camptothecin derivative YL0010014	Topoisomerase 1 inhibitor	https://www.medilinkthera.com/pipeline

YL202	MediLink Therapeutics	Solid tumors	HER3	...	Protease cleavable linker	...	Topoisomerase 1 inhibitor	https://www.medilinkthera.com/pipeline l https://aacrjournals.org/cancerres/article/83/7_Supplement/563/722958/Abstract-563-Preclinical-development-of-YL202-a
Zanidatamab Zovodotin | ZW49	Zymeworks, Canada /BeiGene, USA	Solid tumors l Gastric cancer l Breast cancer	HER2 domain II l HER2 domain IV	A biparatopic (targeting two different non-overlapping epitopes on ERBB2, on extracellular domains 2 (ECD2) and 4 (ECD4))	Cleavable Linker	N-acyl sulfonamide auristatin	Microtubule inhibitor	https://www.zymeworks.com/pipeline/

Zilovertamab vedotin | VLS-101 | MK-2140	Merck, USA	Leukemia l Lymphoma l Breast cancer l Ovarian cancer l Gastric Cancer l Pancreatic cancer l Urothelial cancer l Bladder cancer	ROR 1	Zilovertamab	Cleavable Linker	MMAE	Tubulin inhibitor	https://www.merck.com/news/merck-to-acquire-velosbio/
